# Supplementary figures and images for: An integrated analysis of the cancer genome atlas data discovers a hierarchical association structure across thirty three cancer types
Source: PLOS Digit Health. 2022 Dec 20;1(12):e0000151. doi: 10.1371/journal.pdig.0000151 (PMC9931374; doi:10.1371/journal.pdig.0000151)

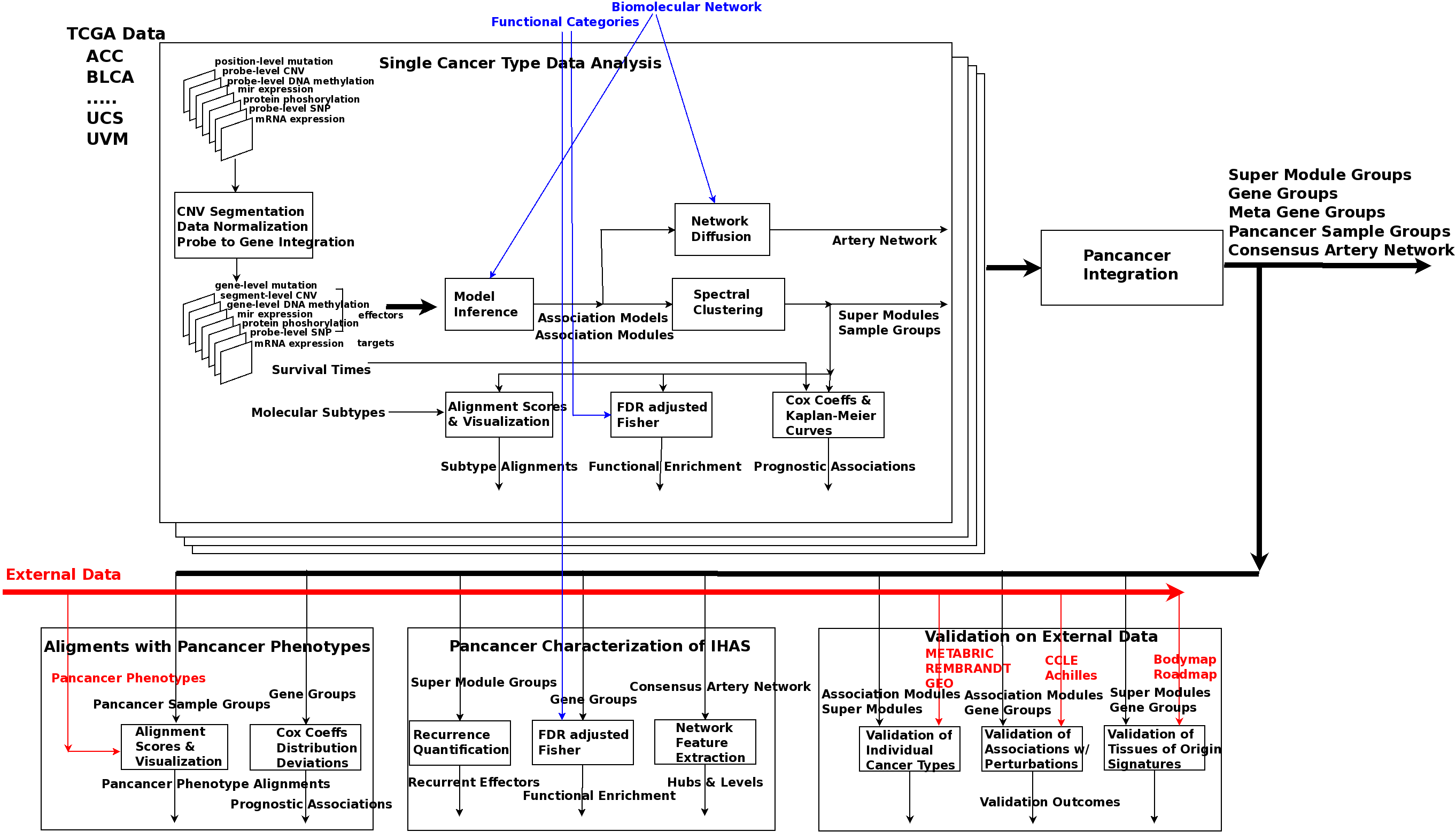

Supplement: S1 Fig — The single cancer type data analysis (the top box) is undertaken for each cancer type separately. In each cancer type the TCGA omics data are processed and fed into the model inference algorithm to build Association Models and Association Modules. The Association Modules and mRNA expression data are bi-clustered to form Super Modules and Sample Groups. The (effector,target) pairs in the Association Models are used to construct the Artery Network from a unified biomolecular network. The Super Modules and Sample Groups then undergo subtype alignments, functional enrichment, and prognostic associations. The inference outcomes of individual cancer types are integrated to form pan-cancer subunits (Super Module Groups, Gene Groups, Consensus Artery Network). These pan-cancer structures are aligned with pan-cancer phenotypes (molecular phenotypes and prognosis), characterized (Recurrent Effectors, functional enrichment, and hubs in the Consensus Artery Network), and validated on external data (individual cancer types, perturbations, normal tissues). Black lines indicate TCGA data and association outcomes. Red lines indicate external data for validation. Blue lines indicate non-TCGA data used to infer IHAS. (TIFF) [file pdig.0000151.s001.tiff]

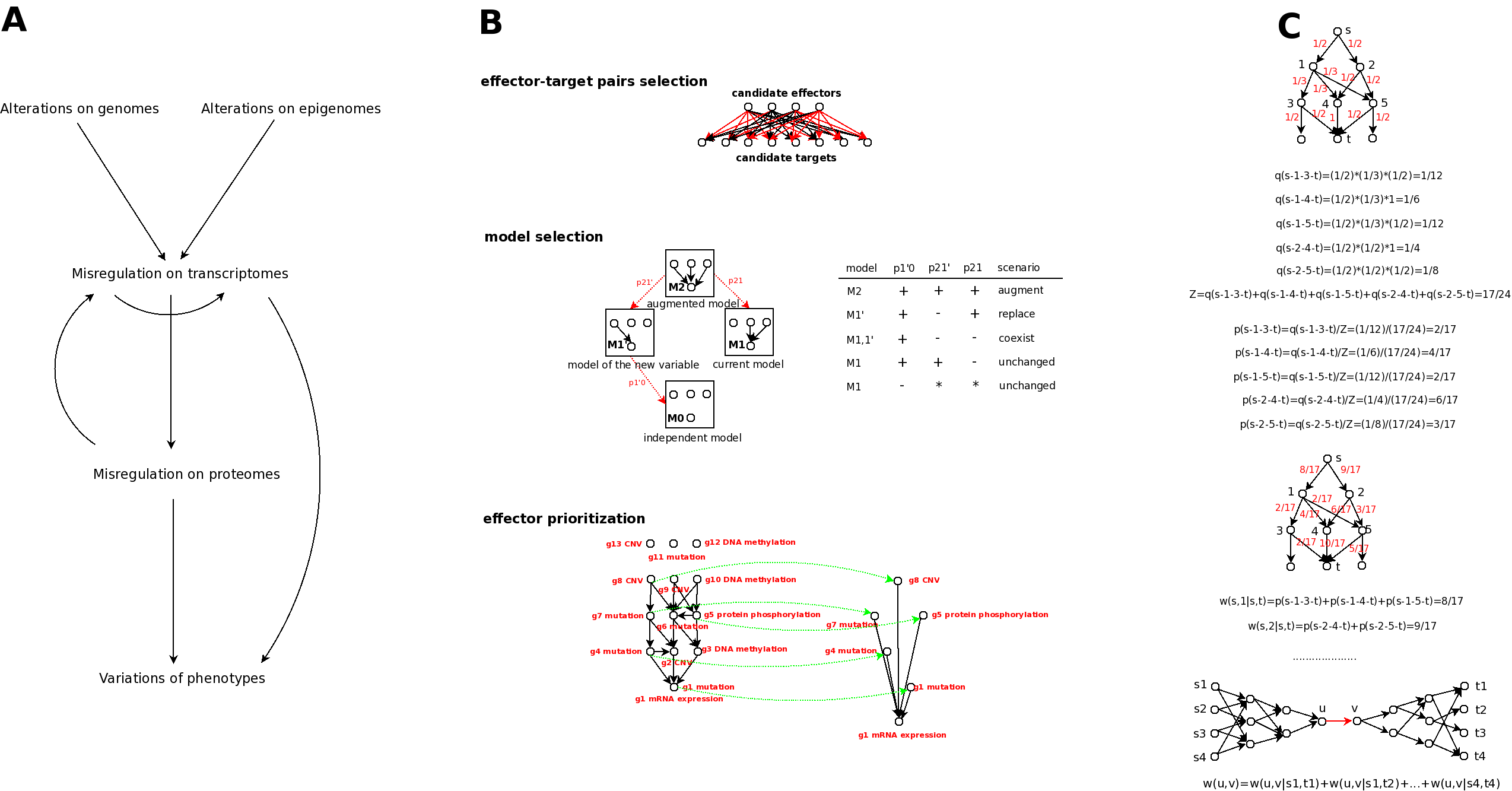

Supplement: S2 Fig — A: Directions of associations according to the central dogma view. Alterations on genomes (sequence mutations, copy number variations, single nucleotide polymorphisms), epigenomes (DNA methylations), microRNA expressions and protein phosphorylations modulate mRNA transcriptions. Variations of mRNA transcriptions modulate protein expressions. Transcriptomic and proteomic variations affect phenotypic variations. B: Procedures of building Association Models of individual genes. First, we calculate pairwise associations between all candidate effectors and targets and select the pairs whose strength of associations (quantified by log-likelihood ratios and permutation p-values) pass threshold values (red edges in the top panel). Second, for each target gene candidate effectors are incrementally selected according to statistical hypothesis test outcomes. In a toy example in the middle panel, suppose we consider merging a candidate effector M1′ with the current model M1 to become the augmented model M2, and M0 is an independent model without effectors. We incur three hypotheses tests comparing pairs of nested models and select models by the three p-values according to the table on the right. Third, before incurring statistical model selection candidate effectors are prioritized by their shortest path lengths to the target gene in the molecular interaction network. Candidate effectors with shorter path lengths have higher priorities. Candidate effectors with the same path lengths are prioritized by their types: CNV > mutation > DNA methylation > microRNA expression > protein phosphorylation > SNP. A toy example in the bottom panel illustrates these priorities. C: Illustration of evaluating edge weights by a network diffusion model. Suppose there is an association between effector s and target t, which are connected by paths in the molecular interaction network. The network diffusion model starts at s, iteratively jumps to downstream neighbor nodes with equal probabilities, a [file pdig.0000151.s002.tiff]

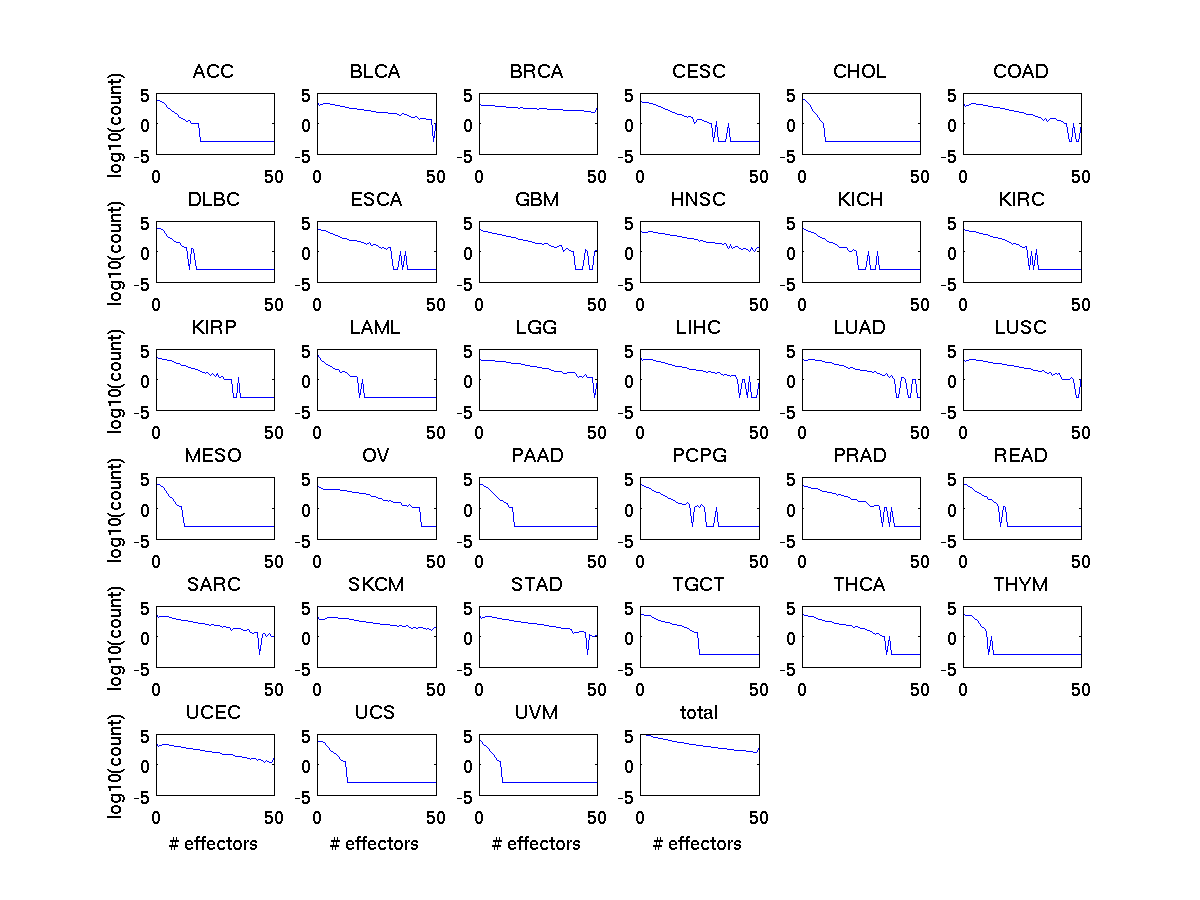

Supplement: S3 Fig — To avoid displaying −∞ (log10 0) values we replaced zero counts with 0.001. (TIF) [file pdig.0000151.s003.tif]

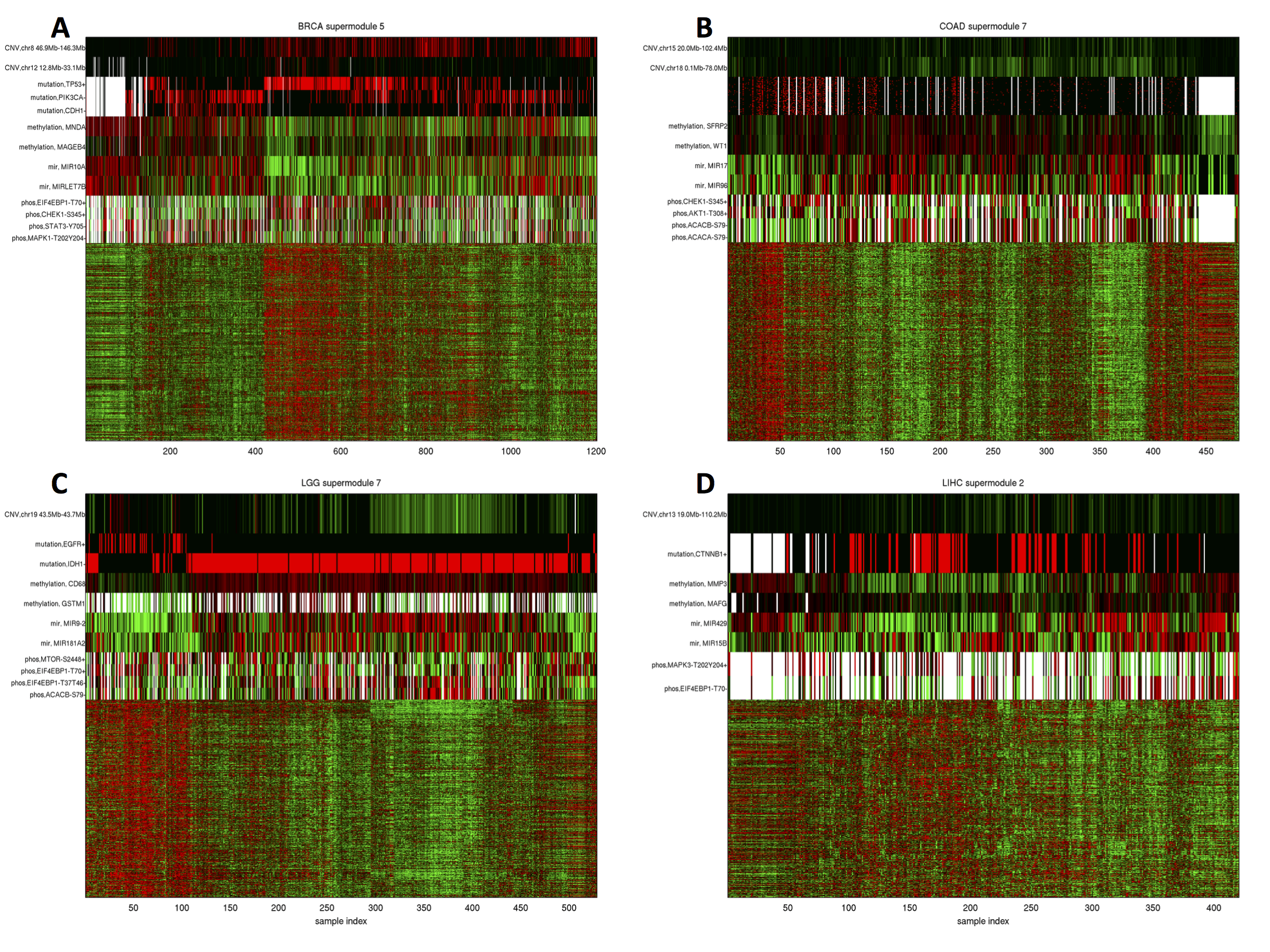

Supplement: S4 Fig — A: BRCA Super Module 5. B: COAD Super Module 7. C: LGG Super Module 7. D: LIHC Super Module 2. Selected effectors are annotated on the upper half rows in each panel. The target genes are on the bottom half rows and not annotated. Color codes follow Fig 3A–3C. (TIFF) [file pdig.0000151.s004.tiff]

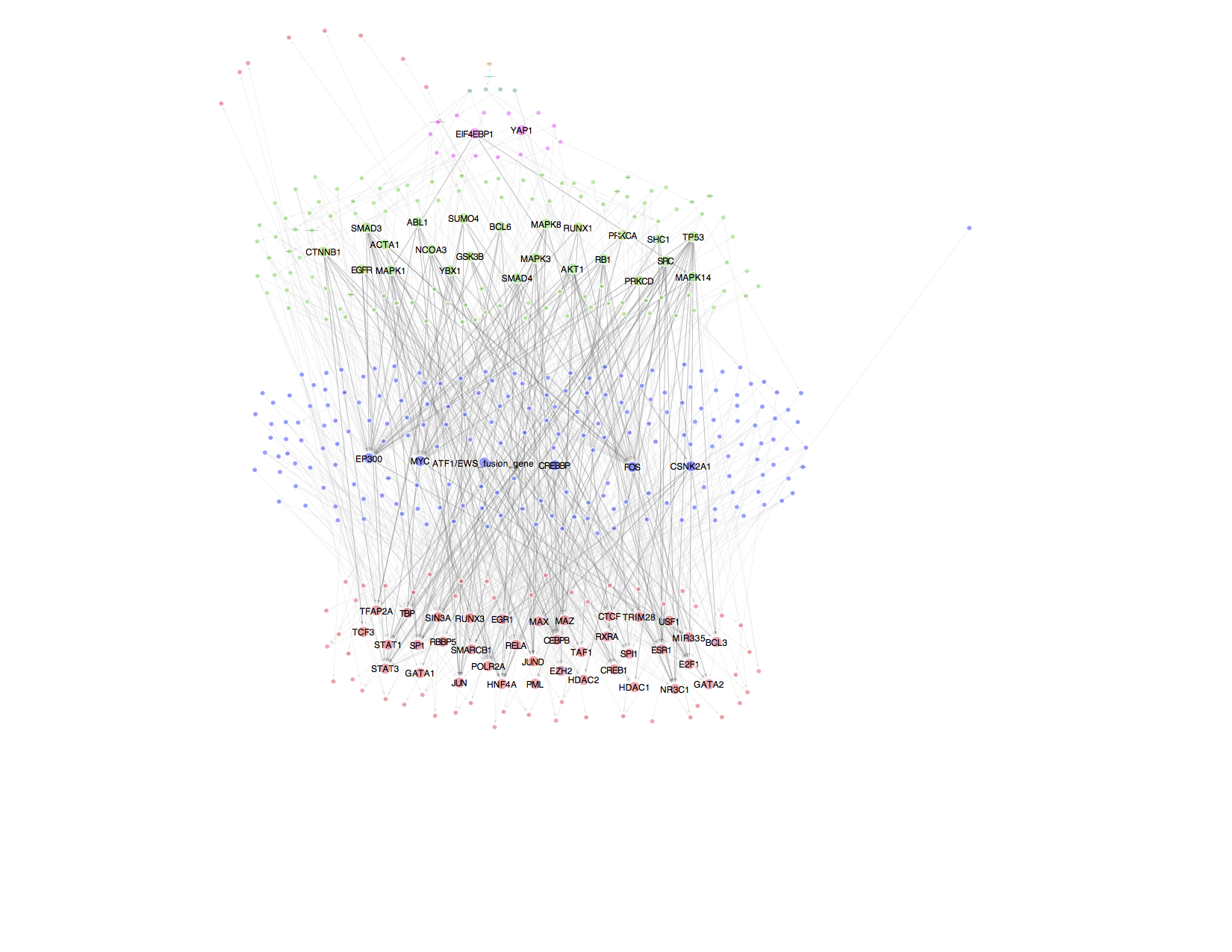

Supplement: S5 Fig — The genes with high connectivity are annotated. Node colors denote hub levels: 1 (red), 2 (purple), 3 (green), 4 (magenta). (TIFF) [file pdig.0000151.s005.tiff]

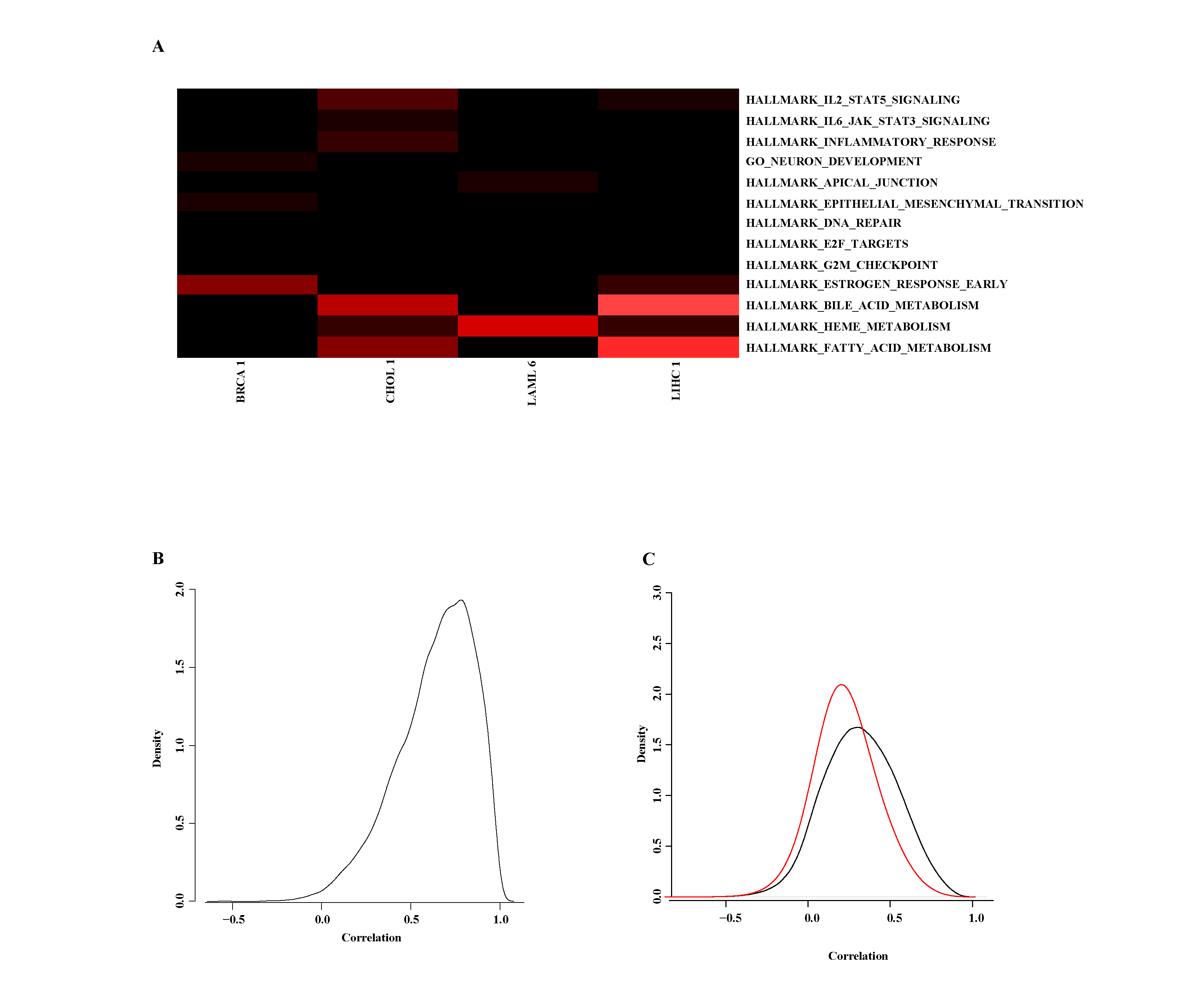

Supplement: S6 Fig — A: Excerpts of four Super Modules enriched with the Gene Sets not belonging to the 18 Gene Groups and their functional enrichment in 13 selected Gene Sets representing the three Meta Gene Groups and several other functional processes. B: Correlation coefficients distribution of the mean target gene expressions between Association Modules and the Super Modules they belong to. C: Correlation coefficients distributions of target gene expressions between the Association Models with identical effectors (black) and the Association Models sharing one common effector but also possessing distinct effectors (red). The y axis in panels B and C indicates values from kernel density estimation. (TIF) [file pdig.0000151.s006.tif]

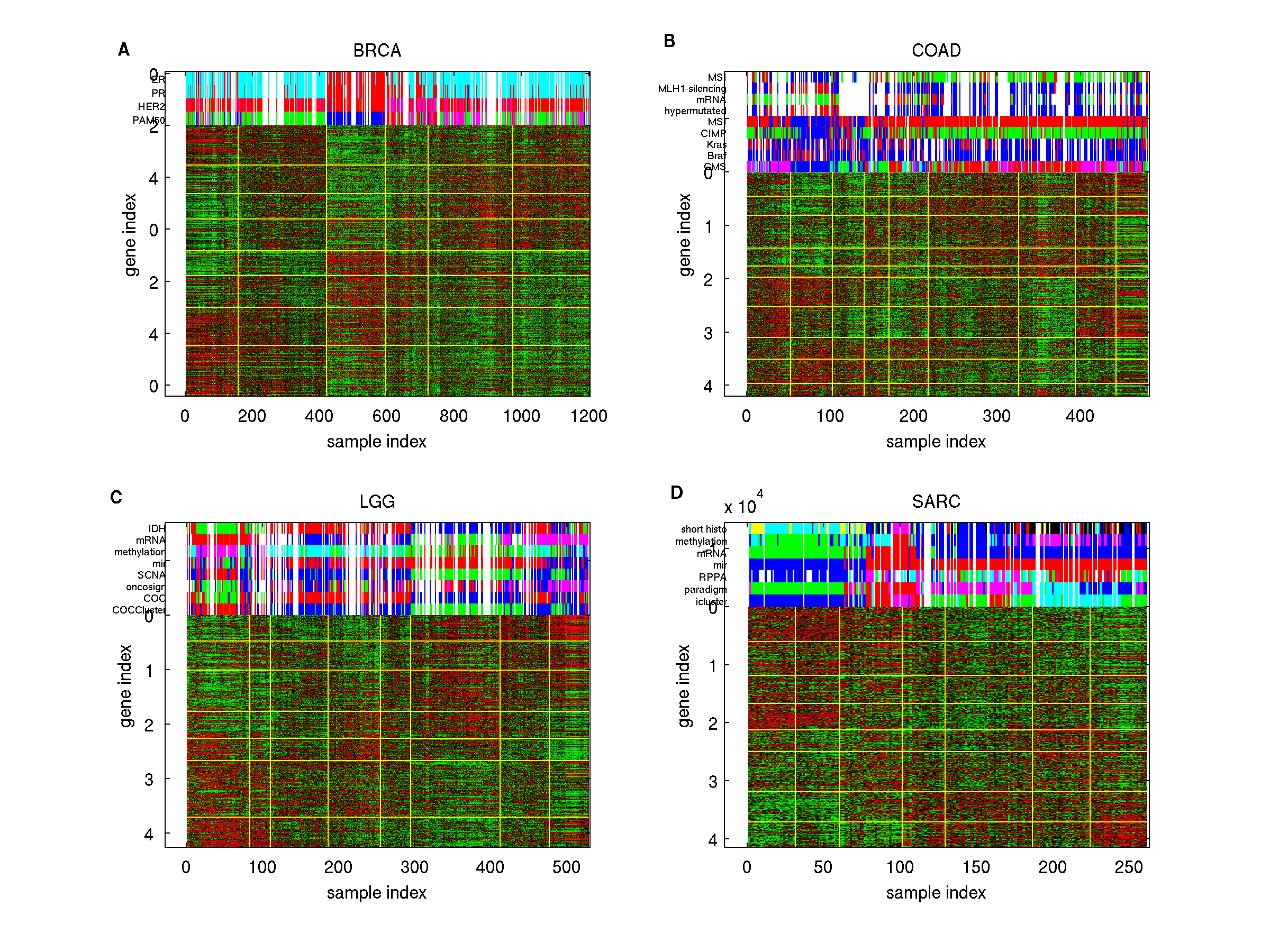

Supplement: S7 Fig — A: BRCA, B: COAD, C: LGG, D: SARC. Dominant feature values of Sample Groups are reported in S5C Table. Feature values of individual samples are reported in S5D Table. (TIF) [file pdig.0000151.s007.tif]

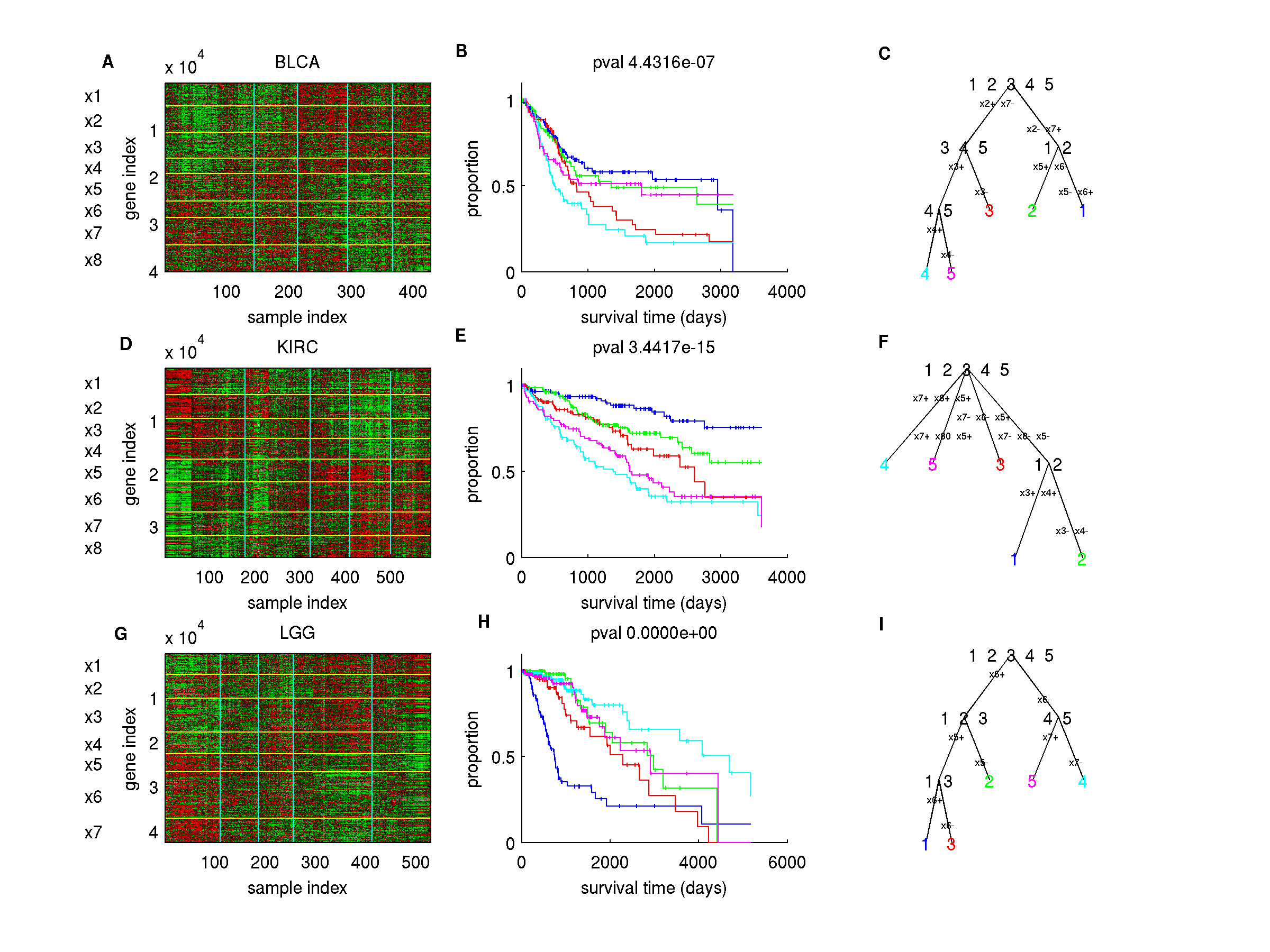

Supplement: S8 Fig — Left panels (A, D, G) display the combinatorial expressions of Super Modules and patient groups. Middle panels (B, E, H) display the Kaplan-Meier curves of patient groups. Right panels (C, F, I) display the decision trees segregating patient groups according to combinatorial expression patterns of Super Modules. Patient groups are separated by cyan vertical lines in their expression data (A, D, G) from left to right, and are annotated by the same colors in their survival curves (B, E, H) and decision trees (C, F, I). (TIF) [file pdig.0000151.s008.tif]

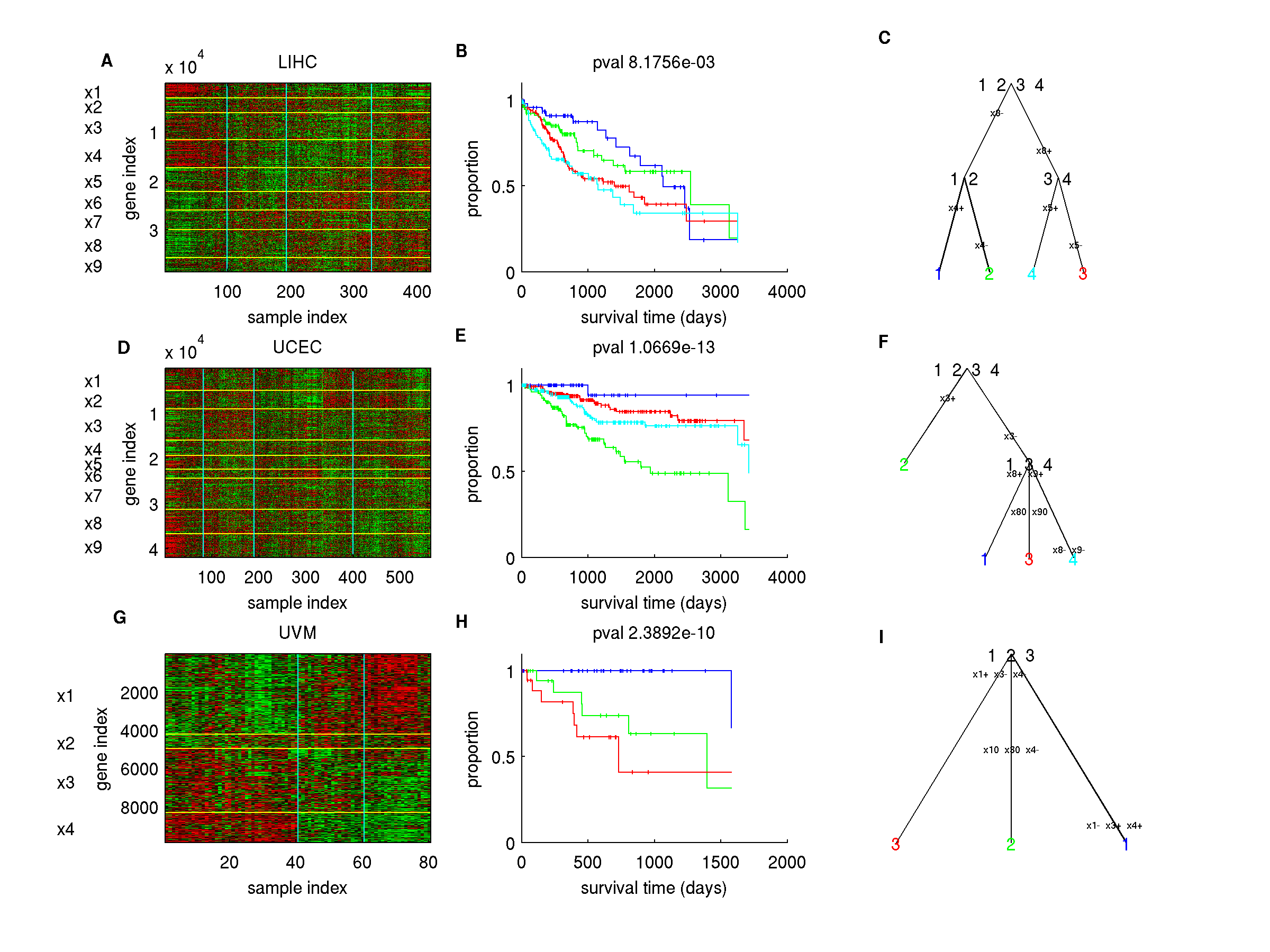

Supplement: S9 Fig — Legend follows S8 Fig. (TIF) [file pdig.0000151.s009.tif]

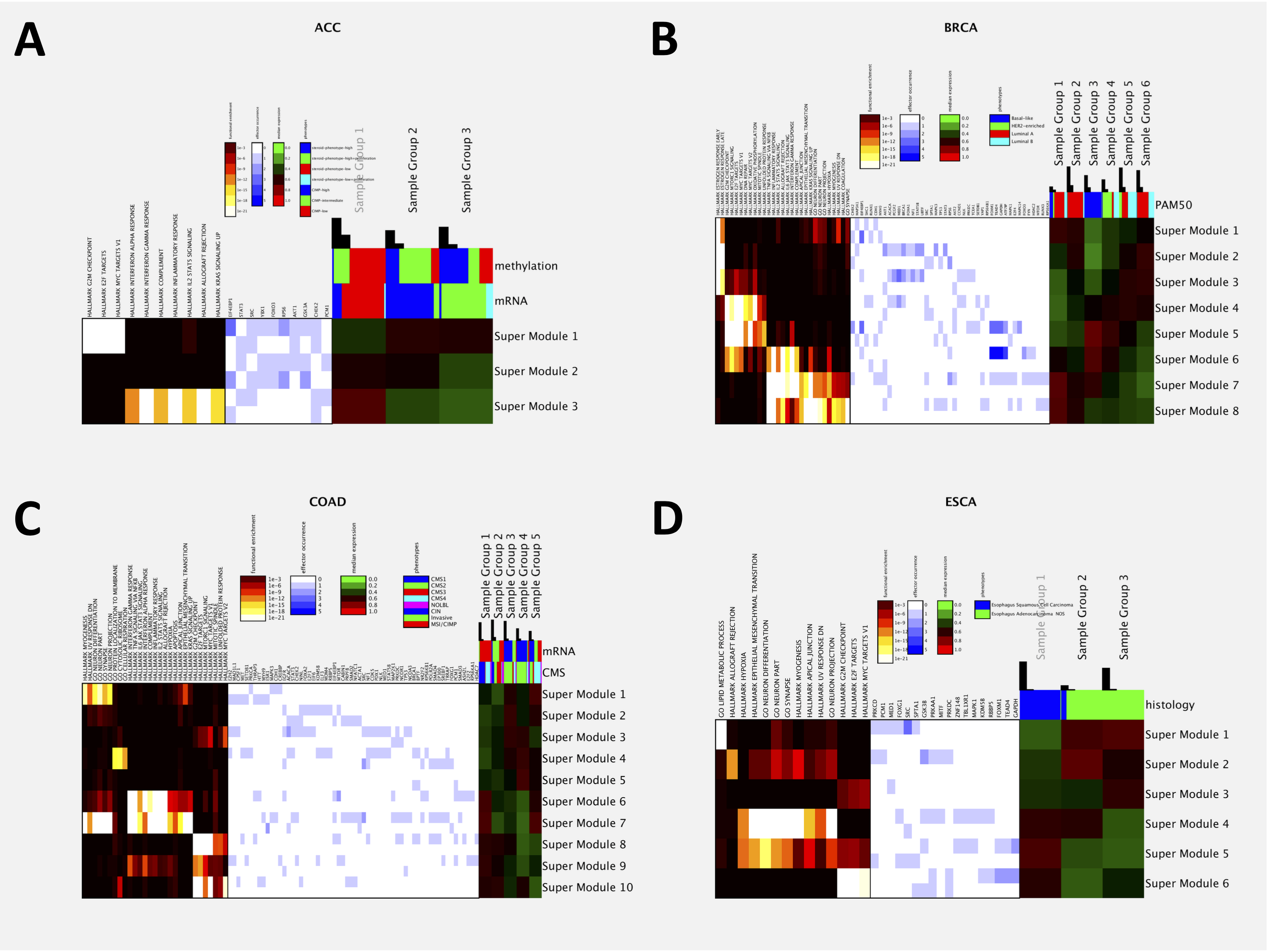

Supplement: S10 Fig — Legend follows Fig 5. (TIFF) [file pdig.0000151.s010.tiff]

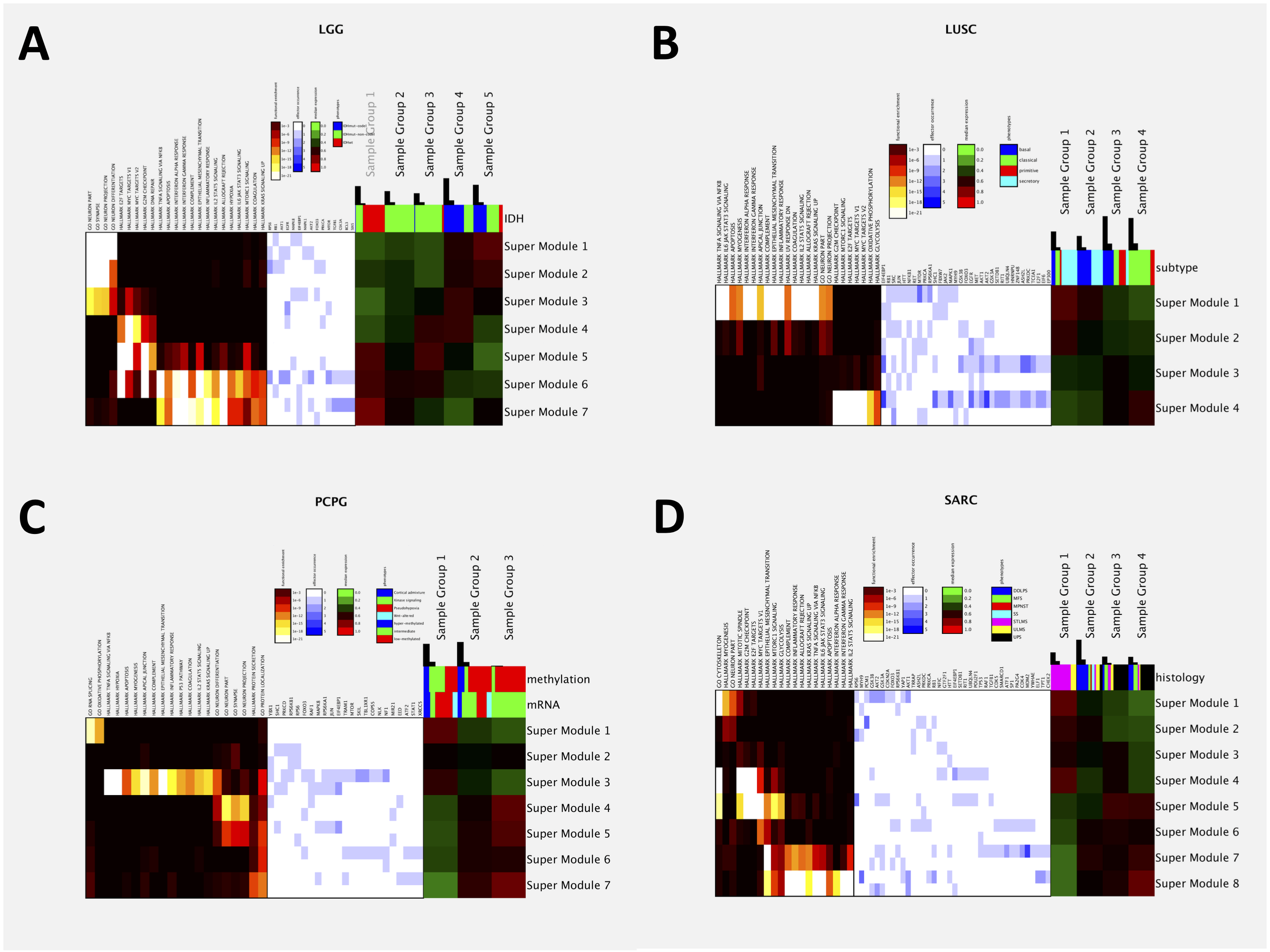

Supplement: S11 Fig — Legend follows Fig 5. (TIFF) [file pdig.0000151.s011.tiff]

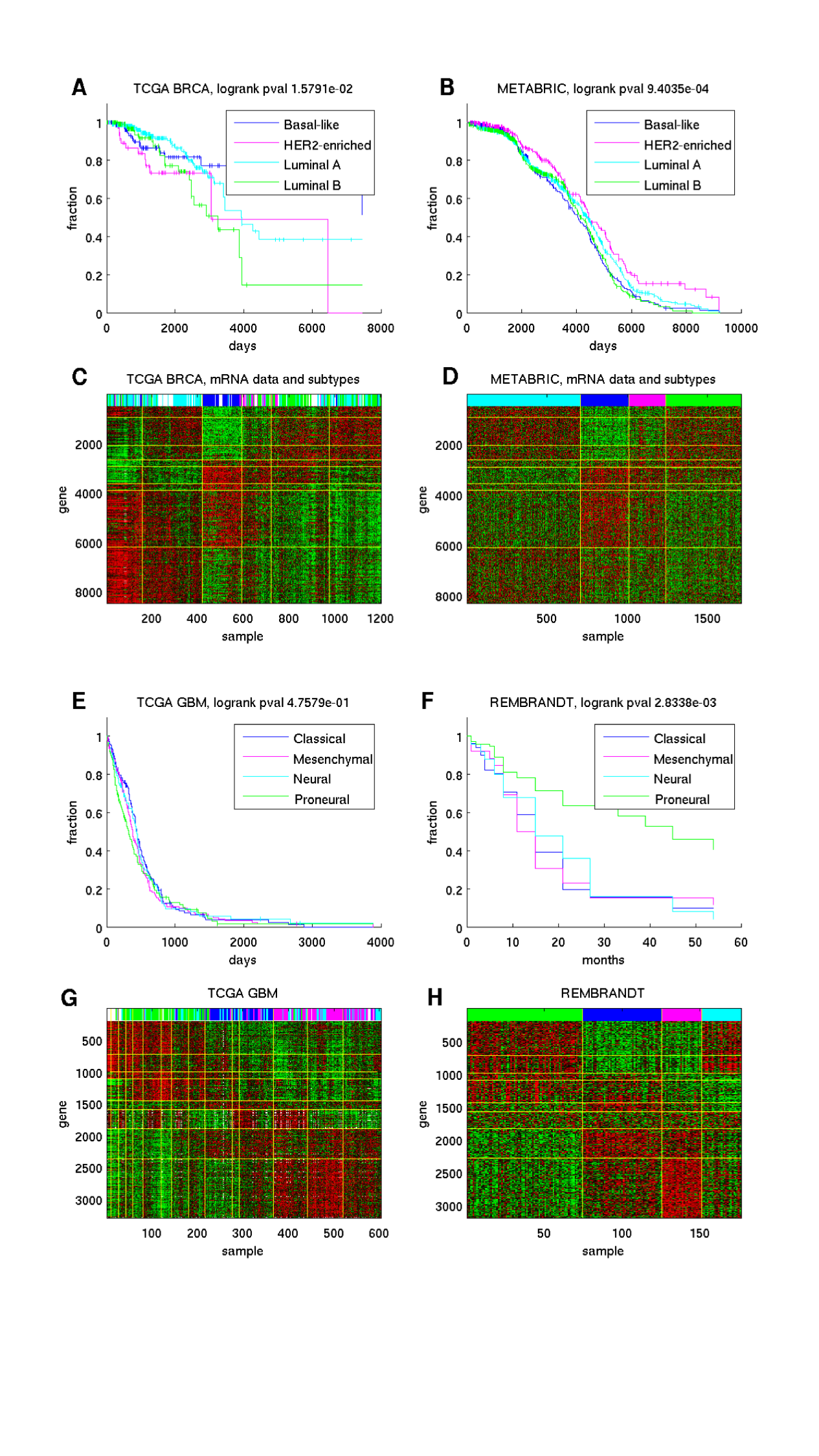

Supplement: S12 Fig — The Kaplan-Meier curves of Sample Groups and combinatorial expressions of Super Modules and Sample Groups of TCGA BRCA (A and C), METABRIC (B and D), TCGA GBM (E and G), and REMBRANDT (F and H) data. (TIFF) [file pdig.0000151.s012.tiff]

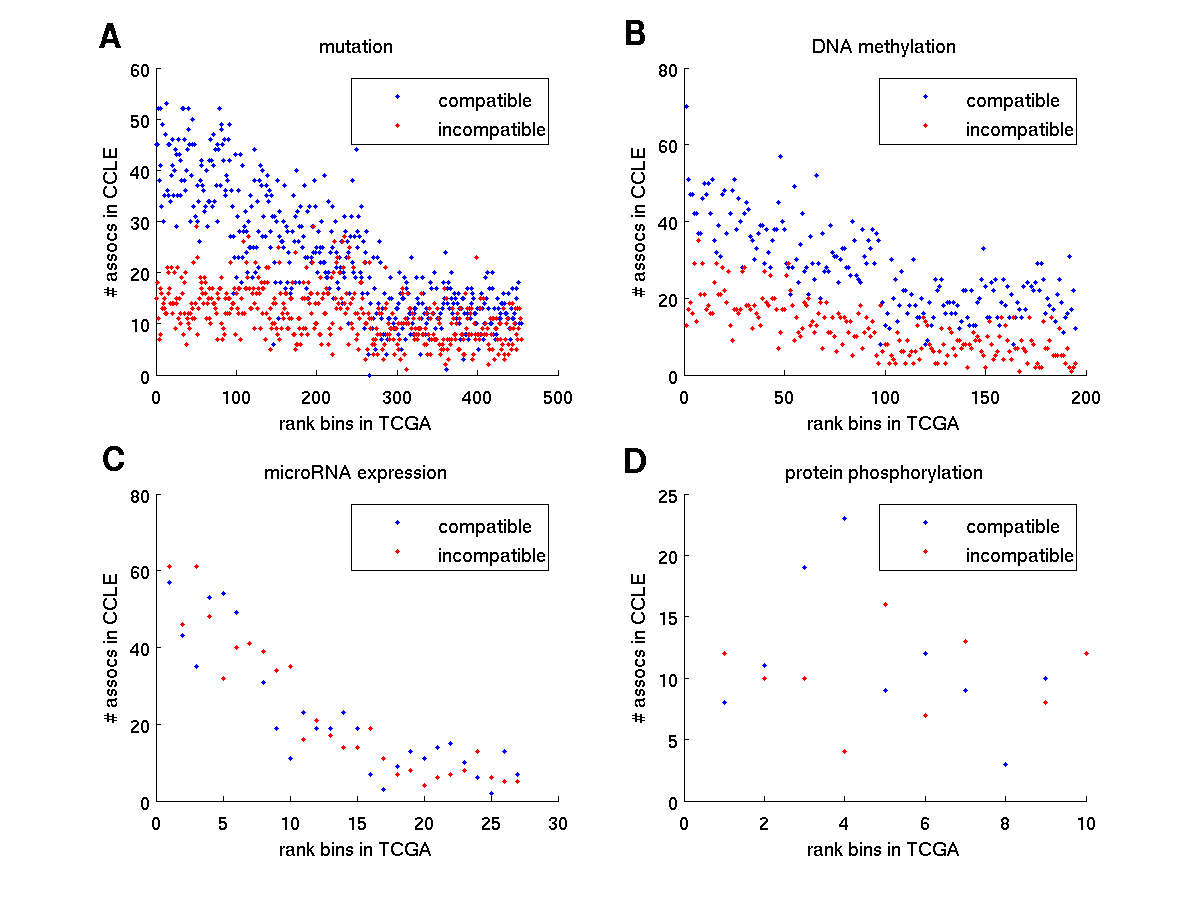

Supplement: S13 Fig — Four panels report the analysis outcomes of four types of effectors: A: mutations, B: DNA methylations, C: microRNA expressions, D: protein phosphorylations. Legend follows Fig 9A. (TIF) [file pdig.0000151.s013.tif]

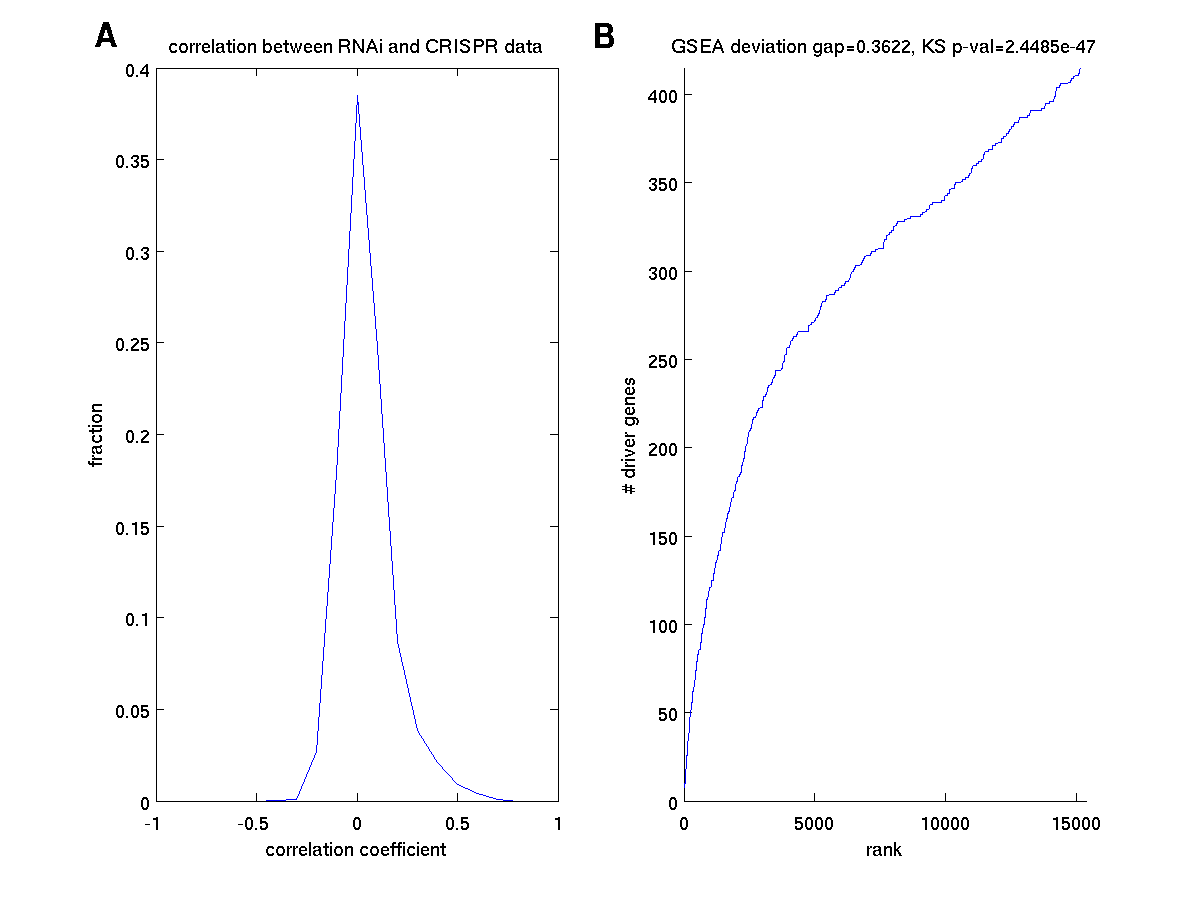

Supplement: S14 Fig — A: The distributions of correlation coefficients between gene dependency data by RNAi and CRISPR perturbations on the same genes. B: The enrichment outcomes of cancer driver genes in the top-ranking perturbed genes in terms of the correlation coefficients between RNAi and CRISPR perturbation data. The GSEA gap and its Kolmogorov-Smirnov p-value are reported. (TIF) [file pdig.0000151.s014.tif]

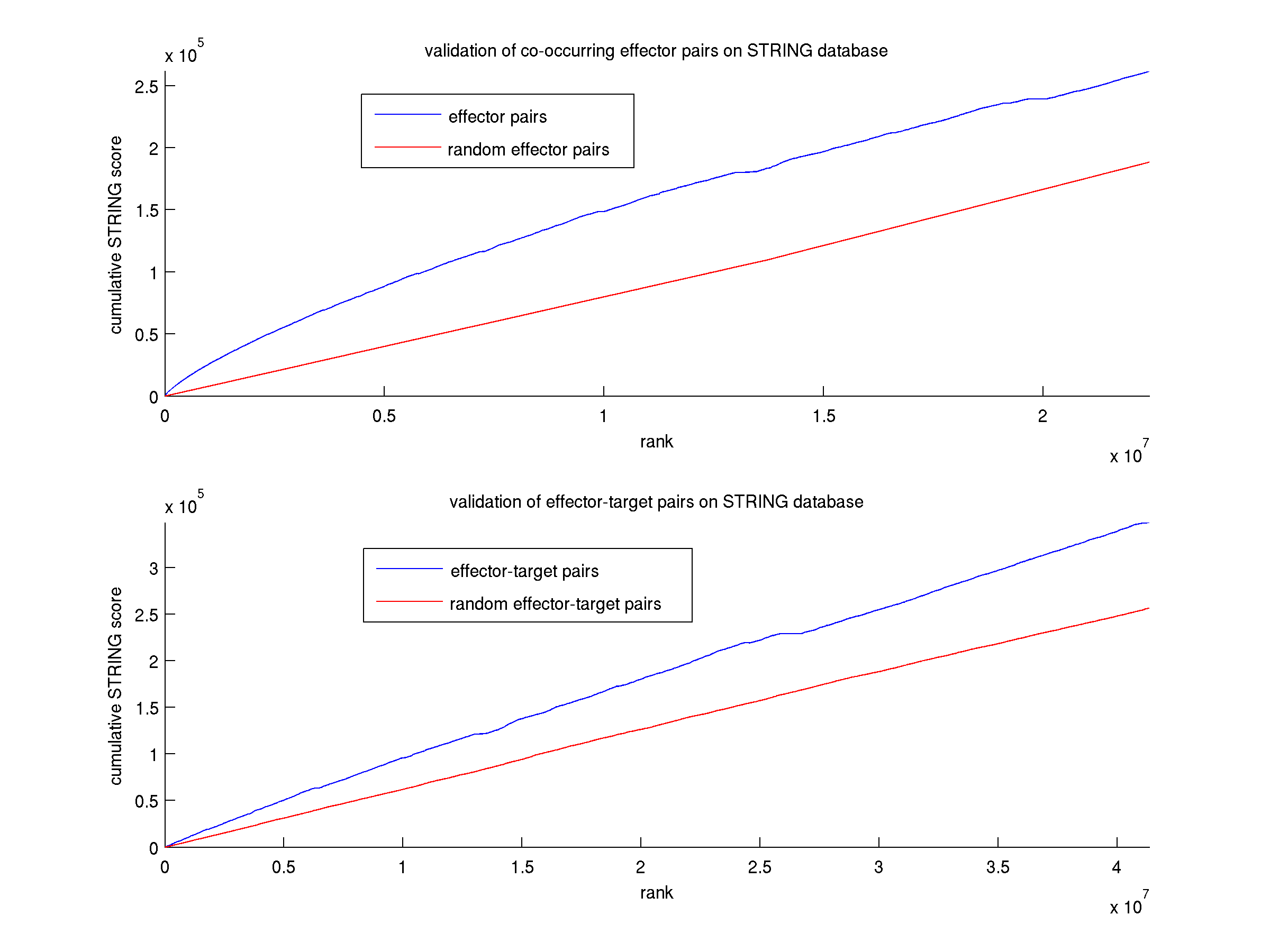

Supplement: S15 Fig — A: Validation of co-occurring effector pairs. Effector pairs are sorted by their co-occurring frequencies in Super Modules, and their cumulative STRING scores are calculated. The ranks of the sorted pairs (x axis) and their cumulative STRING scores (y axis) are displayed. B: Validation of co-occurring effector-target pairs. Legend follows panel A. (TIF) [file pdig.0000151.s015.tif]

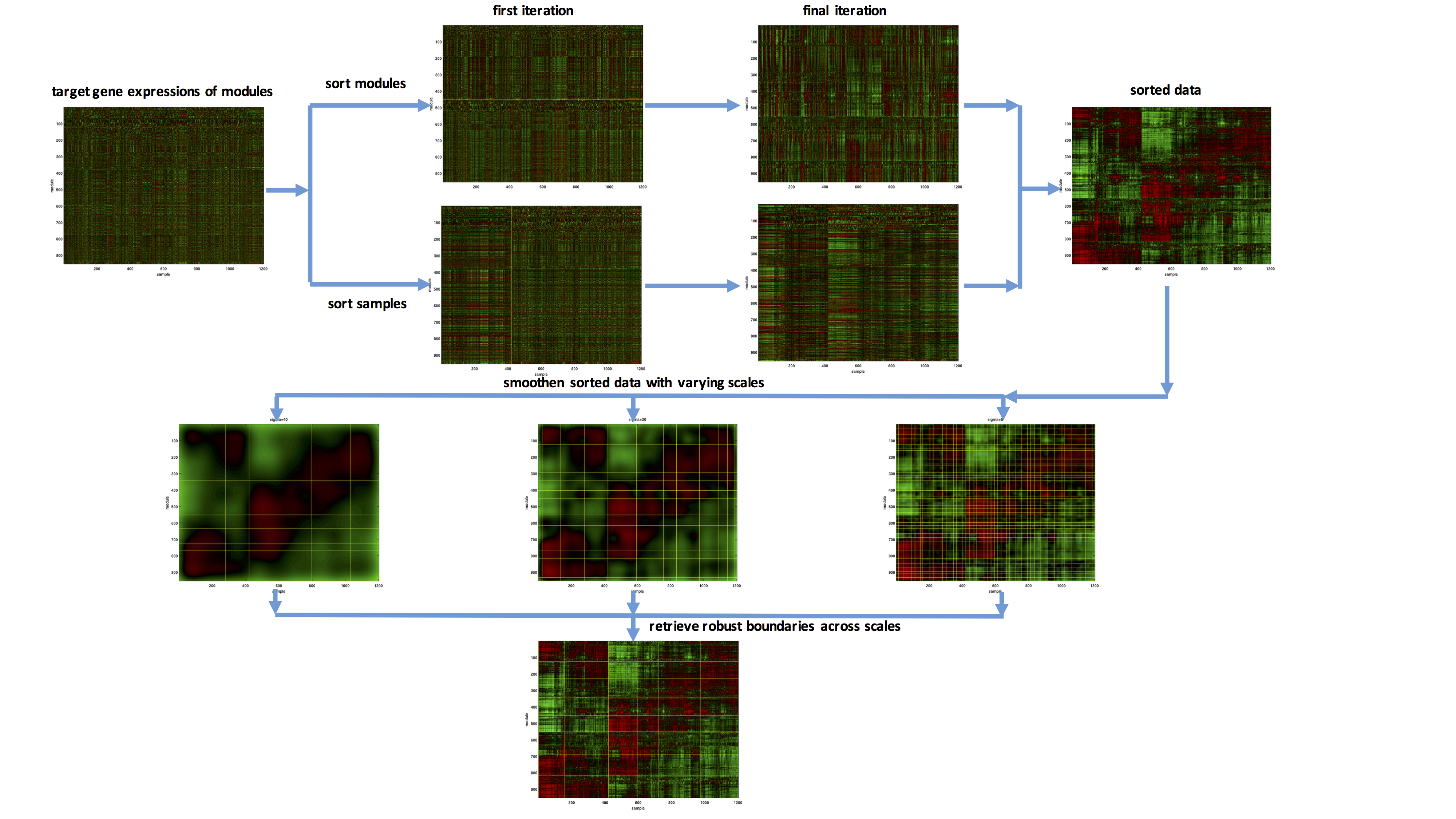

Supplement: S16 Fig — The algorithm comprises three phases. In phase 1, Modules and Samples are sorted by recursively incurring spectral clustering to the rows (Modules) and columns (Samples) of the data separately. In each iteration, subunits (Modules or Samples) are partitioned into two groups. The outputs of phase 1 are the expression data with sorted Modules and Samples. In phase 2, the sorted data are smoothened by convolving with Gaussian kernels with varying scales (standard deviations). Boundaries of each smoothened data are detected. In phase 3, boundaries which are robust against varying scales are selected. The Super Modules and Sample Groups are demarcated by the selected boundaries. (TIFF) [file pdig.0000151.s016.tiff]
