# Supplementary material for: An integrated analysis of the cancer genome atlas data discovers a hierarchical association structure across thirty three cancer types
Source: PLOS Digit Health. 2022 Dec 20;1(12):e0000151. doi: 10.1371/journal.pdig.0000151 (PMC9931374; doi:10.1371/journal.pdig.0000151)
Supplement: S1 Text — (PDF) [file pdig.0000151.s030.pdf]

An integrated analysis of the cancer genome atlas data discovers a hierarchical association structure across thirty three cancer types

Khong-Loon Tiong, Nardnisa Sintupisut, Min-Chin Lin, Chih-Hung Cheng, Andrew Woolston, Chih-Hsu Lin, Mirrian Ho, Yu-Wei Lin, Sridevi Padakanti, Chen-Hsiang Yeang

Supplementary Text S1 provides a detailed description of data processing and analysis methods and some analysis results in the paper.

Table of content

1. Collecting and processing data

   1.1 TCGA data

   1.2 A unified network of molecular interactions

   1.3 External datasets for validation

      1.3.1 MSigDB gene sets

      1.3.2 METABRIC data

      1.3.3 REMBRANDT data

      1.3.4 GEO transcriptome datasets

      1.3.5 CCLE and Achilles data

      1.3.6 Illumina Bodymap data

      1.3.7 Roadmap Epigenomic data

   1.4 Data normalization

1.5 CNV data processing
1.5.1 CNV data normalization
1.5.2 Generating segment CNV data
1.6 Dimension reduction of molecular alteration data
1.6.1 Clustering DNA methylation and microRNA expression data
1.6.2 Trimming molecular alteration data
1.7 Calculating shortest path distances between candidate effectors and targets in the unified network
1.8 Processing external data
2. Inferring IHAS from TCGA data
2.1 Association Models
2.1.1 An exponential family model
2.1.2 Selecting candidate effectors according to pairwise associations
2.1.3 Statistical model selection
2.1.4 Prioritizing candidate effectors using biological knowledge
2.1.5 Summary of constructing Association Models
2.2 Association Modules
2.3 Super Modules and Sample Groups
2.4 Super Module Groups and Gene Groups
2.5 Meta Gene Groups
3. Characterizing functions of IHAS
3.1 Evaluating functional enrichment of Super Modules and Gene Groups
3.2 Inferring Recurrent Effectors of Super Module Groups
3.3 Building the Artery Networks spanned by explanatory paths for associations
3.3.1 Computing edge weights in terms of explanatory paths for associations

3.3.2 Generating the Artery Networks

3.3.3 Generating the Consensus Artery Network from multiple cancer types

4. Relating IHAS with clinical phenotypes

4.1 Aligning Sample Groups with clinical features within cancer types

4.2 Prognostic analysis of Sample Groups within cancer types

4.2.1 Computing Cox regression coefficients and log rank p-values of Kaplan-Meier curves

4.2.2 Assessing statistical significance of prognostic associations for IHAS subunits

4.2.3 Assessing statistical significance of survival function difference between subpopulations differentiated by target gene expressions

4.2.4 Constructing decision trees relating combinatorial expression patterns of Sample Groups with their survival times

4.3 Generating Pan-cancer Sample Groups

4.4 Aligning Pan-cancer Sample Groups with pan-cancer phenotypes

4.5 Integrating effector and target information within and across cancer types

5. Validating IHAS on external datasets

5.1 Juxtaposition of cancer subtypes and combinatorial expression patterns of Super Modules

5.2 Evaluating expression coherence of Super Modules and Gene Groups

5.3 Associations between association structure and survival outcomes

5.4 Aligning the combinatorial expression patterns in the GEO datasets

5.5 Assessing preservation of CNV-mRNA associations

5.6 Assessing preservation of other types of effector-target associations

5.7 Relating Gene Group expressions and drug response data

5.8 Relating Gene Group expressions and gene dependencies

5.9 Verifying the impacts of selected hub effectors in gene dependency data

5.10 Relating IHAS from TCGA and transcriptomic and epigenomic data in normal tissues

6. Comparing IHAS with other multi-omics integration studies and databases

6.1 Comparing IHAS with other multi-omics integration studies

6.2 Verifying IHAS results on the STRING database

1 Collecting and processing data

1.1 TCGA data

We downloaded the level-2 and level-3 data from the original TCGA data portal website (https://tcga-data.nci.nih.gov/tcga/, now moved to https://portal.gdc.cancer.gov/). The following 8 types of data are included: (1) mRNA expression data generated by RNA HiSeq sequencing, Affymetrix or Agilent microarrays, (2) probe-level CNV data generated by Affymetrix Human SNP 6.0 arrays, (3) somatic mutation data derived from exome sequencing and reported in mutation annotation format (MAF), (4) microRNA expressions generated by Illumina RNA HiSeq or GA sequencing, (5) DNA methylation data generated by Illumina 450K array, (6) SNP data generated by Affymetrix Human SNP 6.0 arrays, (7) expression and phosphorylation data of about 200 proteins/amino acid residues generated by reverse phase protein arrays (RPPA), (8) clinical and phenotypical data of patients such as their ages, genders, dates of diagnosis and death, histological and molecular subtypes, and

received treatments. Over the span of 14 years (2005-2018) the TCGA Consortium generated the data of 33 cancer types covering 23359 samples and summarized in Supplementary Table S1A. The data of those cancer types were downloaded at several time points following the release of the data belonging to certain types of cancers. The files in the TCGA data portal are hierarchically organized by first cancer types, second data types (platforms), and third sample IDs. We concatenated the files of a unique cancer type and data type combination into one big table, where each row indicates a gene or probe and each column indicates a sample.

1.2 A unified network of molecular interactions

We generated a large network database regarding human molecular interactions by incorporating multiple sources of biological pathways and networks: (1) Pathway Commons (Cerami *et al*., 2011) is a meta-database containing multiple commonly used databases of molecular interactions such as Reactome, KEGG, BioCyc, DIP, miRTarBase, and others. We downloaded an early version (version 4) in 2012, (2) TRANSFAC (Matys *et al*., 2006) is a manually curated database of eukaryotic transcription factors, their genomic binding sites and DNA binding profiles. We downloaded a proprietary version 2009.1 of human transcription factors and their target genes, (3) miRBase (Kozomara and Griffiths-Jones, 2011, version 19) is a searchable database of published miRNA sequences and annotation. MiRTarBase (Hsu *et al*., 2012, version 4.5) is a database of target genes of microRNAs. We collected the published targets of the microRNAs in 2012, (4) ENCODE (The Encyclopedia of DNA Elements, The ENCOCE Project Consortium, 2012) builds a comprehensive parts list of functional elements of human cells and generates many datasets of functional genomic assays. We collected the ENCODE data of ChIP-Seq experiments in human cell lines in

2012 (version 2) and converted them into the list of protein-DNA interactions. For each type of data, we mapped the entries to gene names according to the NCBI database, collapsed the synonyms of the same genes and redundant entries, and pruned the entries without corresponding gene names.

The unified network compiled from the aforementioned sources is a hyper-graph consisting of molecules (nodes) and interactions (hyper-edges). Beyond the non-trivial data from these sources, we also augmented several types of dummy molecules and interactions in order to facilitate enumerating valid paths. Each protein coding gene comprises three molecules of DNA, mRNA and protein, and an microRNA comprises two molecules of DNA and microRNA. Hence dummy molecules (DNAs, mRNAs, microRNAs, proteins) of genes are added if they are absent in the unified network. Furthermore, the dummy interactions (DNA,mRNA), (mRNA,protein), (DNA,microRNA) of the same genes/microRNAs indicate information flows of transcription and translation, thus are added to the unified network. The augmented network was further consolidated according to two criteria: (1) molecules with the same identities but different names (e.g., synonyms of the same gene, or upper and lower case expressions of the same name) were collapsed, (2) molecules not involved in regulatory relations (e.g., an isolated protein complex connected to only its component proteins, or a gene not traversed by any paths of protein-DNA and protein-protein interactions) were discarded. The resulting unified network consists of 90122 molecules and 1068050 interactions. There are 13 types of molecules – DNA regions, RNA regions, Proteins, RNAs, Complexes, Gene classes, Mir classes, Dummy DNAs, Dummy mRNAs, Dummy proteins, Dummy mirs, Small molecules, and Physical entities. Complexes refer to large molecules consisting of smaller subunits of proteins or RNAs. Gene (mir) classes refer to a collection of genes (mirs) that may carry similar functions and do not physically bind together. Small

molecules refer to mostly metabolites such as glucoses and phosphates. Dummy DNAs, mRNAs, proteins and mirs were created by us to build the central dogma links of the same genes (mirs) such as DNA-mRNA-protein and DNA-mir. Interactions are hyper-edges constituting one or multiple molecules. There are 20 types of interactions – Biochemical reactions, Catalysis, Complex assembly, Control, Conversion, Degradation, Modulation, Molecular interactions, Template reactions, Transport, Transport with biochemical reaction, Dummy2member, Genemirclass2dummy, DNARNA, RNAprotein, Complex2member, ProteinDNA, MirRNA. Most of those molecule and interaction types are self-explanatory and reported in the Pathway Commons database. For instance, Biochemical reactions consist of small molecules as substrates and products and proteins or protein complexes as enzymes. Complex assembly interactions consist of subunits (proteins or protein subcomplexes) as inputs and one protein complex as an output. In contrast, Complex2Member interactions consist of one complex as an input and multiple member proteins or subcomplexes as outputs. Molecular interactions are bipartite, symmetric relations specifying the bindings of two molecules. DNARNA and RNAProtein interactions specify the central dogma information flows of the same genes. ProteinDNA and MirRNA interactions specify the regulatory relations of transcription factors or microRNAs to their targets.

1.3 External datasets for validation

1.3.1 MSigDB gene sets

We downloaded 14545 gene sets from the MSigDB database (https://www.gsea-msigdb.org/gsea/msigdb). They comprise members of functional categories from several

large-scale databases including GO, KEGG, REACTOME, PID pathways, BIOCARTA, HALLMARK, and differentially expressed genes in many datasets.

1.3.2 METABRIC data

We acquired an approval from the METABRIC data access committee and downloaded the METABRIC data of 1981 breast cancer patients from (Curtis *et al*., 2012). They include the mRNA expression data of 49576 probes, CNV data of 17013 loci, and clinical data of patients such as survival times, PAM50 subtypes, tumor histology, and many others.

1.3.3 REMBRANDT data

We downloaded the REMBRANDT data of 176 brain tumor patients from (Madhavan *et al*., 2009). They include the mRNA expression data of 21029 genes, CNV data of 8066 loci, and clinical data of patients such as survival times, tumor histology, and mRNA subtypes.

1.3.4 GEO transcriptome datasets

We collected 388 transcriptomic datasets pertaining to the 33 cancer types from the GEO database during 2015-2016. To find out those datasets, we searched the GEO database with the keywords of each cancer type and manually picked the ones which contained ≥ 10 cancer samples according to the data descriptions. Those datasets are filtered out if they satisfy at least one of the following conditions.

1. They contain too few (≤ 30) samples.
2. They have too few intersected genes with all Super Module targets.

3. Their expressions have small variations across samples.
4. They are subsets of larger datasets that appear on the list.
5. They contain only cell lines or normal tissues but no tumors.

294 datasets pass those filtering criteria. Among them 54 have survival information. Supplementary Table S9 reports the characteristics of the 294 selected datasets.

1.3.5 CCLE and Achilles data

The Cancer Cell Line Encyclopedia (CCLE) is a comprehensive, integrative database of multi-omic data over 1046 cancer cell lines (Barretina *et al.*, 2012). It consists of the data of mRNA expressions, CNV, SNP, mutations, DNA methylations, microRNA expressions, protein expressions and phosphorylations, and growth responses to 26 drugs. Another large-scale project, the Achilles project (Tsherniak *et al.*, 2017), generated the gene dependency data of selected CCLE cancer cell lines. Selected genes were knocked down by RNAi or CRISPR-Cas9 on those cancer cell lines, and their growth responses were reported. We downloaded the CCLE data in 2017 and 2018 and found the two versions of data were generally compatible but not identical. We selected or joined different types of data with distinct criteria.

The old version of mRNA expression data was measured by microarrays and provided as gct and res file formats. The new version of mRNA expression data was probed by RNA-Seq and provided as the RPKM format. We calculated the distribution of correlation coefficients between the old and new mRNA expression data of the same genes and found they were

highly or moderately correlated (Figure X1). We chose the old microarray data since they covered more cancer cell lines than the new RNA-Seq data.

Figure X1: The sorted correlation coefficients between old and new CCLE mRNA expression data of the same genes.

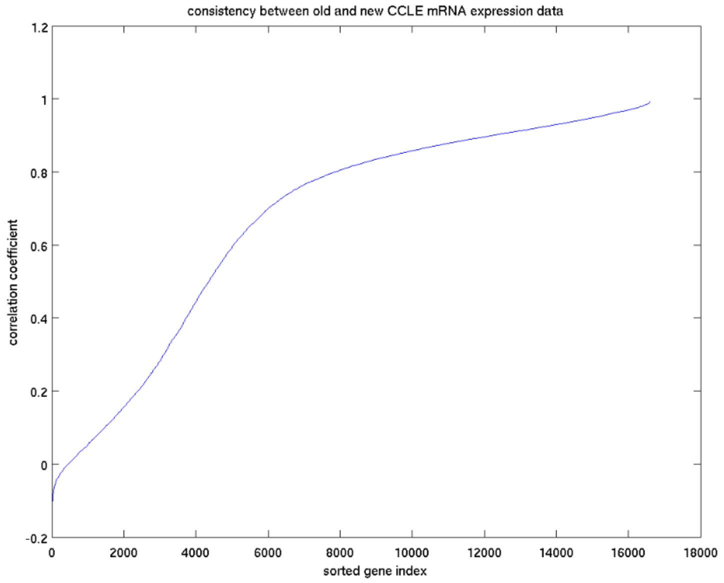

The CNV data appeared in the old version only. The data at probe, gene and segment levels are all provided in CCLE. Therefore, we downloaded the segment-level CNV data without undergoing the process of partitioning probe-level data into segment-level data. DNA methylation, microRNA expression and protein expression/phosphorylation data are provided only in the new version. Thus we used the new version of data without having to make a choice.

The old version of mutation data is measured by three DNA sequencing methods: whole exome sequencing (WES), oncomap, and hybrid capture sequencing. The new version of mutation data is merged mutation calls in the protein coding regions with germline mutations removed. The oncomap sequencing covers only a small number of genes. Thus we compared the WES data of the old version and the mutation calls of the new version (Figure X2). Unlike mRNA expression data, the old and new mutation data are poorly consistent. We decided to include the new mutation data for they covered more genes. This choice is justified since the recurrent mutations in TCGA are verified in CCLE mutation data (Supplementary Figure S8A).

The pharmacological profile data consists of the drug response information by treating CCLE cell lines with 26 chemical compounds. Various types of information are reported, such as doses, mean and standard deviation of activities, EC_{50} and IC_{50}. We extracted only the IC_{50} values and generated a table where rows were compounds, columns were cell lines, and entries were IC_{50} values. We filtered out 5 compounds with very sparse data and considered the data of 21 drugs. Table X1 lists those 21 compounds and their characteristics.

The Achilles project reports the growth responses of selected CCLE cell lines by perturbing many genes with RNAi and CRISPR technologies. 15366 genes are perturbed with both technologies. We calculated the correlation coefficients between the RNAi and CRISPR dependency data of those genes (Figure X3, left panel) and found a slight tendency of positive correlation: mean and median values were 0.0463 and 0.0264 respectively. However, the genes with strongly correlated dependency data are highly enriched with cancer-related driver genes. We extracted 459 cancer driver genes from the Intogen database (Martinez-Jimenez *et al.*, 2020) and found they were highly concentrated among the top-

ranking genes in terms of RNAi-CRISPR correlations (Figure X3, right panel). Therefore, we selected the top 2000 genes whose dependency data were robust against the perturbation technologies.

Figure X2: The sorted consistency between old and new CCLE mutation data of the same genes. Consistency of a gene is defined as the ratio between the number of samples carrying mutations in both datasets and the number of samples carrying mutations in at least one dataset.

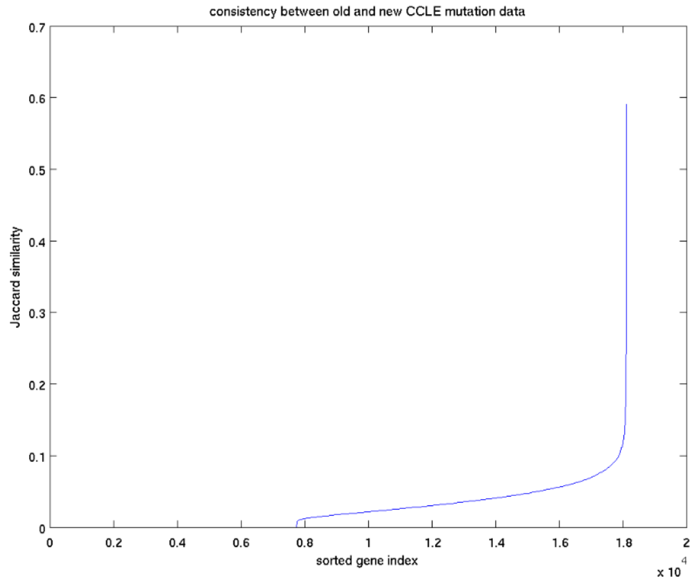

Table X1: Summary of 21 compounds in CCLE drug response data

compound	Targets	mechanism of action	class
erlotnib	EGFR	EGFR inhibitor	kinase inhibitor
lapatinib	EGFR, HER2	EGFR & HER2 inhibitor	kinase inhibitor
PHA-665752	c-MET	c-MET inhibitor	kinase inhibitor
TAE684	ALK	ALK inhibitor	kinase inhibitor
Nilotinib	Abl/Bcr-Abl	Abl inhibitor	kinase inhibitor
AZD0530	Src, Abl/Bcr-Abl, EGFR	Src and Abl inhibitor	kinase inhibitor
sorafenib	Flt3, C-KIT, PDGFRβ, RET, Raf kinase B, Raf kinase C, VEGFR-1, KDR, FLT4	multi-kinase inhibitor	kinase inhibitor
TKI258	EGFR, FGFR1, PDGFRβ, VEGFR-1, KDR	multi-kinase inhibitor	kinase inhibitor
PD-0332991	CDK4/6	CDK4/6 inhibitor	kinase inhibitor
AEW541	IGF-1R	IGF-1R inhibitor	kinase inhibitor
RAF265	Raf kinase B, KDR	Raf kinase B and KDR inhibitor	kinase inhibitor
PLX4720	RAF	Raf kinase B inhibitor	kinase inhibitor
PD-0325901	MEK	MEK1 and MEK2 inhibitor	kinase inhibitor
AZD6244	MEK	MEK1 and MEK2 inhibitor	kinase inhibitor
nutlin-3	MDM2	inhibitor of Apoptosis Proteins (IAP) inhibitor	other targeted therapies

LBW242	IAP	inhibitor of apoptosis proteins (IAP) inhibitor	other targeted therapies
17-AAG	HSP90	heat shock protein 90 (HSP90) inhibitor	other targeted therapies
L-685458	γ-secretase	γ-secretase inhibitor	other targeted therapies
paclitaxel	β-tubulin	microtubule-stablizing agents	cytotoxic
irinotecan	topoisomerase I	DNA topoisomerase I inhibitor	cytotoxic
topotecan	topoisomerase I	DNA topoisomerase I inhibitor	cytotoxic

Figure X3: Left – Distribution of correlation coefficients between RNAi and CRISPR data of the same genes in Achilles. Right – Numbers of reported driver genes among top-ranking genes sorted by correlation coefficients.

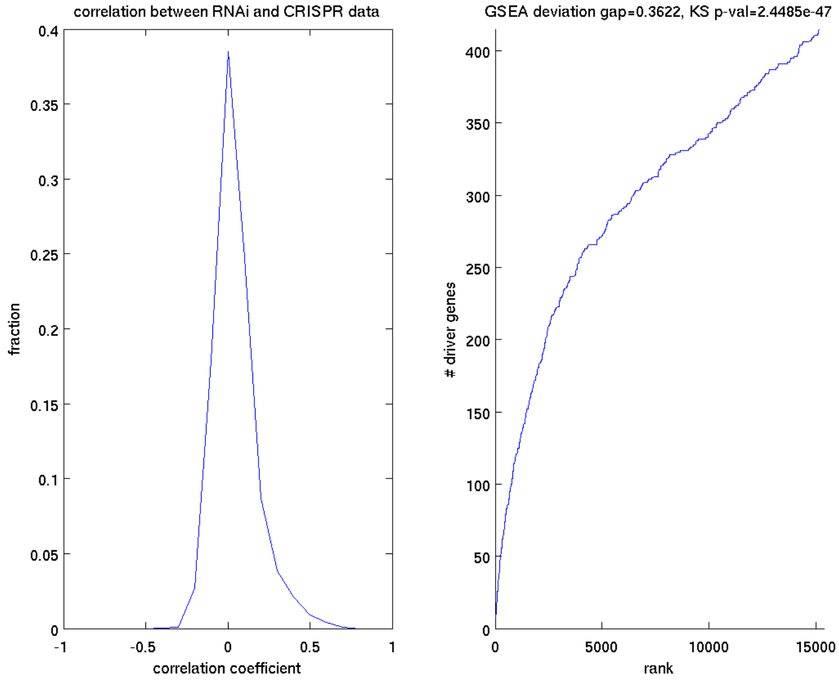

1.3.6 Illumina Bodymap data

The Illumina BodyMap (ArrayExpress ID: E-MTAB-513) provides transcriptomic (RNA-Seq) data of normal tissues. It has a relatively small size with 27496 genes and 16 samples. The following normal tissues are covered: adipose tissue, adrenal gland, brain, breast, colon, heart, kidney, leukocyte, liver, lung, lymph node, ovary, prostate gland, skeletal muscle, testis, thyroid gland.

1.3.7 Roadmap Epigenomic data

The Roadmap Epigenomic data provides measurements of dozens of epigenomic markers over many normal human tissues (Roadmap Epigenomics Consortium, 2015). The probed epigenomic markers include nucleosome positions, histone methylations, histone acetylations, and mRNA expressions. The covered samples include various human cell lines derived from stem or iPS cells, stem cells, blood and immune cells, skin cells, neuron cells, cells from various organs, and cells from fetal tissues. The original Roadmap data has a complicated structure which is difficult to directly compare with the integrated hierarchical association structures inferred from TCGA. Each type of epigenomic marker is probed by a genome-wide assay and reported by genome-wide peak callings from the measurements. For instance, a histone methylation mark (such as H3K4me3) is typically measured by genome-wide ChIP-Seq assays, and the peak calls from the ChIP-Seq measurements all over the genomic regions are reported. Different combinations of those epigenomic markers imply distinct epigenomic/regulatory states. The Roadmap team deciphered the measured epigenomic markers into 25 distinct epigenomic states, partitioned the genome of a sample

into small segments, and reported the predicted epigenomic states of the segments. Table X2 lists the 25 predicted epigenomic states of the Roadmap project. We downloaded the predicted epigenomic states of chromosomal segments of the Roadmap data.

Table X2: 25 predicted epigenomic states of the Roadmap data

index	name	annotation
1	TssA	transcription start site
2	PromU	upstream promoter
3	PromD1	downstream promoter 1
4	PromD2	downstream promoter 2
5	Tx5’	5’ site of transcription
6	Tx	strong transcription
7	Tx3’	3’ site of transcription
8	TxWk	weak transcription
9	TxReg	transcribed & regulatory
10	TxEnh5’	transcribed 5’ preferential & enhancer
11	TxEnh3’	transcribed 3’ preferential & enhancer
12	TxEnhW	transcribed and weak enhancer
13	EnhA1	active enhancer 1
14	EnhA2	active enhancer 2
15	EnhAF	active enhancer flank
16	EnhW1	weak enhancer 1
17	EnhW2	weak enhancer 2
18	EnhAc	primary H3K27ac possible enhancer
19	DNase	primary DNase
20	ZNF/Rpts	ZNF genes & repeats
21	Het	heterochromatin

22	PromP	poised promoter
23	PromBiv	bivalent promoter
24	ReprPC	repressed PolyComb
25	Quies	quiescent

1.4 Data normalization

The TCGA data are from heterogeneous sources with distinct properties, value ranges and interpretations. Some possess categorical values (such as SNP genotypes and somatic mutation states), while others possess numerical values (such as mRNA or microRNA expressions). Some possess a wide range of numerical values (such as FPKM values for RNA-Seq data), while others possess a narrow range of numerical values (such as the β values for DNA methylation data). To incorporate them in the same modeling and analysis framework, we converted those diverse types of data into the same format with compatible scales. We treated the true value of a feature in each sample as a random variable with discrete hidden states, and its observed value in a dataset as a noisy measurement outcome. The proper representation of a feature value is a posterior probability vector over the hidden states conditioned on the observed data. Consequently, a data matrix (features $\times$ samples) is converted into a data probability tensor (features $\times$ samples $\times$ hidden states) with values in $[0,1]$.

The hidden states of categorical data types (mutations and SNPs) are automatically defined. For mutation data, a gene possesses three states, indicating whether it undergoes no mutation or silent mutation (state 0), missense point mutations or in-frame insertions/deletions that do not necessarily block mRNA synthesis (state 1), and nonsense point mutations or frame-

shifting insertions/deletions that disrupt mRNA synthesis (state 2). The MAF file consists of multiple rows indicating the mutation records of nucleotides or segments. One gene may possess multiple records if it undergoes mutations at multiple positions. We collapsed those multiple records into a single state by choosing the records of the strongest transcriptional impacts. If nonsense mutations occur in the gene, then assign it to state 2. If nonsense mutations do not occur but missense mutations occur in the gene, then assign it to state 1. If only silent mutation or no mutation records are reported, then assign it state 0. The inferred mutation state is converted into the probability vector without uncertainty: state 0 – $(1,0,0)$, state 1 – $(0,1,0)$, state 2 – $(0,0,1)$. An entry with a missing value is converted to $(0.5,0.25,0.25)$ indicating equal probabilities of no mutations and mutations and equal probabilities of missense and nonsense mutations.

An entry of SNP arrays possesses trinary values $(0,1,2)$ indicating the number of minor alleles (or homozygote major alleles, heterozygote alleles, and homozygote minor alleles) at the corresponding locus. For instance, if A and G are the major and minor alleles of the locus, then the haplotypes AA, AG, GA and GG correspond to the values 0, 1, 1 and 2. It is already a discrete state and can be converted into the probability vectors $(1,0,0), (0,1,0), (0,0,1)$ respectively. A missing entry was converted into the vector $(\frac{1}{3}, \frac{1}{3}, \frac{1}{3})$ of equal probabilities over hidden states.

All the remaining data types (mRNA expressions, CNV, microRNA expressions, DNA methylations, protein expressions and phosphorylations) possess numerical values. We postulated that each entry had three hidden states. For expressions, the three states correspond to up (+1), down (-1) regulation and no change (0). For CNV, the three states correspond to amplification (+1), deletion (-1) and normal karyotypes (0). For DNA

methylation, the three states correspond to hyper (+1), hypo (-1) methylation and normal levels (0). For protein phosphorylation, the three states correspond to high (+1), moderate (0) and low (-1) levels of phosphorylation. We converted measurements of continuous random variables (mRNA and microRNA expressions, CNV values, etc) into discrete random variables of three states for the following reasons. First, since the multi-omics data include both discrete (mutations, SNPs) and continuous (other data types) measurements, it is easier to formulate models of discrete random variables than hybrid models of both discrete and continuous random variables. Second, models of discrete random variables can better accommodate the combinatorial functions of gene regulation than models of continuous or hybrid random variables. A rare but possible example is the XOR function of binary variables. Suppose either of two transcription factors can activate a target gene, but the presence of both has an antagonistic effect, then the target gene expression Y is the XOR function of the activities of two transcription factors X_1 and X_2: $Y = X_1 \cdot \overline{X_2} + \overline{X_1} \cdot X_2$. This combinatorial function does not have an obvious extension in continuous variables. Third, tristate discretization is a reasonable choice in this work since it is compatible with the nature of most data types: mutations (0: no mutation or synonymous mutation, 1: missense mutation, 2: nonsense mutation), SNPs (0 and 2: homozygote major and minor alleles, 1: heterozygote allele), and CNVs (0: no change, 1: amplification, 2: deletion) all have three states. Other types of data (such as expressions and protein/DNA modifications) do not have strong preference for a particular number of discrete states, yet trinary states are compatible with other variables, yield better resolution than binary states, and still have a manageable number of combinatorial functions to exhaust. Fourth, information about continuous variable measurements will be incorporated in a quantization procedure introduced below. Continuous measurement levels are converted into probabilities of hidden states, hence the arbitrary discretization thresholds are not needed.

We proposed a *probabilistic quantization* procedure to convert an entry value into a trinary probability vector. For each dataset, denote z_{ij} the observed value of probe i in sample j, and x_{ij} its discrete hidden state. The following procedures convert z_{ij} into a probability vector $(P(x_{ij} = -1), P(x_{ij} = 0), P(x_{ij} = 1))$.

Figure X4: Probabilistic quantization algorithm

Input: A continuous omic data matrix $\boldsymbol{Z}$ with rows and columns as probes and samples.

Output: Trinary probability vectors of the hidden states x_{ij} for each entry z_{ij} in $\boldsymbol{Z}$.

Procedures:

1. If z_{ij} has a missing value, then assign an equal probability to each state $\left(P(x_{ij} = -1), P(x_{ij} = 0), P(x_{ij} = 1)\right) = (\frac{1}{3}, \frac{1}{3}, \frac{1}{3})$.
2. Rank-transform z_{ij} into a cumulative distribution function (CDF) value $y_{ij} \in [0,1]$. For the datasets reporting relative values (e.g., Agilent microarrays), rank transformation is applied to the entire matrix. For the datasets reporting absolute values (e.g., Affymetrix microarrays or RNA-Seq data), each feature (probe or gene) is rank-transformed separately. This is because we want to capture the relative variation of feature values across different samples instead of comparing the values of distinct features that might have quite different intrinsic levels. DNA methylation data are scaled in $[0,1]$ thus need not to be rank-transformed.

3. Convert y_{ij} into a probability vector $\left(P(x_{ij} = -1), P(x_{ij} = 0), P(x_{ij} = 1)\right)$ with a parametric quantization function. Intuitively, $P(x_{ij} = 1)$ is positively and monotonically related to y_{ij}, and $P(x_{ij} = -1)$ is negatively and monotonically related to y_{ij}. We chose polynomial functions f_γ and $\bar{f}_\gamma$ as the quantization functions.

$$P(x_{ij} = +1 | y_{ij}, \gamma) = f_\gamma(y_{ij}) \equiv {y_{ij}}^\gamma$$
$$P(x_{ij} = -1 | y_{ij}, \gamma) = \bar{f}_\gamma(y_{ij}) \equiv (1 - y_{ij})^\gamma \quad (1)$$
$$P(x_{ij} = 0 | y_{ij}, \gamma) = 1 - P(x_{ij} = +1 | y_{ij}, \gamma) - P(x_{ij} = -1 | y_{ij}, \gamma)$$

Parameter γ controls the *soft threshold* of assigning x_{ij} to $+1$ or -1. A higher γ lifts the threshold on y_{ij} (and $1 - y_{ij}$) of calling the hidden state x_{ij} to be $+1$ (or -1). Thus a higher γ raises $P(x_{ij} = 0)$ and reduces $P(x_{ij} = \pm 1)$.

4. Quantization results are sensitive to γ values. To reduce the bias induced by a specific quantization function we assigned weights (prior) on f_γ and $\bar{f}_\gamma$ functions and integrated the transformed values over a family of quantization functions. In this work we chose an exponential prior $e^{-(\gamma-1)}$ and restricted $\gamma \in [1, \infty)$. The averaged quantization outputs are:

$$P(x_{ij} = +1 | y_{ij}) = \int_1^\infty e^{-(\gamma-1)} f_\gamma(y_{ij})\, d\gamma = \frac{y_{ij}}{(1 - \log y_{ij})}$$
$$P(x_{ij} = -1 | y_{ij}) = \int_1^\infty e^{-(\gamma-1)} \bar{f}_\gamma(y_{ij})\, d\gamma = \frac{1 - y_{ij}}{(1 - \log(1 - y_{ij}))} \quad (2)$$
$$P(x_{ij} = 0 | y_{ij}) = 1 - P(x_{ij} = +1 | y_{ij}) - P(x_{ij} = -1 | y_{ij})$$

The exponential prior $e^{-(\gamma-1)}$ was chosen for the following reasons. First, large γ values are penalized because they assign the probability mass to $x_{ij} = 0$ for most y_{ij} values. An exponential prior naturally penalizes large γ values. Second, it ensures the existence of the integrals in equation 1. Third, the requirements that $P(x_{ij} = +1 | y_{ij} = +1) = 1$ and $P(x_{ij} = -1 | y_{ij} = 0) = 1$ are satisfied. Fourth, the

most justified single value of γ is $\hat{\gamma} = \frac{\log 3}{\log 2}$ because it assigns an equal probability $(\frac{1}{3})$ for each state when the input CDF $y_{ij} = 0.5$. The marginal quantization curves indeed resemble the quantization curves generated by $\hat{\gamma}$.

These procedures convert a data matrix into a data probability tensor. For visualization convenience, we also reported the rank-transformed CDF matrix. Figure X5 illustrates the probabilistic quantization procedures.

Figure X5: Illustration of probabilistic quantization.

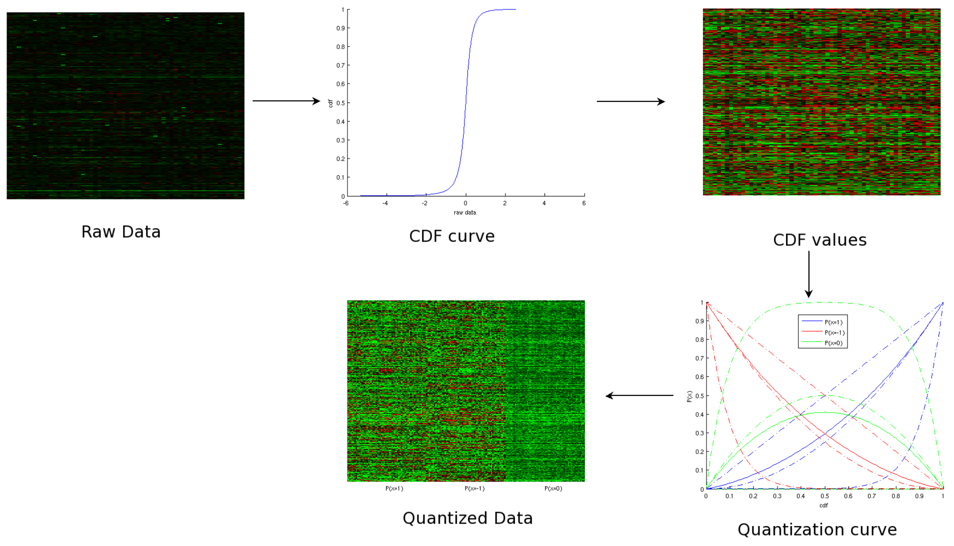

Genes are the elementary units in our analysis. For each data type, we aggregated all the relevant datasets and generated a gene-based matrix (tensor) reporting the CDF value (or

probability vector) of each gene in each sample. Multiple measurements of the same genes take place at two levels. First, there are multiple datasets generated by multiple institutions with different platforms. For instance, in GBM there are 4 mRNA expression datasets using Illumina HiSeq (1), Agilent G4502A microarrays (2), Affymetrix U133A microarrays (1). Second, a gene is often measured by multiple probes within a dataset. For instance, in an Affymetrix microarray the expression of a gene is measured by multiple probes. We aggregated multiple measurements of the same genes with two-step procedures. Within each dataset, we generated gene-level data by merging the probe data corresponding to the same gene. The CDF value of each gene in each sample is the average over all probe values of the corresponding gene and sample. The gene-based datasets from multiple sources are further merged into one dataset. Likewise, entries of the same gene and sample from multiple sources are merged by taking an average. The CDF values of the merged datasets are then converted into probability vectors with the aforementioned procedures. CNV and SNP data are not converted into gene-based matrices. CNV probes are partitioned into chromosomal segments (see the description below), whereas SNP loci are the basic units in the Association Models.

1.5 CNV data processing

The CNV data need to be processed separately as there are two unique problems when incorporating them in the Association Models. First, the aforementioned probabilistic quantization procedures overestimate amplification and deletion events. Second, TCGA CNV data are generated by dense CGH microarrays covering 941662 probes. The large number of probes both defy efficient construction of Association Models and are highly redundant. To resolve those problems, we modified probabilistic quantization to correct the

biases and further partitioned chromosomes into segments according to probe-level CNV data.

1.5.1 CNV data normalization

The underlying assumption of the probabilistic quantization procedure mismatches the empirical characteristics of CNV measurements. Equation 2 gives the probability of each trinary state x given the CDF value of its measurement outcome y. For each probe, y is uniformly distributed in $[0,1]$. Thus the probability of amplification events in the data ($x = +1$) is

$$P(x = +1) = \int_0^1 P(y)P(x = +1|y)\, dy = \int_0^1 \frac{y}{1-\log y}\, dy \approx 0.3663. \qquad (3)$$

Similarly, $P(x = -1) \approx 0.3663$ and $P(x = 0) \approx 0.2674$. These probabilities indicate that more than two thirds of the probe-level CNV data encounter amplifications or deletions. In reality, only a small fraction of CNV data entries deviate from normal values: about 1% of the data points have $\log_2$ ratios ≥ 1 or ≤ -1. Therefore, the quantization function in equation 2 severely distorts the global characteristic of the CGH array data.

To reduce this distortion we introduced an extra parameter β to the marginal quantization functions in equation 2:

$$P\left(x_{ij} = +1 \middle| y_{ij}\right) = \frac{{y_{ij}}^{\beta}}{1 - \log\left({y_{ij}}^{\beta}\right)}$$

$$P\left(x_{ij} = -1 \middle| y_{ij}\right) = \frac{(1-y_{ij})^{\beta}}{1-\log\left((1-y_{ij})^{\beta}\right)} \qquad (4)$$

$$P\left(x_{ij} = 0 \middle| y_{ij}\right) = 1 - P\left(x_{ij} = +1 \middle| y_{ij}\right) - P\left(x_{ij} = -1 \middle| y_{ij}\right)$$

The new $P\left(x_{ij} = +1 \middle| y_{ij}\right)$ and $P\left(x_{ij} = -1 \middle| y_{ij}\right)$ shrink with increasing β values. We adjusted β to make the global distribution $(P(x = -1), P(x = 0), P(x = +1))$ obtained from equation 4 close to the empirical distribution. For each CGH dataset, we counted the fractions of entries exceeding $\log_2\left(\frac{3}{2}\right) = 0.585$ (f_1) and below $\log_2\left(\frac{1}{2}\right) = -1$ (f_2). For simplicity we set the global empirical probability of amplification and deletion to be equal $P(x = +1) = P(x = -1) = \frac{1}{2}(f_1 + f_2)$. We then determine the parameter value $\hat{\beta}$ that fit the following equality:

$$\int_0^1 \frac{y^{\hat{\beta}}}{1 - \log\left(y^{\hat{\beta}}\right)}\, dy \approx P(x = +1)$$

$$\int_0^1 \frac{(1-y)^{\hat{\beta}}}{1 - \log\left((1-y)^{\hat{\beta}}\right)}\, dy \approx P(x = -1) \qquad (5)$$

The estimated parameter $\hat{\beta}$ is substituted in equation 4 in adjusted probabilistic quantization. The adjusted probability vector is substituted back in equation 2 and converts into an adjusted CDF value. Figure X6 demonstrates the quantization outcomes of BLCA chromosome 1 CNV data. The CNV data (top rows) have sparse entries undergoing amplifications or deletions, and the $\log_2$ ratios are sharply concentrated in 0. After ordinary probabilistic quantization (equation 2, middle rows), many more entries undergo copy number alterations, and the CDF values are uniformly distributed in [0,1], which contradicts with the observations from the raw data. In contrast, after adjusted probabilistic quantization (equation 4, bottom rows), amplification and deletion entries become sparse again, and the adjusted CDF values are sharply concentrated in 0.5.

Figure X6: Adjusted quantization outcomes of BLCA chromosome 1 CNV data. The top row displays the distribution of the $\log_2$ probe values and their heat map. The middle row displays the distribution of the CDF values and their heat map. The bottom row displays the distribution of the adjusted CDF values and their heat map.

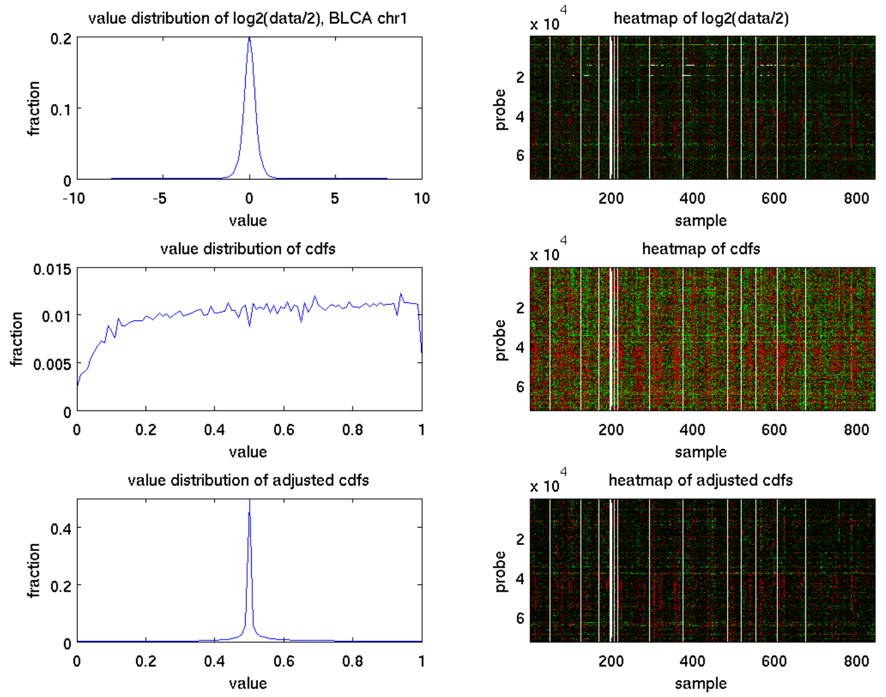

1.5.2 Generating segment CNV data

It is neither necessary nor sufficient to construct Association Models with the high-dimensional probe-level CNV data. Most CNV events cover long stretches of DNAs or even the entire chromosome arms. Moreover, many CNV events recur in multiple patients of the same cancer type. These properties of copy number variations enable us to partition chromosomes into a small number of segments and reduce the near one million dimensional

probe-level CNV data into an about one hundred dimensional segment-level CNV data. We proposed an algorithm to perform this dimension reduction task. In brief, it comprises three major parts: (1) partition the chromosomes of each sample into segments, (2) merge the segment boundaries from multiple samples to form global segment boundaries of the entire dataset, (3) generate segment-level CNV data.

Partitioning the chromosomes of each sample into segments

The input data of this part are the adjusted probability vectors of all CNV probes in a sample. We sorted probes by their coordinates and considered the probe data of one chromosome each time. A segment is a collection of consecutive probes on the same chromosome. We assumed that the CNV data of all probes on the same segment were independently drawn from a multinomial distribution over the hidden states $(-1,0,1)$. The CNV measurement of a probe gives a fractional count of each hidden state rather than a unit count to the most likely hidden state. The log likelihood of a segment can be thus evaluated. Formally, suppose a segment consists of m probes $1,\cdots,m$, and $\boldsymbol{p}_i \equiv (p_i^{-1}, p_i^0, p_i^1)$ denotes the adjusted probability vector of probe i. Define $\boldsymbol{N}_{1-m} = (N_{1-m}^{-1}, N_{1-m}^0, N_{1-m}^1) \equiv \sum_{i=1}^{m} \boldsymbol{p}_i$ the total counts of the hidden states over probes $1-m$, and $\boldsymbol{P}_{1-m} = (P_{1-m}^{-1}, P_{1-m}^0, P_{1-m}^1) \equiv \frac{\boldsymbol{N}_{1-m}}{\sum_{k=-1}^{1} N_{1-m}^k}$ its normalized probability vector. $\boldsymbol{P}_{1-m}$ gives the multinomial distribution of the fractional counts in probes $1-m$. The log likelihood of the probe data becomes $L_{1-m} \equiv \sum_{k=-1}^{1} N_{1-m}^k \log P_{1-m}^k$. A partition $(1,\cdots,i)(i+1,\cdots,m)$ splits the m probes into two segments. It adds two degrees of freedom to the model (one more multinomial distribution with three components). Thus the BIC score of the partition becomes $L_{1-i} + L_{(i+1)-m} - L_{1-m} - 0.5 \cdot 2 \cdot \log m$. Based on the BIC scores, we initially included all probes on a

chromosome in a segment S and incurred a function $\text{partition}(S)$ to recursively partition S into smaller segments:

Figure X7: $\text{partition}(S)$ algorithm to partition a chromosomal segment

Input: Adjusted probability vectors $\boldsymbol{p}_i$ for probes $i = 1 - |S|$.

Output: A partition of S into smaller segments.

Procedures:

1. Evaluate L_S.
2. Find the binary partition (S_1, S_2) of S that maximizes $L_{S_1} + L_{S_2}$.
3. Stop if the BIC score $L_{S_1} + L_{S_2} - L_S - 0.5 \cdot 2 \cdot \log m \geq 0$.
4. Otherwise incur $\text{partition}(S_1)$ and $\text{partition}(S_2)$.

Merging the segment boundaries from multiple samples to form global segment boundaries

There are three types of CNV events in cancers. Recurrent aneuploidies include frequent amplifications/deletions of a long stretch of DNA (often the entire chromosome arm) that occur in many tumors. Recurrent focal CNV events include frequent amplifications/deletions of a narrow chromosomal segment which typically harbors oncogenes or tumor suppressors. Sporadic CNV events occur randomly and infrequently on chromosomes. The segments generated by the first two types of CNV events are representative for all (or most) tumors of a

cancer type. Those global segments can thus reduce the high-dimensional probe-level CNV data into a low-dimensional segment-level CNV data.

The segment boundaries of all samples manifest all those three types of CNV events. While the boundary positions of sporadic CNV segments are randomly distributed, the boundary positions of recurrent CNV segments are often concentrated in narrow ranges. Therefore, finding boundaries of global CNV segments amounts to detecting narrow bands encompassing dense sample segment boundaries. We proposed an algorithm to merge the segments from multiple samples according to this intuition. Conceptually, we subdivided a chromosome into windows and counted the number of boundaries within each window. A band of consecutive windows with high boundary counts contains a candidate location for a global segment boundary. This assertion depends on both the window size and the threshold of boundary counts. We chose the *persistent* bands whose boundary counts (1) far surpass the expected boundary counts according to a randomized null model and (2) remain significant over a wide range of window sizes and threshold values. The detailed procedures are described below.

Figure X8: mergebds algorithm to merge boundaries of all samples on the same chromosome

Input: Segment boundaries from all samples, ranges of window sizes (in terms of the numbers of probes), and boundary counts thresholds.

Output: Global segment boundaries.

Procedures:

1. Vary the window size and subdivide a chromosome into windows accordingly.
2. Count the number of boundaries in each window for each window size.
3. For each window size and threshold value, identify the bands of consecutive windows whose boundary counts surpass the threshold.
4. For each window size, identify the threshold value intervals which give rise to the same numbers of bands with dense segment boundaries. Identify the unique bands that appear in one or multiple threshold value intervals. For each unique band, document the threshold interval where it is valid.
5. Apply two filtering criteria to select the unique bands.
   5.1 The null model assumes that the boundaries are uniformly distributed among all the windows. The maximal boundary count of a unique band should be 5 standard deviations more than the mean according to the null model.
   5.2 Under the same null model approximate the count of boundaries in a window by a binomial distribution. The threshold interval of a unique band should exceed the mean difference of boundary counts between two randomly selected windows.
6. For each window size, combine the selected unique bands which are overlapped.
7. Find the selected unique bands which persistently appear in the highest number of window sizes. Merge the persistent bands which are overlapped.
8. Return the centers of the merged persistent bands as the global segment boundaries.

Figure X9 illustrates the procedures of the segment boundary merging algorithm using the LIHC chr1 CNV data. The segment boundaries from all samples are marked by yellow dots on the top left panel. They are concentrated in the centromere, middle of the p arm and

several other locations. The boundary densities are peaked in those locations and relatively robust against window sizes (middle and bottom left panels). We inferred the global segment boundaries and marked them in the probe-level CNV data (blue vertical lines in top right panel) as well as the boundary densities (black bars in middle and bottom right panels). The visualization of probe-level CNV data and global segment boundaries of all chromosomes in all cancer types are reported in Supplementary Data.

Figure X9: Illustration of CNV segmentation on LIHC chromosome 1 CNV data. The top row displays the adjusted CNV probe CDF values with sample-specific boundaries (yellow marks on left) and global segment boundaries (cyan lines on right). The middle and bottom rows display the densities of boundaries within windows of 300 and 400 probes (left) and the marks of global boundaries (right).

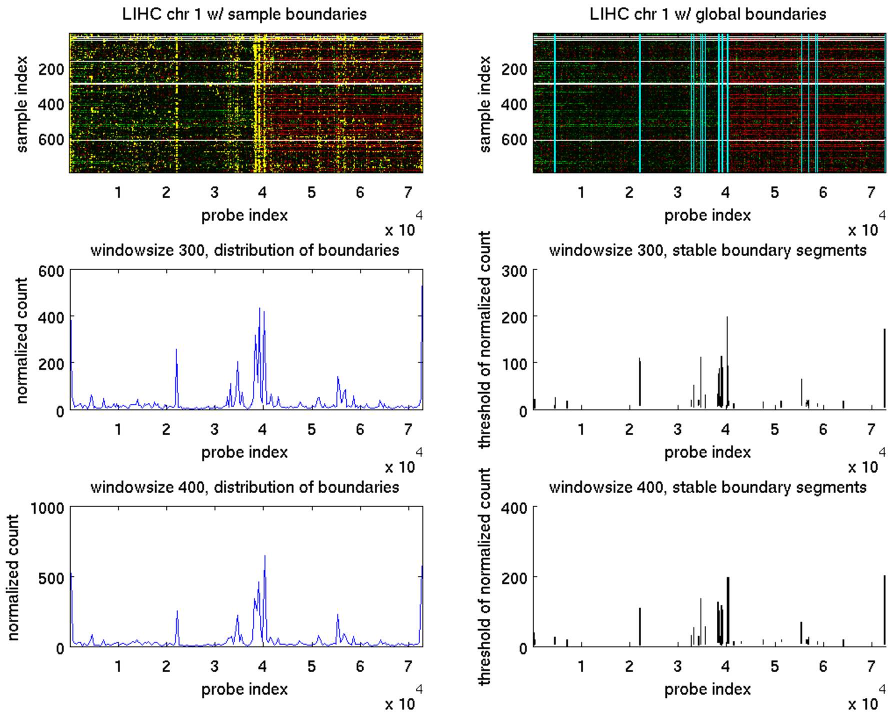

Generating segment-level CNV data

Given the global segment boundaries, it is straightforward to form global segments and infer their CNV data. Only one issue needs to take into account when generating global segments from their boundaries. A focal CNV segment is embedded within a long-range CNV segment. Thus the two sides of the long-range segment can have highly correlated CNV data but are separated by the focal segment boundaries. To fix this problem, we first partitioned a chromosome into segments according to the global segment boundaries, and then merged the non-adjacent segments with correlated CNV data. The adjusted CDF value of a segment CNV is simply the average over the CDF values of its constituent probes. Consequently, we generated segment-level CNV data directly from the probe-level CNV data.

The local and global segment boundaries of all chromosomes and all cancer types are reported in Supplementary Data.

1.6 Dimension reduction of molecular alteration data

The dimension of probe-level CNV data is greatly reduced by the aforementioned segmentation algorithm. Yet other types of molecular alteration data still possess hundreds or thousands of features, which are candidate effectors of the Association Models. Dimension reduction of candidate effectors is essential for association inference since (1) existence of multiple collinear features prevents identification of the true explanatory factors, (2) overfitting will likely occur when the data dimension far exceeds the sample size, (3) computational complexity of association inference also scales with the number of candidate

effectors. We adopted two dimension reduction approaches to the molecular alteration data. For DNA methylation and microRNA expression data, we clustered genes/mirs and used the average profiles over cluster members as candidate effectors. For all types of molecular alteration data, we also excluded the features which had either many missing entries or very little variation across samples.

1.6.1 Clustering DNA methylation and microRNA expression data

The TCGA DNA methylation data was generated by the Illumina HumanMethylation450 BeadChips. Methylation levels of cytosines on gene promoters were reported. We converted the probe-level DNA methylation data into gene-level data by taking the mean of the beta values over the probes on each gene promoter. The TCGA microRNA expression data was generated by RNAseq data and already summarized at gene levels.

We clustered the DNA methylation or microRNA expression data by combining clique finding and hierarchical clustering algorithms. Members within a clique are tightly correlated but relatively small. Thus we treated cliques as basic subunits and then applied hierarchical clustering to those subunits. The procedures are described below.

Figure X10: Clustering algorithm for DNA methylation and microRNA expression data.

Input: A DNA methylation or microRNA expression data matrix.

Output: Clusters genes or microRNAs of the data matrix.

Procedures:

1. Find cliques in terms of correlation coefficients between genes (or microRNAs).
   1.1 Sort correlation coefficients of gene pairs in a descending order.
   1.2 Subdivide the sorted correlation coefficient values into intervals with increment 0.001.
   1.3 Start with the top interval $[0.999,1]$ and construct a disconnected graph G with all genes/mirs as singleton nodes. Perform the following steps and decrement the interval. Stop when the interval becomes $[0.499,0.5]$.
      1.3.1 Add node pairs within the correlation coefficient interval as edges of G.
      1.3.2 Merge cliques obtained from the previous steps with the newly added edges in G.
      1.3.3 Generate new cliques from the newly added edges.
      1.3.4 Merge the two types of cliques generated from 1.3.2 and 1.3.3.
2. Apply hierarchical clustering to cliques in terms of Euclidean distances.
   2.1 Treat each clique as a cluster. Obtain the average DNA methylation/microRNA expression profile of each cluster. Calculate the Euclidean distances between the average profiles of cluster pairs.
   2.2 Continue merging clusters until the maximum correlation coefficient between cluster pairs < 0.3.
      2.2.1 Choose the cluster pair with the smallest Euclidean distance.
      2.2.2 Merge the selected cluster pair into one cluster. Update the average profile of the merged cluster and its Euclidean distances to other clusters.

1.6.2 Trimming molecular alteration data

We trimmed the features in the molecular alteration data that had either many missing entries or very little variation across valid samples. The feature selection criteria for each type of data are described below.

1. Mutation: A gene should have valid entries in $\geq 10\%$ and 10 of valid samples, and should have mutations in $\geq 1\%$ and 5 of valid samples.
2. DNA methylation: A gene should have valid entries in $\geq 20\%$ or 50 of valid samples, and should have hyper-methylated entries ($\beta \geq 0.7$) and hypo-methylated entries ($\beta \leq 0.3$) in $\geq 10\%$ or 10 of valid samples.
3. MRNA and microRNA expressions: a gene/mir should have valid entries in $\geq 25\%$ of valid samples, the number of nonzero entries $\geq 25\%$ of the number of zero entries, and the number of missing and zero entries $< 75\%$ of samples.
4. Keep all the protein expression and phosphorylation features.

1.7 Calculating shortest path distances between candidate effectors and targets in the unified network

To prioritize candidate effectors for target genes when building Association Models, we need to calculate the shortest distances between candidate effectors and targets in the unified network of molecular interactions. Effectors and targets are connected by valid paths which satisfy the following criteria: (1) the source (the first molecule in the path) is an effector in the TCGA data, (2) the destination (the last molecule in the path) is a target in the TCGA data, (3) the information flow direction along the path is consistent with the information flow

direction in each interaction, (4) the last portion of a valid path constitutes a regulatory link from a regulator to the target, such as ProteinDNA – DNARNA and ProteinDNA – MirRNA, (5) the path length ≤ 10, (6) the path does not self-intersect. Two challenges have to be addressed when calculating shortest path distances. First, it is intractable to enumerate the astronomical number of valid paths. Thus we need to calculate distances without listing valid paths. Second, some types of edges in the unified network have zero distances since they do not represent distinct steps in gene regulation. Instances of zero-distance edges include the edges of the central dogma information flow of the same genes (DNA,mRNA) (mRNA,protein), the edges from protein complex subunits to protein complexes and vice versa, and the edges from gene class members to gene classes and vice versa, all have zero distances. We proposed the following algorithm to calculate the shortest path distances between candidate effectors and targets.

Figure X11: Algorithm of calculating the shortest path distances.

Input: The unified network G, the maximum path length l_{max}.

Output: A sparse distance matrix D between molecule pairs. $D_{ij} = -1$ if the shortest path distance between i and j is 0, and $D_{ij} = 0$ if the distance between i and j is not considered.

Procedures:

1. Start with a sparse matrix D_R with all entries are 0s.

2. Find the molecule pairs connected by paths of zero distance. They include the paths consisting of the following types of interactions: DNA→mRNA→protein of the same genes, DNA→microRNA of the same microRNAs, gene class↔members, microRNA class↔members, protein complex↔members. Set their distances in D_R to -1.
3. Find the molecule pairs connected by edges of unit distance. They include edges in all other types of interactions. Set their distances in D_R to 1.
4. Identify the set of regulators R consisting of transcription factors and microRNAs. Set $d = 2$.
5. While $d \leq l_{max}$, repeat the following subroutines.

   5.1 Construct a sparse graph adjacency matrix G_1. $G_1(i,j) = 1$ if $D_R(i,j) = 1$ according step 3.

   5.2 Construct a sparse graph adjacency matrix G_2. $G_2(j,k) = 1$ if $D_R(j,k) = l_{max} - 1$ and k is a regulator.

   5.3 Compute $G_3 = G_1 \cdot G_2$. If $G_3(i,k) > 0$ and $D_R(i,k) = 0,$ then set $D_R(i,k) = l_{max}$.

   5.4 $d \leftarrow d + 1$.
6. Construct a sparse distance matrix D from D_R. For each pair of molecules (i,j), find the set of all regulators R_{ij}, such that for each $r \in R_{ij}, D_R(i,r) > 0, D_R(r,j) = 1$. $D(i,j) = \min_{r \in R_{ij}} D_R(i,r) + 1$.

1.8 Processing external data

The mRNA expression data of all external datasets (METABRIC, REMBRANDT, GEO data, CCLE, Illumina Bodymap) undergo rank transform and probabilistic quantization

analogous to the normalization procedures for TCGA mRNA expression data. The CNV data of all external datasets (METABRIC, REMBRANDT, CCLE) also undergo rank transform and adjusted probabilistic quantization analogous to the normalization procedures of TCGA CNV data. We did not apply the chromosome partitioning algorithm to the CNV data of external datasets. Instead, the CNV segments of TCGA BRCA and GBM data are used in validating METABRIC and REMBRANDT data. CCLE CNV data provides the chromosomal segments. We thus used the CCLE CNV segment data for validation. Other types of CCLE omic data that appeared in TCGA (mutations, DNA methylations, microRNA expressions, protein expressions and phosphorylations) underwent the same processing analogous to TCGA data. CCLE IC_{50} drug response data and Achilles gene dependency data, underwent rank transform.

The Illumina Bodymap data comprises 16 samples from distinct tissue types. We defined a gene specifically expressed in a tissue if its CDF value was at least two folds as those of the remaining tissues. The 16 tissue types and the number of tissue-specific genes for each tissue are listed in Table X3.

Table X3: Numbers of tissue-specific genes in Illumina Bodymap

index	tissue	# genes
1	adipose tissue	98
2	adrenal gland	994
3	brain	1362
4	breast	214
5	colon	62

6	heart	105
7	kidney	232
8	leukocyte	865
9	liver	385
10	lung	367
11	lymph node	369
12	ovary	518
13	prostate gland	297
14	skeletal muscle tissue	214
15	testis	1870
16	thyroid gland	542

The Roadmap Epigenomic data input comprises the chromosomal segments labeled with the 25 predicted epigenomic states listed on Table X2 for all samples. To further simplify the data we converted these labeled segments into the binary states of active transcription for all genes over all samples (tissue types). The algorithm below describes the conversion into binary active transcription states. Generally, we required that the epigenomic structure of a gene comprised transcription start sites, strongly or weakly transcribed elements, and consistent directions of promoters, 5' and 3' sites.

Figure X12: Algorithm of converting the segments labeled with epigenomic states into the active transcription states of individual genes.

Input: Labeled segments from a tissue type overlapped with the span of a gene.

Output: The binary active transcription state of the gene.

Procedures:

If the gene satisfies the following conditions, then assign its active transcription state to 1. Otherwise assign its active transcription state to 0.

1. It has a TssA segment near the start site of the gene. Its distance to the start site $\leq \frac{1}{3}$ of the gene length.
2. It has a Tx or TxWk segment downstream of the TssA segment.
3. If it has a PromU and (PromD1 or PromD2) sites, then some PromU is in the upstream of some (PromD1 or PromD2) sites. Some PromU is in the upstream of TssA, some (PromD1 or PromD2) is in the downstream of TssA.
4. If it has Tx5' and Tx3' sites, then some Tx5' is in the upstream of some Tx3'. Some of both Tx5' and Tx3' sites are downstream of the TssA segment.

2 Inferring IHAS from TCGA data

2.1 Association Models

2.1.1 An exponential family model

We specified the association between effectors and a target gene expression by an exponential family model. The model resembles logistic regression but allows associations

with discrete and continuous independent random variables. Denote y a target gene expression vector over samples, and $\boldsymbol{x} = (x_1, \cdots, x_F)$ its effector vectors. The conditional probability $P(y|\boldsymbol{x})$ is expressed as

$$P(y|\boldsymbol{x}) = \frac{1}{Z(x)} e^{\sum_{i=1}^{F} \lambda_i f_i(x_i) y}, \lambda_i \geq 0 \ \forall i. \qquad (6)$$

$f_i(x_i)$ is the feature function relating the effector value to the target value. If the effector input is a numerical variable (segment CNV, DNA methylation, microRNA expression, protein phosphorylation), then only two functions are allowed: $f_i(x_i) = x_i$ and $f_i(x_i) = -x_i$ denote that the effector (x) activates or represses the target gene expression (y) respectively. If the effector input is a categorical variable (mutation and SNP), then the following twelve functions in Table X4 are allowed:

Table X4: The possible feature functions of categorical variables.

index	$x = 0$	$x = 1$	$x = 2$	index	$x = 0$	$x = 1$	$x = 2$
f_0	1	0	−1	f_6	1	−1	−1
f_1	1	−1	0	f_7	−1	1	1
f_2	−1	0	1	f_8	−1	1	−1
f_3	−1	1	0	f_9	1	−1	1
f_4	0	1	−1	f_{10}	−1	−1	1
f_5	0	−1	1	f_{11}	1	1	−1

If x stands for a mutation variable, then roughly f_2, f_3, f_7, f_{10} are activator functions, and f_0, f_1, f_6, f_{11} are inhibitor functions, while the directions of other functions are ambiguous. $Z(\boldsymbol{x}) = \sum_{y=-1}^{1} e^{\sum_{i=1}^{F} \lambda_i f_i(x_i) y}$ is the partition that normalizes the conditional probabilities.

We chose this exponential family model instead of other common models (such as linear or nonlinear regressions, Bayesian networks, conditional random fields and neural networks) to build Association Models for several reasons. It contains combinatorial feature functions (such as those in Table X4) directly in its formula (equation 6). Other models may accommodate similar feature functions but require more complicated formulations. An exponential family model can incorporate additional covariates by multiplying exponential feature functions. Parameter estimation is achieved by a straightforward gradient descent algorithm (see the description below), which can be efficiently implemented and amenable for parallelization.

Although an Association Model is based on logistic regression, it differs from regular logistic regression models in several aspects. In standard logistic regression, the dependent variable (y) is a discrete random variable specifying the category of an event. In an Association Model, y is essentially a continuous random variable (gene expression level) but converted into a trinary random variable through probabilistic quantization. In standard logistic regression, parameters (λ_i's in equation 6) are estimated by applying an optimization algorithm directly to the data. In building associations from the multi-omics data, a large number of candidate effectors are correlated. To find the effector molecular alterations which likely modulate target gene expressions, we incurred a series of filtering and model selection processes (see the descriptions below).

Recall that we converted the effector and target feature values into tristate probability vectors. The converted probability vector of a feature value is viewed as fractional counts of possible states for an observed data point. For instance, if the CDF value y of an mRNA

expression value is 0.8, then according to equation 2

$\big(P(x = -1|y = 0.8), P(x = 0|y = 0.8), P(x = -1|y = 0.8)\big) = (0.0766, 0.2693, 0.6541)$.

Thus this observed value assigns the fractional counts $0.0766, 0.2693, 0.6541$ to the trinary states $x = -1, 0, 1$ respectively.

We introduce the following notations to represent the observed data and the log likelihood function in terms of fractional counts. Suppose there are m samples, and effectors $\boldsymbol{x}_j = (x_{1j}, \cdots, x_{Fj})$ and target y_j are the observed CDF (or categorical) values on sample j. Each x_{ij} $(i = 1, \cdots, F)$ is converted into fractional counts $q_{ijk}, k = -1, 0, 1$, and each y_j is converted into fractional count ρ_{jk} according to non-adjusted or adjusted probabilistic quantization (equation 2 or 4). There are 3^F configurations of effector feature values where each component effector takes values in $\{-1,0,1\}$. Likewise there are 3^{F+1} configurations of effector and target feature values. Denote $C_{\boldsymbol{x}} = (c_1, \cdots c_F)$ a configuration of effector feature values, and $C_{\boldsymbol{x},y} = (c_1, \cdots c_F, c_{F+1})$ a configuration of effector and target feature values, with each $c_i \in \{-1,0,1\}$. The fractional counts of configurations $C_{\boldsymbol{x}}, C_y$ and $C_{\boldsymbol{x},y}$ over all samples are

$$N(C_{\boldsymbol{x}}) = \sum_{j=1}^{m} \prod_{i=1}^{F} q_{ijc_i}.$$

$$N(C_y) = \sum_{j=1}^{m} \rho_{jc_{F+1}}.$$

$$N(C_{\boldsymbol{x},y}) = \sum_{j=1}^{m} \prod_{i=1}^{F} q_{ijc_i} \cdot \rho_{jc_{F+1}}. \qquad (7)$$

The log likelihood function of the observed data D is summed over possible configurations:

$$\mathcal{L}(D) = \sum_{j=1}^{m} \log P(\boldsymbol{x}_j) - \log Z(\boldsymbol{x}_j) + \sum_{i=1}^{F} \lambda_i f_i(x_{ij}) y_j$$

$$= \sum_{C_{x,y}} \{ N(C_x) \cdot \log P(C_x) + N(C_{x,y}) \cdot [- \log Z(C_x) + \textstyle\sum_{i=1}^{F} \lambda_i \, f_i(c_i) c_{F+1}] \}. \quad (8)$$

where the prior $\log P(C_x)$ is uniform over the effector configurations C_x. $f_i(.)$ is the feature function of effector i on the target gene expression, as described in equation 6 and Table X4.

The parameters $\Lambda = (\lambda_1, \cdots, \lambda_F)$ are estimated by maximizing the log likelihood function $\mathcal{L}(D|\Lambda)$. We first derive the gradient $\nabla\mathcal{L}(D|\Lambda)$ and Hessian $\mathcal{H}(D|\Lambda)$ of $\mathcal{L}(D|\Lambda)$.

$$\nabla\mathcal{L}(D|\Lambda) \equiv \left(\frac{\partial\mathcal{L}(D|\Lambda)}{\partial\lambda_1}, \cdots, \frac{\partial\mathcal{L}(D|\Lambda)}{\partial\lambda_F} \right),$$

$$\frac{\partial\mathcal{L}(D|\Lambda)}{\partial\lambda_i} = \sum_{C_{x,y}} N(C_{x,y}) \cdot \{ C_y \, f_i(c_i) - \frac{f_i(c_{x_i}) \cdot \left[e^{\sum_{j=1}^{F} \lambda_j f_j(c_{x_i})} - e^{-\sum_{j=1}^{F} \lambda_j f_j(c_{x_i})} \right]}{1 + e^{\sum_{j=1}^{F} \lambda_j f_j(c_{x_i})} + e^{-\sum_{j=1}^{F} \lambda_j f_j(c_{x_i})}} \}. \quad (9)$$

$$\mathcal{H}_{ij}(D|\Lambda) \equiv \frac{\partial^2\mathcal{L}(D|\Lambda)}{\partial\lambda_i \partial\lambda_j}$$

$$= \sum_{C_{x,y}} -N(C_{x,y}) \cdot \frac{f_i(c_{x_i}) f_j(c_{x_j}) (e^{\sum_{j=1}^{F} \lambda_j f_j(c_{x_i})} + e^{-\sum_{j=1}^{F} \lambda_j f_j(c_{x_i})} + 4)}{(1 + e^{\sum_{j=1}^{F} \lambda_j f_j(c_{x_i})} + e^{-\sum_{j=1}^{F} \lambda_j f_j(c_{x_i})})^2}. \quad (10)$$

The maximum likelihood parameter $\widehat{\Lambda}$ was numerically estimated using Newton Raphson's method. Set an initial value $\Lambda^0 = (1, \cdots, 1)$. Iteratively execute the following updates until convergence.

$$\Gamma^t = \Lambda^t - \nabla\mathcal{L}(D|\Lambda^t) \cdot \mathcal{H}^{-1}(D|\Lambda^t).$$

$$\lambda_i^{t+1} = \max(\gamma_i^t, 0). \tag{11}$$

The maximum log likelihood value can be evaluated by substituting $\widehat{\Lambda}$ back to equation 8.

When building an Association Model, we incrementally added candidate effectors that provided the best extra explanatory power to the data given the current list of effectors. This procedure requires comparison of the log likelihood values between the current model and a new model by adding one candidate effector to the current model. We quantified the model comparison results with two statistical approaches. Given the observed data D and two nested models $M_0, M_1 \supseteq M_0$, we incurred a standard hypothesis testing procedure to calculate the log likelihood ratio and χ^2 p-value:

$$\mathcal{L}(D; M_0, M_1) = \mathcal{L}(D|M_1) - \mathcal{L}(D|M_0).$$

$$p = 1 - \chi_d^2\big(2\mathcal{L}(D; M_0, M_1)\big). \tag{12}$$

where χ_d^2 is the χ^2 CDF function with d degrees of freedom, and d is the number of extra free parameters in M_1 relative to M_0. Here $d = 1$ as M_1 has one more effector (thus free parameter) than M_0.

The χ^2 p-values tend to over-estimate the significance of the testing results as they are asymptotic approximations when sample size approaches infinite. Thus we also evaluated the p-values of permutation tests and reported the supremum of χ^2 and permutation p-values. To assess the statistical significance of the extra explanatory power of the additional effector in M_1, we fixed the data of all variables in M_0 (including effectors and the target) and permuted only the data of the additional effector in M_1. Also, albeit the log likelihood ratio captures the quantitative difference of two models to fit the data, it may fail to distinguish the

qualitative difference under some circumstances. For instance, in scenario 1, the two models may predict the probability of up-regulation of a target gene to be 0 and 0.3; in scenario 2 the predicted probabilities of up-regulation are 0.3 and 0.6 respectively. While the differences of predicted probabilities are identical, the two scenarios have distinct qualitative implications. Scenario 1 suggests that the target gene is unlikely up-regulated according to the predictions of both models, yet scenario 2 indicates that one model predicts the target gene to be up-regulated with a moderate probability but another model predicts the opposite direction. To accommodate both quantitative and qualitative differences, we proposed two test statistics in calculating permutation p-values: the log likelihood ratio and the difference between consistent and inconsistent samples according to model prediction. The latter used hard thresholds to convert the CDF data into trinary states and applied the feature functions to determine whether the trinary configuration of a sample was consistent or not. This counting is less precise than the log likelihood ratio but can capture the aforementioned qualitative difference. The permutation p-value of each type is the fraction of permutations where the test statistic values exceed the empirical values. The reported permutation p-value is the supremum of the two p-values. Permutation p-values were calculated by the following procedures:

Figure X13: Algorithm of calculating permutation p-values.

Input: Effector and target variable CDF values over the samples, the association models M_0 and M_1 of the data.

Output: The permutation p-value.

Procedures:

1. Quantize the numerical $\boldsymbol{x}$ (effector CDF) and y (target CDF) values into trinary states, with 0.4 and 0.6 as thresholds for -1 and $+1$ states. Leave the categorical effector values intact.
2. In the quantized data, check each sample whether y value is consistent with the predicted values according to $\boldsymbol{x}$ values and the feature functions of the model M_1. Report the difference of the counts of consistent and inconsistent samples. Denote this number as n_C.
3. Identify the newly added effector x_v that appears in M_1 but not in M_0.
4. Repeat the following steps 10000 times.

   4.1 Randomly permute the data in x_v.

   4.2 Report the difference of the counts of consistent and inconsistent samples in the permuted data and denote this number as n_P.

   4.3 Report the log likelihood ratio of the permuted data.
5. The p-value p_1 is the fraction of the random permutations whose n_P's exceed n_C.
6. The p-value p_2 is the fraction of the random permutations whose log likelihood ratios exceed the log likelihood ratio of the empirical data.
7. The p-value is the supremum of p_1 and p_2.

2.1.2 Selecting candidate effectors according to pairwise associations

Building Association Models of the entire TCGA data is computationally intensive as all combinations of candidate effectors and target mRNA expressions in all cancer types have to be considered. Most of those candidate effectors have quite weak pairwise associations with the target mRNA expressions and will unlikely appear in the Association Models. To avoid wasting computing resources on unlikely associations, we filtered candidate effector-target associations according to pairwise association outcomes. In each cancer type we calculated pairwise associations between all candidate effectors (CNVs of all chromosomal segments, mutation profiles of all genes, DNA methylation profiles of all genes, microRNA expression profiles of all microRNAs, phosphorylation profiles of selected proteins, SNPs of all probes) and all target gene expressions. Three statistical quantities for each pairwise association are reported: log likelihood ratios of logistic regression model hypothesis testing, supremum of χ^2 and permutation p-values, and correlation coefficients between candidate effectors and target gene expressions. For each target gene expression, we kept the candidate effectors whose scores of all three types (log likelihood ratios, p-values, correlation coefficients) surpassed predetermined threshold values. Only selected candidate effectors are considered when constructing the joint Association Models. Computation of all pairwise associations is executed in parallel in a Dell Precision 7920 Tower Workstation with 28 cores and 100 nodes. The total computing time is about one week (16800 CPU-hours).

The threshold values of the three statistical scores are not fixed for all cancer types since they are sensitive to sample sizes and different cancer types have a wide range of sample sizes. Instead we determined those threshold values by controlling the numbers and rates of false discoveries. False discovery rates (FDRs) are calculated by a general form below:

$$\mathrm{FDR}(\theta) = \frac{\frac{\#\,\text{random associations with scores} \geq \theta}{\#\,\text{random associations}}}{\frac{\#\,\text{associations with scores} \geq \theta}{\#\,\text{associations}}}. \qquad (13)$$

The denominator counts the fraction of pairwise associations from the real data whose scores surpass a threshold θ, and the numerator counts the fraction of pairwise associations from the randomized data whose scores surpass θ. We generated randomized data by randomly permuting the effector and target mRNA expression data 10000 times. Since the number of random permutations is rather limited, the extreme values of the statistical scores generated from the randomized data (the highest log likelihood ratios and correlation coefficients and the lowest p-values) are likely much less significant than those generated from the empirical data. This property will force $\mathrm{FDR}(\theta) = 0$ when θ surpasses the extreme value of the randomized associations. To overcome this limitation, we obtained the statistical score distribution of the randomized data from both the empirical distribution from 10000 random trials and a parametric distribution that fit the empirical distribution. The latter may have nonzero tail probabilities beyond the extreme value from the empirical distribution. We fit the distributions of log likelihood ratios, negative log p-values, and absolute values of correlation coefficients as mixtures of a constant random variable at value 0 and generalized Pareto, Weibull, and Nakagami distributions respectively. Denote the FDRs evaluated by the empirical and estimated score distributions from randomized data $\mathrm{FDR}_{\mathrm{emp}}$ and $\mathrm{FDR}_{\mathrm{est}}$ respectively. The modified FDR reports the minimum of the two FDRs if both have positive values and the nonzero entry if only one has a positive value:

$$\mathrm{FDR}(\theta) = \min\left(FDR_{emp}, FDR_{est}\right) \text{ if } FDR_{emp} > 0 \text{ and } FDR_{est} > 0.$$

$$= \max(FDR_{emp}, FDR_{est}) \text{ if } FDR_{emp} > 0 \text{ xor } FDR_{est} > 0. \quad (14)$$

$$= 0 \text{ if } FDR_{emp} = 0 \text{ and } FDR_{est} = 0.$$

The estimated number of false discoveries (nFD) is the product of FDR and the number of pairwise associations whose scores surpass the threshold θ.

Given the FDR's and nFD's with varying threshold values, we determined the threshold values of log likelihood ratios, p-values and absolute correlation coefficients according to the following procedures.

Figure X14: Criteria for determining the threshold values for log likelihood ratios, p-values and absolute correlation coefficients.

log likelihood ratios: Find a threshold value that satisfies the following properties.

- DNA methylation, microRNA expression, protein phosphorylation, and SNP:
  1. Find the log likelihood ratios that suddenly raise FDR's and nFD's respectively (the rates of change are maximized). Choose the minimum of those two values.
  2. If the number of association pairs (from the empirical data) corresponding to this threshold ≥ 10000 (≥ 1000 for SNP associations), then choose the maximum threshold whose number of association pairs ≥ 10000 (≥ 1000 for SNP associations).
  3. If no such threshold exists, then choose the maximal log likelihood ratio from the empirical data as the threshold.

4. If this threshold exceeds the upper limit of the threshold, then set the threshold to the upper limit. The upper limit depends on the types and levels of associations.

- Trans-acting CNV and mutation:
  1. Find the threshold values whose FDR's $\leq 10^{-3}$ and the number of pairwise associations ≥ 50000.
  2. If those threshold values do not exist, then find the threshold values whose FDR's $\leq 10^{-3}$ and the number of pairwise associations ≥ 10000.
  3. If those threshold values exist and the smallest threshold value is below an upper limit, then report the minimum of the candidate threshold values. The upper limit depends on the type and level of associations.
  4. If those threshold values exist but the smallest threshold value exceeds an upper limit, then report upper limit as the threshold value.
  5. If those threshold values do not exist, then check whether the FDR's jump at a threshold value below an upper limit. If yes, then report the smallest threshold value of the jumps.
  6. If there are no FDR jumps or the jumps are above the upper limit, then find the local minimum of FDR's whose threshold values are below the upper limit and the number of pairwise associations ≥ 10000. Report the corresponding threshold value.
  7. If this range of threshold values still does not exist, then find the threshold values below the upper limit and identify the one which minimizes the FDR. Report this threshold value.

Minus log p-values: Find a threshold value that satisfies the following properties.

- DNA methylation, microRNA expression, protein phosphorylation, and SNP:
    1. Find the threshold values whose FDR’s and nFD’s are the minimum respectively, find the minimum of those two threshold values.
    2. If the number of association pairs (from the empirical data) corresponding to this threshold ≥ 10000 (≥ 1000 for SNP associations), then choose the maximum threshold whose number of association pairs ≥ 10000 (≥ 1000 for SNP associations).
    3. If this threshold exceeds the upper limit of the threshold, then set the threshold to the upper limit.
- Trans-acting CNV and mutation:
    1. Find the threshold values whose FDR’s $\leq 10^{-3}$ and the number of pairwise associations ≥ 50000.
    2. If those threshold values do not exist, then find the threshold values whose FDR’s $\leq 10^{-3}$ and the number of pairwise associations ≥ 10000.
    3. If those threshold values exist and the smallest threshold value is below an upper limit, then report the minimum of the candidate threshold values.
    4. If those threshold values exist but the smallest threshold value exceeds an upper limit, then report upper limit as the threshold value.
    5. If those threshold values not exist, then find the threshold values below an upper limit and the corresponding number of pairwise associations ≥ 50000.
    6. If those threshold values not exist, then find the threshold values below an upper limit and the corresponding number of pairwise associations ≥ 10000.
    7. If those threshold values exist, then find the threshold value that gives the smallest FDR.

8. If this threshold exceeds the upper limit of the threshold, then set the threshold to the upper limit.

Absolute values of correlation coefficients: Find a threshold value that satisfies the following properties.

- DNA methylation, microRNA expression, protein phosphorylation:
  1. Find the threshold values whose FDR's and nFD's are the minimum respectively, find the minimum of those two threshold values.
  2. If the number of association pairs (from the empirical data) corresponding to this threshold ≥ 10000 (≥ 1000 for SNP associations), then choose the maximum threshold whose number of association pairs ≥ 10000 (≥ 1000 for SNP associations).
  3. If this threshold exceeds the upper limit of the threshold, then set the threshold to the upper limit.
- Trans-acting CNV:
  1. Find the threshold values whose FDR's $\leq 10^{-3}$ and the number of pairwise associations ≥ 50000.
  2. If those threshold values do not exist, then find the threshold values whose FDR' $\leq 10^{-3}$ and the number of pairwise associations ≥ 10000.
  3. If those threshold values exist and the smallest threshold value is below an upper limit, then report the minimum of the candidate threshold values.
  4. If those threshold values exist but the smallest threshold value exceeds an upper limit, then report upper limit as the threshold value.

5. If those threshold values not exist, then find the threshold values below an upper limit and the corresponding number of pairwise associations ≥ 50000.
6. If those threshold values not exist, then find the threshold values below an upper limit and the corresponding number of pairwise associations ≥ 10000.
7. If those threshold values exist, then find the threshold value that gives the smallest FDR.
8. If those threshold values do not exist, then find the threshold values below an upper bound and the corresponding FDR's $\leq 10^{-3}$. Find the minimum threshold value among them.
9. If those threshold values do not exist, then find the threshold values below an upper bound. Find the threshold with the minimum FDR.
10. If this threshold exceeds the upper limit of the threshold, then set the threshold to the upper limit.

- Mutation and SNP: do not apply filter on correlation coefficients.

Two examples of threshold determination are illustrated in Figures X15 and X16 for trans-acting CNV and DNA methylation associations in BRCA. They display the numbers of pairwise associations that surpass the threshold (nemppass), FDR's and nFD's with varying thresholds for log likelihood ratios, minus log p-values, and absolute values of correlation coefficients respectively. nemppass, $FDR's$ and $nFD's$ generally decline with more stringent threshold values. Yet $FDR's$ and $nFD's$ may jump upward at very stringent threshold values, probably due to the very small numbers of empirical and randomized association pairs. The chosen threshold values depend on the limits of the threshold values, nemppass, $FDR's$, as well as the shapes of the FDR/nFD curves. Those limits are reported in Table X5. The nemppass, $FDR's$ and $nFD's$ of all types of pairwise associations in all cancer types are

reported in Supplementary Data. Notice that the limits of FDR and nemppass are common for all types of associations, hence they exert the same level of control on the false discovery rates and the numbers of candidate pairwise associations. In principle, the threshold values in each cancer type can be completely determined by these limits and the procedures in Figure X14. To prevent the pathological situations where the derived threshold values are excessively loose (low threshold values on log likelihood ratios and correlation coefficients and high threshold values on p-values), we also explicitly set different limits of the threshold values on distinct types of associations in Table X5. The types of effectors that induce a large number of pairwise associations (such as SNPs and DNA methylations) typically have more stringent limits, while the types of effectors that induce relatively fewer pairwise associations (such as trans-acting CNVs) have less stringent limits. Although there are no strong justifications for these heuristically varying limits, they are likely inactive in most cancer types as the threshold values determined by Figure X14 are typically within the limits.

Figure X15: Dependency of nemppass, FDR's and $nFD's$ with respect to log likelihood ratios, log p-values, and correlation coefficients. BRCA trans-acting CNV segment associations. Red dots mark the chosen threshold values.

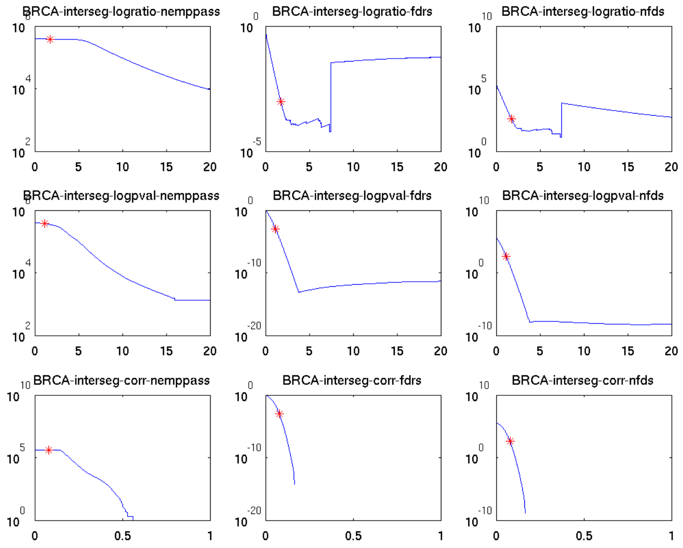

Figure X16: Dependency of nemppass, FDR's and $nFD's$ with respect to log likelihood ratios, log p-values, and correlation coefficients. BRCA DNA methylation associations.

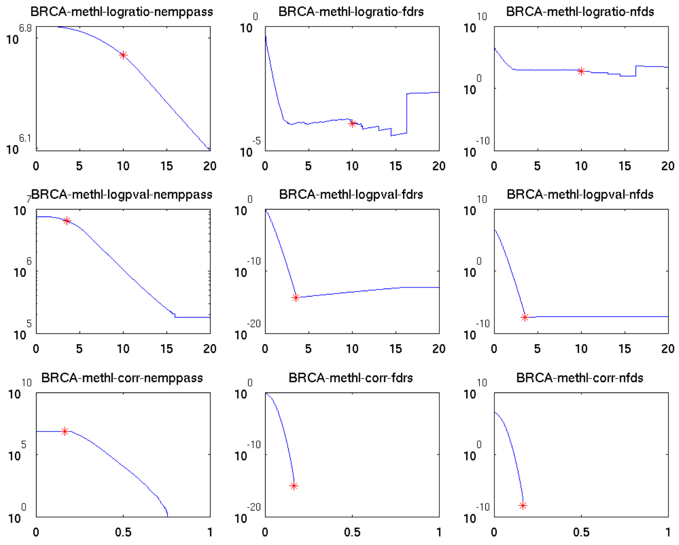

Table X5: Limits for determining threshold values.

type of limit	type of threshold	value	type of limit	type of threshold	value
upper FDR	All	10^{-3}	lower methylation assocs.	log likelihood ratio	10.0
lower nemppass 1	All	50000	upper methylation assocs.	p-value	10^{-5}
lower nemppass 2	All	10000	lower methylation assocs.	abs. corr.	0.3
lower nemppass, SNP	All	1000	lower mir assocs.	log likelihood ratio	10.0
lower cis-acting assocs.	log likelihood ratio	2.0	upper mir assocs.	p-value	10^{-5}
upper cis-acting assocs.	p-value	10^{-2}	lower mir assocs.	abs. corr.	0.3
lower cis-acting assocs.	abs. corr.	0.3	lower phos. assocs.	log likelihood ratio	5.0
lower cis-acting SNP assocs.	log likelihood ratio	4.0	upper phos. assocs.	p-value	10^{-4}
upper cis-acting SNP assocs.	p-value	10^{-5}	lower phos. assocs.	abs. corr.	0.3
lower cis-acting SNP assocs.	abs. corr.	NA	lower SNP assocs.	log likelihood ratio	4.0
lower trans-acting CNV assocs.	log likelihood ratio	4.0	upper SNP assocs.	p-value	10^{-5}
upper trans-acting CNV assocs.	p-value	10^{-2}	lower SNP assocs.	abs. corr.	NA
lower trans-acting CNV assocs.	abs. corr.	0.3			
lower mutation assocs.	log likelihood ratio	2.0			
upper mutation assocs.	p-value	10^{-1}			
lower mutation assocs.	abs. corr.	NA			

Once the threshold values for log likelihood ratios, p-values and absolute values of correlation coefficients were determined, we applied a joint filter to the pairwise associations from empirical and randomized data using those threshold values. The resulting nemppass, FDR's and nFD's of the joint filter were estimated in the same fashion as those filtered by each type of score. If the estimated FDR is larger than the estimated FDR's from single types of scores, then report the minimum FDR from single types of scores.

2.1.3 Statistical model selection

An Association Model uses the molecular alteration profiles of (zero, one or multiple) effectors to explain the mRNA expression profile of one target gene. The joint model is represented by an exponential family model $P(y|\boldsymbol{x})$. Building an Association Model can be viewed as a model selection problem with prior knowledge and assumptions about the causal relations of variables: finding the candidate effectors which likely affect the target gene expressions. Different from standard model selection problems, the candidate effectors have ordinal but not cardinal priorities. For instance, a candidate effector which connects to the target gene with path length 1 in the cascade of transcriptional regulation has a higher priority than another candidate regulator which connects to the target gene with path length 2. Also, a CNV candidate effector has a higher priority than a microRNA expression candidate effector. However, the relative weights of these candidate effectors in explaining the target gene expressions cannot be intuitively determined. We employed a stepwise regression-like algorithm to sequentially add covariates that provide the best explanatory power to the data conditioned on the existing model. The additional explanatory power is quantified by the log likelihood ratio and p-value of the augmented model relative to the existing model. The order of incorporating the covariates is consistent with their ordinal priorities according to

prior knowledge. In the context of IHAS inference, the sequential model selection algorithm is superior to batch model selection algorithms (such as regressions with sparsity constraints like lasso or elastic net) because the former can best incorporate ordinal priorities of candidate effectors. In contrast, batch algorithms require cardinal priorities of candidate effectors which are not directly available. Besides permutation p-values, it is also possible to execute cross validation like procedures to select the model: iteratively leaving out one effector from the model and check how good the model fits the data in each reduced model. A model has a robust prediction power if removing each single effector from the model does not considerably deteriorate performance. Yet these procedures are top-down and require the presence of a model. They are hence more apt for assessing the prediction power of a model than building a model.

Since computation time of parameter estimation and log likelihood and p-value evaluation scales with the dimension of the model, we adopted a series of filters to reduce the number of candidate effectors.

The ordinal priorities of candidate effectors were determined in a hierarchical fashion. They were first stratified by their shortest path distances to the target genes in the network of molecular interactions. Within each stratum, candidate effectors are ordered by the types of molecular alterations. Within each stratum and molecular alteration type, there can be still many candidate effectors. To further narrow down the candidate effectors, we incurred filtering processes for each type of molecular alteration effectors and their union. The precise procedures of statistical model selection are described below. Here we consider the model selection procedures in one iteration, as more involved iteration procedures will be discussed later.

Figure X17: select_effector(.), algorithm of selecting one effector that provides the highest additional explanatory power given an existing model.

Input: The mRNA expression profile y of the target gene. All candidate effector profiles $\boldsymbol{X}$. An existing model M_0 that fits y with effectors $\boldsymbol{x}$ and represents $P(y|\boldsymbol{x})$ with an exponential family model.

Output: One effector $\hat{x} \in \boldsymbol{X} / \boldsymbol{x}$ that provides the best additional explanatory power to fit the data.

Procedures:

1. Narrow down $\boldsymbol{X}$ to the effectors whose pairwise associations with y surpass the thresholds of log likelihood ratios, p-values and absolute correlation coefficients.
2. For trans-acting CNV segment associations in the candidate effectors, require the presence of at least one regulator. A regulator is a transcription factor or signaling protein which satisfies three conditions: (1) the regulator is located on the same CNV segment or chromosome arm, (2) there is a significant pairwise association between the segment CNV and the regulator mRNA expression profile, (3) there is a significant pairwise association between the regulator and target gene expression profiles. Keep the trans-acting CNV segment associations that have regulators.

3. For trans-acting SNP associations in the candidate effectors, require the presence of at least one regulator. A regulator requires the same conditions as above except in (1) it is located within 10Mb from the effector SNP.
4. Among the candidate segment CNVs on the same chromosome, pick the one with the strongest log likelihood ratio as the representative and discard the remaining candidate segment CNVs on the chromosome.
5. Among the candidate DNA methylation profiles in the same cluster, pick the one with the strongest log likelihood ratio as the representative and discard the remaining candidate DNA methylation profiles in the same cluster.
6. Pick the representative microRNA expression from each cluster in the same fashion as DNA methylation profile selection.
7. If there are ≥ 20 candidate effectors after the aforementioned filters, then calculate the gap score of each candidate effector. Subdivide samples into two groups according to effector states. For mutations, separate samples with or without the mutations. For SNPs, separate samples with the two homozygous genotypes. For other effectors, separate samples with high (≥ 0.6) and low (≤ 0.4) effector values. The gap score is the difference of mean target gene expressions between the two groups. Sort candidate effectors by their gap scores in a descending order and keep the top 20 effectors.
8. If there are still ≥ 20 candidate effectors after the filters, then filter them by conditional mutual information. For each pair of candidate effectors x_1 and x_2, calculate $\mathrm{MI}(y; x_1|x_2)$ and $\mathrm{MI}(y; x_2|x_1)$. If $\mathrm{MI}(y; x_1|x_2)$ is low but $\mathrm{MI}(y; x_2|x_1)$ is high, then y and x_1 are conditionally independent given x_2, and the explanatory power of x_1 is absorbed by x_2. Thus remove x_1. Repeat this procedure for all pairs of effectors.

9. Among the remaining candidate effectors, calculate the log likelihood ratios and p-values by adding each effector to the existing model. Filter out the candidate effectors whose log likelihood ratios and p-values do not surpass the pre-determined threshold values.
10. Among the remaining candidate effectors, remove the ones whose explanatory powers are overwhelmed by others. For each pair of candidate effectors x_1 and x_2, incur nested hypothesis tests to compare the bi-covariates model $x_1 \cup x_2$ against the uni-covariate models x_1 and x_2. Build a graph G_o of overwhelm relations between effectors from those hypothesis tests. $G_o(x_1, x_2) = 1$ and $G_o(x_2, x_1) = 0$ if x_1 overwhelms x_2: the joint model $x_1 \cup x_2$ is superior than x_2 but not superior than x_1. If $x_1 \cup x_2$ is superior to neither x_1 nor x_2, then $G_o(x_1, x_2) = 1$ and $G_o(x_2, x_1) = 1$. From G_o construct another directed graph G: $G(x_1, x_2) = 1$ if $G_o(x_1, x_2) = 1$ and $G_o(x_2, x_1) = 0$. The *sinks* of G are the nodes which have incoming but no outgoing edges in G. Those effectors are overwhelmed by other effectors but do not overwhelm others. Remove the sink effectors.
11. Add the remaining candidate effectors to the model.

A principled but much more time-consuming approach to select the effectors is to identify the *Markov blanket* of the target expression y. Suppose the current model comprises effectors $Z \equiv \{z_1, \cdots, z_k\}$. The conditional probability $p(y|z_1, \cdots, z_k)$ can be represented as a star-graph with edges pointing from each z_i to y. The selected candidate effectors $X \equiv \{x_1, \cdots, x_m\}$ at the current iteration include the covariates where the dependency of each effector x_i to y is not mediated by any subset of $Z \cup (X \backslash x_i)$. Mediation of a variable subset S between y and x_i can be detected by testing the nested model $p(y|S \cup x_i)$ against $p(y|S)$. It is expensive and often intractable to incur hypothesis tests to all possible subsets of existing

and candidate effectors. Therefore, the bulk of the select_effector(.) algorithm is to exert various filtering processes to limit the candidate effectors and subsets of covariates for mediation analysis. Step 1 selects the candidate effectors whose pairwise scores exceed the threshold values. Steps 2-6 filter the candidate effectors of trans-acting CNVs, trans-acting SNPs, cis-acting CNVs, DNA methylations, and microRNA expressions respectively. If there are more than 20 candidate effectors after step 6, then step 7 selects 20 of them with the largest variations of effector values across samples. Step 8 uses conditional mutual information as a surrogate for mediation analysis for each pair of candidate effectors. Step 9 conducts mediation analysis of $p(y|Z \cup x_i)$ against $p(y|Z)$ for each candidate effector x_i. Step 10 conducts mediation analyses of $p(y|x_i, x_j)$ against $p(y|x_i)$ and $p(y|x_j)$ for each candidate effector pair x_i, x_j. After step 10 a small number of candidate effectors pass all filtering criteria and are added to the Association Model.

2.1.4 Prioritizing candidate effectors using biological knowledge

The integrated TCGA data have a large number of highly dependent features. These properties make selection of true effectors of target genes challenging since there are many highly correlated candidates. The statistical model selection together with a series of filtering criteria can substantially reduce the number of candidate effectors, but cannot tell which of the remaining candidate effectors are biologically meaningful. To mitigate this problem, we prioritized candidate effectors in terms of the relevance and directness of their influences on the target genes. This prioritization subdivides candidate effectors into several categories, and candidate effectors from top to bottom categories are sequentially considered. Within each category, the aforementioned statistical model selection procedures are employed to incrementally build Association Models.

We prioritized candidate effectors in a hierarchical fashion. At the top tier, candidate effectors are ordered by whether there are direct, indirect or no evidence regarding regulatory relations with the target genes. At the middle tier, effectors with indirect regulatory relations are ordered by their shortest path lengths to the target gene in a unified network of gene regulation, metabolic reactions and molecular interactions. At the bottom tier, candidate effectors are ordered by the types of molecular alterations. Detailed criteria for prioritization are described below.

Top tier:

1. Level 1: Effectors are local to the target genes. They include cis-acting CNV segment associations (CNV segments encompass the target gene or on the same chromosome arm of the target gene), mutations and DNA methylations (mutated and methylated genes are the same as the target genes), and SNPs (SNPs are on the target genes or within 10Mb from them).
2. Level 2: Effectors are nonlocal to the target genes but connected to them by paths in the united network of gene regulation, metabolic reactions and molecular interactions. For CNV segments, consider the paths from their regulators to the target genes.
3. Level 3: Nonlocal effectors of CNV segments with positive associations, mutations, DNA methylations with negative associations, and SNPs, and are not connected to the target genes by paths in the united network.
4. Level 4: Nonlocal effectors of CNV segments with negative associations, and do not appear in lower-level associations.
5. Level 5: Nonlocal effectors of microRNA expressions with negative associations and phosphorylations, and do not appear in lower-level associations.

Middle tier:

Among the level 2 effectors, sort them by the shortest path lengths to the target genes in the unified network. Effectors with shorter path lengths have higher priorities.

Bottom tier:

Among the effectors in a category of top and middle tiers, sort them according to the following order.

1. CNV segments.
2. Mutations.
3. DNA methylations with negative associations.
4. MircoRNA expressions with negative associations.
5. Protein phosphorylations.
6. SNPs.

2.1.5 Summary of constructing Association Models

We synthesize the previously described methods and summarize the procedures of Association Model construction below.

Figure X18: Schematic description of constructing Association Models.

Input: Multimodal data of molecular alterations and gene expressions.

Output: Association Models for all the genes.

Procedures:

1. Pairwise associations:

   1.1 Incur pairwise associations between all candidate effectors of all data types and mRNA expressions.

   1.2 For each type of association pairs, randomly select 10000 pairs, permute the data, and compute pairwise association scores.

   1.3 Based on the pairwise association scores of both empirical and permuted data, evaluate nemppass, FDR$'s$ and nFD's with varying threshold values. Determine threshold values of pairwise associations accordingly.

2. Integrated associations:

   2.1 For each gene, find candidate effectors whose pairwise association scores surpass the threshold values.

   2.2 Sort and subdivide candidate effectors into categories according to the hierarchy of prioritization.

   2.3 In each category incur statistical model selection to identify the effectors for each gene.

   2.4 Build an exponential family model for each gene.

2.2 Association Modules

An Association Module constitutes three parts: a common effector, a collection of target genes sharing the common effector, and (for trans-acting CNV segment and trans-acting SNP associations) regulators mediating the influence from effectors to targets. All Association

Modules are immediately determined once the Association Models of all target genes are constructed. For each candidate effector, identify all the target genes whose Association Models contains it. Those target genes are members of the corresponding module.

There are modules of cis-acting CNV and trans-acting CNV associations with correlated target gene expressions and physically close segments. We adopted the following procedures to consolidate Association Modules of cis-acting and trans-acting segment CNV associations.

Figure X19: Algorithm of consolidating segment CNV Association Modules.

1. Identify the CNV segments on the same chromosome.
2. Cluster their segment CNV data with varying thresholds.
3. Extract all unique clusters and mark the threshold value combinations where they appear.
4. Find the clusters that harbor consecutive segments.
5. Group together the consecutive segments with subsumption relations. Report the threshold value combinations for each consecutive segment group.
6. Choose the threshold value combination that accommodates many stable consecutive clusters.
7. Report the consecutive segment groups under this threshold value combination.

2.3 Super Modules and Sample Groups

The modules derived from one TCGA dataset can be further simplified as there are many modules with highly correlated target expression profiles. We grouped similar clusters together by recursively incurring a variation of spectral clustering and reported Super Modules for each TCGA cancer type. Likewise, samples in the data were combined to Sample Groups with the same fashion. We briefly introduced the algorithm of generating Super Modules and Sample Groups in Methods and Supplementary Figure S11. Below we will give detailed descriptions of the algorithm.

Spectral clustering is a well-known algorithm of clustering data in Euclidean space or graphs. We treated each module as a data point and computed the mean expression profile in the samples over its member target genes. Denote the L_2 distance matrix of the mean expression profiles of modules as $\boldsymbol{U}$. $\boldsymbol{U}$ is converted into a weight matrix by $\boldsymbol{W} = e^{-\beta_W \boldsymbol{U}}$. We can view $\boldsymbol{W}$ as the weight matrix of a graph. Define $\boldsymbol{D}$ a diagonal matrix where each diagonal entry is the sum of the corresponding row entries in $\boldsymbol{W}$. The graph Laplacian of $\boldsymbol{W}$ is defined as

$$\boldsymbol{L} = \boldsymbol{D}^{-\frac{1}{2}} \cdot (\boldsymbol{D} - \boldsymbol{W}) \cdot \boldsymbol{D}^{-\frac{1}{2}}. \qquad (15)$$

The smallest eigenvalue of $\boldsymbol{L}$ is 0 and the corresponding eigenvector is $\boldsymbol{D}^{\frac{1}{2}}$. Instead we considered the second smallest eigenvalue and the corresponding eigenvector.

$$\boldsymbol{L} \cdot \boldsymbol{z}_1 = \lambda_1 \cdot \boldsymbol{z}_1. \qquad (16)$$

The spectral clustering algorithm procedures are briefly described below.

Figure X20: The spectral clustering algorithm.

Input: The distance matrix $\boldsymbol{U}$ of data points in a Euclidean space or a graph.

Output: Partition of data points into clusters.

Procedures:

1. Calculate the weight matrix $\boldsymbol{W}$ and its graph Laplacian $\boldsymbol{L}$ (equation 15).
2. Find the eigenvector $\boldsymbol{z}_1$ with the second smallest eigenvalue.
3. Binary partition the vertices in the graph according to the signs of entries in $\boldsymbol{z}_1$.
4. Recursively partition each of the two components.

To determine the free parameter β_W, we varied β_W over a range of possible values and chose the value which gave rise to the most even binary partition at the top level.

Spectral clustering outcomes give an intuitive interpretation in a graph as they are closely linked to the normalized cuts. However, like most other clustering algorithms spectral clustering does not explicitly specify the number of clusters. We augmented spectral clustering with criteria to determine the number of clusters. Here spectral clustering is used as an algorithm to sort modules. We recursively proceeded binary partitions all the way to single modules. Each partition places some modules at the left side of the dividing line and the remaining modules at the right side of the dividing line. We further considered possible sorting orders of three consecutive partitions and picked the one that minimized the distances

between adjacent clusters. After three consecutive partitions, there are four subclusters p_1, p_2, p_3, p_4, where p_1 and p_2 are grouped together and p_3 and p_4 are grouped together after the first partition. We swapped the orders of p_1 and p_2 as well as p_3 and p_4 such as the distances of adjacent subclusters were smaller than those of non-adjacent subclusters.

We proposed an algorithm to jointly cluster modules and samples according to the recursive spectral clustering outcomes. The inputs are the sorted modules and samples generated by running spectral clustering recursively. The outputs are the boundaries of the module and sample clusters in the sorted lists. The mean module expression data are sorted by the input orders of modules and samples. Conceptually, the module and sample clusters can be directly demarcated on the sorted expression data. We borrowed an algorithm in computer vision (Marr, 1982) to detect boundaries in the sorted expression data and mapped them to the sorted lists. Intuitively, we treated the mRNA expressions of sorted Modules and samples as a two-dimensional image. Boundaries of patterns in this 2D image correspond to the boundaries of Super Modules (groups of modules) and Sample Groups (groups of samples). To mitigate the interference of noise to affect boundary detection, we smoothened the image boundaries by convolving the image with Gaussian kernels. Denote $\boldsymbol{I}(x, y)$ a 2D image (the sorted expression data) and $\boldsymbol{G}_\sigma(x, y) = (\frac{1}{\sqrt{2\pi\sigma^2}})^2 \exp(\frac{-x^2}{2\sigma^2} + \frac{-y^2}{2\sigma^2})$ a Gaussian kernel with standard deviation σ. The convolution is $\boldsymbol{J}(x, y) \equiv (\boldsymbol{I} * \boldsymbol{G}_\sigma)(x, y) = \int \boldsymbol{I}(x - x', y - y')\boldsymbol{G}_\sigma(x', y')dx'dy'$. Laplacian operator ∇^2 of the smoothen image evaluates the second derivatives along each direction. $\nabla^2 \boldsymbol{J}(x, y) = \frac{\partial^2 \boldsymbol{J}(x,y)}{\partial x^2} + \frac{\partial^2 \boldsymbol{J}(x,y)}{\partial y^2}$. Thus the zero-cross points correspond to the boundaries. Furthermore, we looked for boundaries which were robust against a range of smoothening scales (kernel widths). The algorithm procedures are described below.

Figure X21: The algorithm of clustering Association Modules and samples.

Input: Sorted binary partition trees of modules and samples, sorted mean module expression data.

Output: Super Module and sample cluster boundaries.

Procedures:

1. A node in the binary partition tree specifies a number of sorted modules or samples and is called a segment.
2. Identify all segments above a given length and find their candidate boundaries.
3. Sort the expression data and generate a 2D image $\boldsymbol{I}(x, y)$.
4. Generate Gaussian kernels $\boldsymbol{G}_\sigma$ with varying widths (standard deviations σ's).
5. Convolve each Gaussian kernel to the sorted expression data to smoothen it. $\boldsymbol{J}(x, y) \equiv (\boldsymbol{I} * \boldsymbol{G}_\sigma)(x, y)$.
6. Compute Laplacian of the smoothen data. $\nabla^2 \boldsymbol{J}(x, y) = \frac{\partial^2 \boldsymbol{J}(x,y)}{\partial x^2} + \frac{\partial^2 \boldsymbol{J}(x,y)}{\partial y^2}$.
7. Find the zero-crossing points in $\nabla^2 \boldsymbol{J}(x, y)$ as the boundaries of the data.
8. Find the closest candidate boundaries relative to $\nabla^2 \boldsymbol{J}(x, y)$ boundaries.
9. Find the boundaries which are pronounced in a wide range of σ's.

We applied the algorithm to the modules of each cancer type and identified totally 217 Super Modules and 228 Sample Groups. To demonstrate that Super Modules capture important

driver alterations and functional processes of cancer, we solicit four Super Modules and illustrate their selected effectors and target genes in Supplementary Figure S4 and the paragraphs below.

Breast cancer (BRCA) Super Module 5 (Figure 4A) consists of the following prominent effectors. MYC (regulator of chr8q CNV positive association +), TP53 (+ mutation), PIK3CA and CDH1 (- mutation) are well-known driver genes (Weinberg, 2007). MNDA and MAGEB4 (- methylation) are myeloid cell differentiation antigen involved in chronic lymphocytic leukemia and other cancers (Joshi *et al*., 2007) and cancer-testis antigens associated with immunotherapy treatment responses and undergoing aberrant methylation (Almeida *et al*., 2009, Saghafinia *et al*., 2018). Mir-10a and let-7 are closely involved in various cancer-related processes (e.g., Ke and Liu, 2017, Chirshev *et al*., 2019). The target genes are highly enriched with cell cycle process.

Colon cancer (COAD) Super Module 7 (Figure 4B) consists of the following prominent effectors. SMAD2/4 (regulator of + chr18p CNV) and SFRP2 (- methylation) are members of TGF-β (Weinberg, 2007, Luo *et al*., 2019) and Wnt (Yang *et al*., 2016) pathways critical for colon cancer genesis. WT1 (- methylation) encodes a transcription factor frequently mutated in Wilms tumor and is also involved in colorectal cancers (e.g., Oji *et al*., 2003). Mir-17 (- mir) is a member of the oncomir and implicated in various malignancies (Mogilyansky and Rigoutsos, 2013). Mir-96 (- mir) promotes cell proliferation, migration and invasion in breast cancer (Hong *et al*., 2016). Phosphorylations of AKT1, ACACA and ACACB regulate growth factor responses and acetyl-CoA metabolism respectively. Furthermore, some colon cancer samples undergo hyper-mutations in a large number of

genes (Yuza *et al*., 2017). The target genes are highly enriched with immune responses, cell adhesion, and epithelial-mesenchymal transition.

Low grade glioma (LGG) Super Module 7 (Figure 4C) consists of the following prominent effectors. The positive association with chr19 43.5-43.7Mb is putatively mediated by deletions of candidate regulators such as CEBPA, NFKBIB, TGFB1, GSK3A, BCL3, RELB, AKT2, AXL. EGFR (+ mutation) and IDH1 (- mutation) are frequently mutated driver genes in lower-grade gliomas (TCGA, 2015b). GSTM1 (- methylation) detoxifies carcinogens and drugs involved in gliomas (Kilburn *et el*., 2010). Mir-9 (- mir) and mir-181 (- mir) are involved in cancers (Ma *et al*., 2010, Shi *et al*., 2008). MTOR (+ phosphorylation) is a key gene in the PI3K/AKT/mTOR pathway critical for tumorigenesis (Weinberg, 2007). The target genes are highly enriched with immune and inflammatory responses.

Liver hepatocellular carcinoma (LIHC) Super Module 2 (Figure 4D) consists of the following prominent effectors. The positive association with chr13 is putatively mediated by deletions of candidate regulators such as CDK8, FLT3, FOXO1, ELF1, RB1. CTNNB1 (+ mutation) is a key gene in the Wnt pathway and frequently mutated in gastrointestinal cancers (Weinberg, 2007). Mir-429 (- mir) suppresses tumor migration and invasion in liver cancers (Guo *et al*., 2018). Mir-15 (- mir) down-regulates BCL2 expression and thus suppresses tumor growth (Cimmino *et al*., 2005). The target genes are highly enriched with oxidation-reduction, lipid metabolism, and amino acid metabolism.

2.4 Super Module Groups and Gene Groups

Each Super Module constitutes multiple modules, and each module comprises multiple target genes. We combined the membership matrices of Super Modules to modules and modules to genes and microRNAs and generated a large but sparse membership matrix $\boldsymbol{M}$ of Super Modules to genes and microRNAs. It has 217 rows (Super Modules) and 29250 columns (genes and microRNAs). Each entry (i, j) denotes the number of modules that belong to Super Module i and contain target gene j. The Super Module membership matrix reveals two types of structures. First, certain groups of Super Modules may share many common target genes. Second, certain groups of genes may co-appear in a combination of Super Module Groups. These structures are illustrated in the heat map of the sorted Super Module membership matrix in Figure 5A, and can be viewed as factorization of the Super Module membership matrix.

We proposed an algorithm to decompose the Super Module membership matrix $\boldsymbol{M}$ into Super Module Groups and Gene Groups. In brief, we applied hierarchical clustering to the Super Modules according to their Jaccard similarities and generated a binary tree with the Super Modules on the leaves. Each node in the binary tree is the most recent common ancestor of a group of the leaves, thus can represent a group of Super Modules. We selected the nodes such that the member genes of their two children have similar enrichment patterns in six functional categories: cell cycle, immune response, cell adhesion, ribosome, respiration, and neurogenesis and projection. Those nodes are the seeds of Super Module Groups. Furthermore, we generated a binary membership matrix to report whether each gene was a consensus member among the Super Modules belonging to a Super Module Group. Unique combinatorial patterns genes are inferred from the binary membership matrix.

Figure X22: Algorithm of inferring Super Module Groups.

Input: The Super Module membership matrix $\boldsymbol{M}$ of genes and microRNAs.

Output: The Super Module Groups.

Procedures:

1. For each Super Module, identify the consensus member genes or microRNAs that appear in at least 3 member modules.
2. Extend the consensus member genes by including other genes with correlated expression profiles in the corresponding cancer types.
3. For each pair of Super Modules, count the Jaccard similarity between their extended consensus members (intersection size/union size).
4. Apply hierarchical clustering to Super Modules according to their Jaccard similarities.
5. Calculate the enrichment p-values of each Super Module in each of six key functional categories: cell cycle, immune response, cell adhesion, ribosome, respiration, and synapse.
6. Each node in the tree of hierarchical clustering represents a group of Super Modules.
7. Find the highest level nodes in the tree such that the two children have similar enrichment patterns in the key functional categories.
8. Find the highest level nodes in the tree such that the descendants of the node are not enriched with a key process, but have very similar children in term of the Jaccard similarity of Super Modules.

9. Treat the nodes retrieved from steps 7 and 8 as seeds of Super Module Groups. Attach each unlabeled Super Module to the closest Super Module Groups.

Figure X23: Algorithm of inferring Gene Groups.

Input: The Super Module membership matrix $\boldsymbol{M}$ of genes and microRNAs, Super Module Groups.

Output: The Gene Groups.

Procedures:

1. Determine whether each gene or microRNA is a consensus member of a Super Module Group by assessing the probability of its occurrence by chance.
   1.1 Suppose the Super Module Group contains Super Modules $1, \cdots, k$, and each Super Module contains $n_1, \cdots, n_k$ genes or microRNAs.
   1.2 Construct a null model that each Super Module i randomly selects n_i genes or microRNAs from N genes and microRNAs. The probability that the target gene/microRNA is selected by the Super Module is $p_i = \frac{n_i}{N}$.
   1.3 Calculate the probability that the target gene appears in $\geq m$ Super Modules according to the null model.
   1.4 The probability is $\sum_{r=m}^{k} \Pr(\text{the gene appears in } r \text{ Super Modules} | \text{null model})$.
   1.5 $\Pr(\text{the gene appears in } r \text{ Super Modules} | \text{null model}) = \sum_{c_1, \cdots, c_k : c_{1+\cdots} c_k = r} \prod_{i=1}^{k} p_i^{c_i} (1 - p_i)^{1 - c_i}$.

1.6 The target gene or microRNA is a consensus member of the Super Module Group if the p-value ≤ 0.1.

2. The result obtained from step 1 is a sparse binary matrix of Super Module Group memberships.
3. Enumerate all unique combinatorial patterns of Super Module Group memberships and count the number of genes belonging to each unique combinatorial pattern.
4. Find the unique combinatorial patterns with the highest numbers of member genes.
5. Report the member genes belonging to each unique combinatorial pattern.

The Super Module membership matrix is decomposed into 17 Super Module Groups and 18 Gene Groups.

2.5 Meta Gene Groups

The FDR-adjusted functional enrichment p-values of the 18 Gene Groups are reported in Supplementary Table S2B. The enriched functional categories and the sorted Super Module membership matrix in Figure 3A indicate that several Gene Groups are highly enriched with similar functions and possess similar Super Module memberships. We aggregated Gene Groups into three meta Gene Groups according to theses similarities. Meta Gene Group 1 consists of Gene Groups 1-3 and is highly enriched with genes involved in immune and inflammatory responses and cell adhesion. Meta Gene Group 2 consists of Gene Groups 4-6 and is highly enriched with genes involved in cell adhesion, neurogenesis, and development. Meta Gene Group 3 consists of Gene Groups 7, 8, 10, 12 and is highly enriched with genes involved in cell cycle processes.

3 Characterizing functions of IHAS

3.1 Evaluating functional enrichment of Super Modules and Gene Groups

We calculated the hyper-geometric (Fisher exact test) p-values of enrichment of 14545 MSigDB gene sets in each Super Module and each Gene Group. For each IHAS subunit (Super Module or Gene Group), the enrichment p-values are adjusted by false discovery rates using the procedures reported in Benjamini and Hochberg 1995.

3.2 Inferring recurrent effectors of Super Module Groups

A Super Module Group is essentially a collection of modules that share many common target genes across multiple cancer types. It is of importance to find the shared effectors of its module members as well. To fulfill this goal, we developed an algorithm to identify the recurrent effectors that appear frequently in a Super Module Group. The algorithm builds a null model of effector occurrence frequencies over all modules and detects the effectors whose occurrence frequencies are statistically significant according to the null model. The null model of each type of effectors is derived from the background distribution of their occurrence frequencies over all Super Module Groups. The procedures of the algorithm are described below.

Figure X24: Algorithm for identifying recurrent effectors of Super Module Groups.

Input: The effectors, regulators and targets of each Association Module, member modules of each Super Module, member Super Modules of each Super Module Group.

Output: The recurrent effectors of each Super Module Group.

Procedures:

1. Partition each chromosome into smaller windows. Count the frequencies that each window appears as a trans-acting CNV segment effector of each Super Module Group.
2. Merge the consecutive windows with identical occurrence frequencies over Super Module Groups and form CNV segments.
3. For each type of effectors and each direction of associations, obtain the background distribution of their occurrence frequencies over all Super Module Groups.
4. For each Super Module Group, report the recurrent effectors according to the following criteria.
   4.1 The occurrence frequency is statistically significant according to the background distribution of effector occurrence frequencies.
   4.2 The occurrence frequency exceeds a threshold value.
   4.3 The occurrence frequency of the opposite direction of associations is negligible.
   4.4 For trans-acting CNV segments, also require the existence of regulators which are on the segments and have statistically significant occurrence frequencies.

3.3 Building the Artery Networks spanned by explanatory paths for associations

We endowed an association between effector molecular alteration and target gene expression with a mechanistic interpretation by finding valid paths in the unified molecular interaction network connecting the effector and the target gene. Due to the large number of association pairs and extensive connectivity of the unified network, these explanatory paths cover a large portion of the unified network and thus are not quite informative about the underlying mechanisms for associations. Yet a core of the unified network is frequently traversed by the connecting paths of many association pairs, thus is indispensable for explaining many association pairs. We termed this core the *Artery Network* from the analogy of transportation or communication systems. An association pair resembles a task of transporting a unit of goods or packets from a source (effector) to a destination (target) in the unified network, which can be allocated along their valid connecting paths. All the association pairs fill the network with differential volumes of traffic. The Artery Network accommodates high volumes of the traffic. Thus transportation or communication will be severely disrupted if links in the Artery Network are severed. Finally, the Artery Networks across the 33 cancer types may share a common subnetwork responsible for explaining many association pairs in all cancer types. We termed the common subnetwork the *Consensus Artery Network*. Below we elaborate the procedures of building the Artery Network for each cancer type and the Consensus Artery Network across all cancer types. The procedures comprise three major parts. First, we computed edge weights in terms of explanatory paths for associations. Second, we identified a connected subgraph spanning the high-weight edges (and some less-weight edges) to construct an Artery Network. Third, we combined the Artery Networks from all cancer types and constructed the Consensus Artery Network accordingly.

3.3.1 Computing edge weights in terms of explanatory paths for associations

Intuitively, an edge has a high weight if it is traversed by many valid paths connecting effectors and targets. A naive definition of an edge weight is simply the number of valid connecting paths traversing the edge. This definition, however, suffers from several shortcomings. First, different (effector,target) association pairs may have very different numbers of connecting paths. Giving every path an equal weight will inflate the importance of the association pairs possessing many connecting paths. Second, even with only one association pair the connecting paths will not have equal contributions. Shorter paths are preferred since they provide simpler mechanistic explanations for the association pair (we used the same logic in prioritizing candidate effectors in terms of their shortest path lengths to the target). Third, the paths traversing highly connected hubs are less preferable as they are likely to occur by random chance. To mitigate those drawbacks, we proposed the following weighting scheme for network edges.

1. Set all edge weights to zero.
2. For each (effector,target) pair, distribute a unit mass along the connecting paths.
3. If the effector is a trans-acting segment CNV, then find all regulators and distribute a unit mass along all connecting paths of (regulator,target) pairs.
4. Distribute the unit mass of one (effector,target) pair with the following criteria.
   4.1 Start the random walk from the effector.
   4.2 Randomly jump to one downstream neighbor of the current node with an equal probability.
   4.3 Stop the random walk when reaching the target.

4.4 Denote $\pi(s,t)$ valid path connecting source (effector,s) and destination (target,t).

4.5 The probability of drifting from s to t along $\pi(s,t)$ is $Q\big(\pi(s,t)\big) = \prod_{v\,\in\pi(s,t),v\neq t}\frac{1}{d_o(v)}$, where v is a node along $\pi(s,t)$ excluding t and $d_o(v)$ its out-degree.

4.6 Conditioned on reaching t, the probability of traversing along $\pi(s,t)$ is $P\big(\pi(s,t)\big|(s,t)\big) = \frac{Q(\pi(s,t))}{\sum_{\pi'\in\Pi(s,t)}Q(\pi')}$, where $\Pi(s,t)$ denotes the collection of all valid paths connecting s and t.

4.7 Add $P\big(\pi(s,t)\big|(s,t)\big)$ to the weight of each edge along $\pi(s,t)$.

This weighting scheme fixes the aforementioned drawbacks since it (1) assigns a unit weight to each association pair, (2) path probabilities decline with path lengths, (3) paths traversing hubs with high out-degrees are assigned low probabilities. It is also computationally intractable as it has to normalize by all valid paths connecting all association pairs. This normalization step contrasts our weighting scheme with the heat kernel diffusion in networks. We proposed two alternative approximation algorithms to efficiently calculate edge weights. As noted before, the last portion of a valid path pertains to a direct interaction from a regulator (mostly a transcription factor) to the target. Thus a valid path can be decomposed into two portions from the effector to the regulator and from the regulator to the target. Since there are far more targets than regulators, a concise representation for valid paths is the product of valid paths from all effectors to all regulators and the links from all regulators to all targets. Both the former and the latter can be efficiently enumerated. The two algorithms differ by the ways to evaluate edge weights. One algorithm explicitly uses the aforementioned formula to calculate normalized path weights. The other uses a dynamic

programming formula to iteratively calculate edge weights. Both algorithms use a function enumerate_paths to enumerate valid paths.

Figure X25: Algorithm enumerate_paths for enumerating valid paths.

Input: The unified network of molecular interactions, a (source,destination) pair.

Output: Valid paths connecting the (source,destination) pair.

Procedures:

1. Start with edges emanating from the source as the candidate paths.
2. Iteratively augment the candidate paths by additional edges until the stopping criteria are satisfied.
   2.1 If a candidate path is not a valid path, then nullify it as a candidate.
   2.2 If a candidate path terminates at the destination, then add it to the list of valid paths and nullify it as a candidate.
   2.3 If no candidate paths are left, then stop.
   2.4 If the total number of candidate paths reaches 10000000, then stop.
   2.5 If the total number of valid paths reaches 1000, then stop.

Figure X26: Algorithm 1 for evaluating edge weights.

Input: The unified network of molecular interactions, an association pair (effector,target).

Output: Edge weights pertaining to the association pair.

Procedures:

1. For an (effector,target) pair, incur enumerate_paths(.) to find valid connecting paths.
2. Calculate normalized path weights according to the weighting scheme.

Figure X27: Algorithm 2 for evaluating edge weights.

Input: The unified network of molecular interactions, an association pair (effector,target).

Output: Edge weights pertaining to the association pair.

Procedures:

1. For each (effector,target) pair, incur enumerate_paths(.) to find valid connecting paths.
2. Stratify all the nodes along the valid connecting paths into *level sets* in terms of their shortest distances from the effector.
3. Denote s and t the effector and regulator, v a node in connecting paths with distance d from s, L_{d-1} the level set of nodes with distance $d-1$ from s.

4. Define $w_T(s, v)$ the sum of unnormalized path weights from s to v, and $w_B(v, t)$ the sum of unnormalized path weights from v to t.
5. $w_T(s, v)$ is iteratively calculated by $w_T(s, v) = \sum_{u \in L_{d-1}} w_T(s, u) \cdot w(u, v)$, where $w(u, v) = \frac{1}{d_u}$.
6. Likewise $w_B(v, t)$ is iteratively calculated by $w_B(v, t) = \sum_{u \in L_{d+1}} w_B(u, t) \cdot w(v, u)$.
7. For an edge (u, v), the unnormalized edge weight is $w_T(s, u) \cdot w(u, v) \cdot w_B(v, t)$. Normalize edge weights by $w_T(s, t) = w_B(s, t)$.

3.3.2 Generating the Artery Networks

The high-weight edges of the unified network constitute the Artery Network that account for the majority of the association pairs. In an analogy of transportation networks, one unit of flow from the effector to the target of an association pair is distributed along their connecting paths. The Artery Network accommodates the heaviest traffic flows in the network. We proposed a simple algorithm to extract the Artery Network from the edge weights of the whole network.

Figure X28: Algorithm for generating the Artery Network.

Input: Edge weights of the unified network of molecular interactions, association pairs between effectors and targets.

Output: The Artery Network.

Procedures:

1. Sort edges by their weights in a descending order. Extract the edges whose weights are within one-percentile and use them as the backbone of the Artery Network.
2. Denote sources as the nodes which are effectors of many association pairs but targets of few or no association pairs. Denote sinks as the nodes which are targets of many association pairs but effectors of few or no association pairs.
3. Nodes which are neither sources nor sinks in an Artery Network should not emit or absorb large net flows.
4. Therefore, add edges whose weights are below the threshold to make the intermediate nodes in the Artery Network accommodate little net flows.

The nodes in the Artery Network are the hubs that emit or absorb high volumes of traffic flows. We stratify the hub nodes into multiple levels according to their interactions. Level 1 hub nodes emit positive-weight edges to many downstream nodes but have no downstream hub neighbors. Level 2 hub nodes emit high-weight edges to level 1 hub nodes, and are not lower level hub nodes. Level 3 hub nodes emit high-weight edges to level 2 hub nodes, and are not lower level hub nodes. Higher-level hub nodes are identified similarly. The lowest and highest levels of nodes correspond to the sinks and sources of the Artery Network respectively.

The two algorithms of computing edge weights give rise to two Artery Networks. We reported the intersection of the two Artery Networks.

3.3.3 Generating the Consensus Artery Network from multiple cancer types

We identified the hub nodes and edges that appeared in at least 10 cancer types and reported the Consensus Artery Network.

4 Relating IHAS with clinical phenotypes

4.1 Aligning Sample Groups with clinical features within cancer types

For each cancer type, our clustering algorithm (Figures X20 and X21) partitions all modules into several Super Modules and all samples into several Sample Groups. TCGA provides rich molecular and clinical annotations to the samples. We aligned the Sample Groups generated by our clustering algorithm with the TCGA sample annotations and checked whether the Sample Groups were aligned with some of those predefined annotations. The alignment with patients' survival times will be discussed in the next section.

TCGA sample annotations are roughly subdivided into four categories: (1) pathology/histology classes of tumors such as stages and grades of tumors, (2) clustering outcomes from single types of data such as mRNA expressions, CNV, DNA methylation, mir expressions, and protein expressions, or from integrated data such as COC, iclusters or paradigm, (3) molecular signatures of single genes such as ER and PR status in BRCA and Kras and Braf mutations in COAD, (4) molecular signatures derived from multiple genes such as the PAM50 subtypes in BRCA, hyper-mutations and CMS in COAD, and G-CIMP

subtypes in GBM. We visualized the mRNA expressions of sorted genes and samples according to Super Modules and Sample Groups as well as the sample annotations. In addition, we calculated the concentration coefficients to measure the quality of alignments between Sample Group labels and feature values. In each Sample Group, we determined the dominant feature value that possesses the highest number of samples. A concentration coefficient is the fraction of samples whose feature values are the dominant feature values.

4.2 Prognostic analysis of Sample Groups within cancer types

Survival information is available for all cancer types of TCGA data except LAML. We counted the survival time (in days) of a patient as the interval from the date of first diagnosis and the date of reported death, and the censoring time (in days) as the interval from the date of first diagnosis and the date of last diagnosis. We quantified the associations between subunits at each level of the integrated hierarchical association structure and patients' survival times using several approaches. For each subunit (module and Super Module), we calculated the distribution of Cox regression coefficients of its member mRNA expression data, and assessed the statistical significance of survival time associations with the deviation between this distribution and a background distribution of Cox regression coefficients from all mRNA expression data. In addition, we subdivided patients into two groups according to their median expression levels over the member genes of the subunit and calculated the log rank p-values of their Kaplan-Meier (survival) curves. Within each cancer type, we also manually constructed a decision tree that related the combinatorial expression profiles of Sample Groups with their survival times.

4.2.1 Computing Cox regression coefficients and log rank p-values of Kaplan-Meier curves

Cox regression coefficients (Cox 1972) and log rank p-values of Kaplan-Meier curves (Peto and Peto, 1972) are the two most common quantitative measures in survival analysis. Denote T a random variable of death time, and the PDF of T is the hazard function:

$$\lambda(t) \equiv \lim_{\delta t \to 0^+} \frac{\Pr(t \le T \le t + \delta t)}{\delta t}. \quad (17)$$

Suppose the hazard function depends on some covariates $\boldsymbol{z} \equiv (z_1, \cdots, z_k)$:

$$\lambda(t; \boldsymbol{z}) = \lambda_0(t) \cdot e^{\boldsymbol{z} \cdot \boldsymbol{\beta}}. \quad (18)$$

$\boldsymbol{\beta} \equiv (\beta_1, \cdots, \beta_k)$ are the Cox regression coefficients specifying the associations between the covariate and the hazard function value. A high positive Cox regression coefficient denotes that a higher covariate value implies a larger hazard and thus a shorter survival time. A high negative Cox regression coefficient exhibits the opposite effect.

Cox regression coefficients can be estimated from the conditional log likelihood function. Suppose each one of the N patients possesses either death or censoring times, without loss of generality we sorted those times in an ascending order $t_1 < \cdots < t_N$, and denote d_i a binary indicator of whether t_i is a death ($d_i = 1$) or censoring ($d_i = 0$) time. Define the risk set $R(t_i)$ as the collection of patients whose death/censoring times $\ge t_i$. At the moment right before the event time t_i, patients in $R(t_i)$ still survive. For each patient $l \in R(t_i)$, the

probability of failure (death) at t_i is $\lambda(t_i; \boldsymbol{z}_l)\delta t = \lambda_0(t_i) \cdot e^{\boldsymbol{z}_l \cdot \boldsymbol{\beta}}$. Conditioned on a death event at t_i, the probability that patient i dies is:

$$\Pr(\text{patient } i \text{ dies at } t_i \mid \text{patients } \in R(t_i) \text{ survive at } t_i - \delta t) = \frac{e^{\boldsymbol{z}_i \cdot \boldsymbol{\beta}}}{\sum_{l \in R(t_i)} e^{\boldsymbol{z}_l \cdot \boldsymbol{\beta}}}. \quad (19)$$

The conditional log likelihood is:

$$\mathcal{L}(\boldsymbol{\beta}) = \sum_{\{i:\, d_i = 1\}} \boldsymbol{z}_i \cdot \boldsymbol{\beta} - \log\left(\sum_{l \in R(t_i)} e^{\boldsymbol{z}_l \cdot \boldsymbol{\beta}}\right). \quad (20)$$

Notice the index of the conditional log likelihood term i is over the patients who encounter death, while the index of the normalization term l is over the patients who encounter either death or censoring after t_i. $\boldsymbol{\beta}$ can be estimated from the death/censoring times of all patients using Newton Raphson's method.

4.2.2 Assessing statistical significance of prognostic associations for IHAS subunits

The basic subunits of the integrated hierarchical association structure within a cancer type comprise subsets of target genes with coherent expressions (e.g., Super Modules and Gene Groups). Each member gene possesses a Cox regression coefficient between its mRNA expression levels and the patients' prognosis. A subunit is related to the patients' survival times if the Cox regression coefficients of its members possess coherently large positive or negative values. To quantify this intuition, we compared two distributions of Cox regression coefficients: those drawn from the members of the designated subunit and those drawn from all genes in the data. A large deviation between the two distributions implies that the mRNA expression levels of the target genes are either negatively associated with survival times

(positive Cox regression coefficients) or the opposite. There are several standard methods to assess the deviation between empirical distributions, such as Kolmogorov-Smirnov tests and Mann-Whitney tests. Those methods, however, are very sensitive to sample sizes of empirical distributions (the numbers of genes in our case). Small deviations between the two distributions become highly significant if the sample sizes are large. This property is not desirable for our purpose since member genes are highly correlated rather than independently drawn from a distribution. The KS and MW test p-values thus over-estimate the significance of deviations. To mitigate this problem, we proposed a new measure p_{diff} to quantify the deviation of two distributions. Denote two random variables X_1 and X_2 whose PDFs are p_1 and p_2 respectively. We define p_{diff} as the difference between two probabilities that X_1 is greater and smaller than X_2:

$$p_{diff} \equiv \Pr\big(X_1 > (X_2 + \epsilon)\big) - \Pr\,(X_1 < (X_2 - \epsilon)). \qquad (21)$$

p_{diff} is superior to standard non-parametric scores due to several properties. First, a large positive or negative p_{diff} value indicates that X_1 is considerably higher or lower than X_2, compatible with the intuition about deviations. Second, p_{diff} is bounded in the interval $[-1,1]$. Third, unlike KS or MW p-values, p_{diff} is much less sensitive to the sample sizes. Fourth, p_{diff} can be efficiently computed. p_{diff} can be reduced into the difference of right and left tail probabilities of a random variable $Z \equiv X_1 - X_2, p_{diff} = \Pr(Z > \epsilon) - \Pr\,(Z < -\epsilon)$. $\Pr(Z > 0)$ and $\Pr\,(Z < 0)$ can be estimated by rejection sampling on X_1 and X_2. Given a PDF p, we want to draw N instances from the distribution p:

1. Start with an empty set $X = \phi$.
2. Repeat the following steps until $|X| = N$.

2.1 Uniformly draw a number x from the domain of p.

2.2 Evaluate $p(x)$.

2.3 Uniformly draw a number $q(x)$ from the interval $[0, p_{max}]$, where p_{max} is the maximum value of p.

2.4 If $q(x) \leq p(x)$, then $X \leftarrow X \cup \{x\}$.

p_{diff} is obtained by the fractions of instances when $X_1 > (X_2 + \epsilon)$ and $X_1 < (X_2 - \epsilon)$.

We calculated the Cox regression coefficient of each mRNA expression profile and obtained a background distribution accordingly. For each Super Module and Gene Group, we also obtained the distribution of Cox regression coefficients of its target gene expressions. The direction and significance of deviation of the two distributions was assessed by both one-side KS tests and the p_{diff} measure. We reported the KS p-values and p_{diff} scores of all Super Modules and Gene Groups for each cancer type in Supplementary Table S5.

4.2.3 Assessing statistical significance of survival function difference between subpopulations differentiated by target gene expressions

The Kaplan-Meier curve is a common non-parametric estimator of the survival function in a population of patients. The survival function $S(t) = \Pr(T > t)$ is simply the complement of the CDF of T. To estimate $S(t)$, we subdivided time by the moments of death events. The estimator is a piecewise constant function whose value remains invariant in each interval between death events. At any time t, the Kaplan-Meier curve is:

$$\hat{S}(t) = \prod_{\{i:\, t_i \leq t\}} (1 - \frac{m_i}{n_i}). \quad (22)$$

m_i and n_i denote the numbers of patients who die at time t_i and who have not died up to time t_i.

The log rank test statistic compares the estimates of the hazard functions of two or multiple groups. Suppose there are K groups. At each time t, denote N_{it} the number of group i patients who are at risk (not yet dead or being censored) at time t, and O_{it} the number of group i patients who die at time t. The null hypothesis is that the K groups have the same hazard function. To test this hypothesis, we constructed a test statistic by defining two random variables $\boldsymbol{U}$ and $\boldsymbol{V}$:

$$U_i = \sum_t [O_{it} - \sum_{l=1}^{K} O_{it} \cdot \frac{N_{it}}{\sum_{l=1}^{K} N_{lt}}].$$

$$V_{ij} = \sum_t [\sum_{l=1}^{K} O_{lt} \cdot \frac{N_{it}}{\sum_{l=1}^{K} N_{lt}} \cdot \frac{N_{jt}}{\sum_{l=1}^{K} N_{lt}} \cdot \frac{\sum_{l=1}^{K} (N_{lt} - O_{lt})}{\sum_{l=1}^{K} (N_{lt} - 1)}].$$

$$Z = \boldsymbol{U}^T \boldsymbol{V}^{-1} \boldsymbol{U}. \qquad (23)$$

Z asymptotically follows a χ^2 distribution with $K - 1$ degrees of freedom. Thus we can calculate the log rank p-value from the N_{it} and O_{it} data.

For each subunit (Super Module or Gene Group) in a cancer type, we obtained the median expression level of each patient over its target member genes, and subdivided patients into two equal sized groups according to their median expression levels. The log rank p-values of the two subgroups were reported.

4.2.4 Constructing decision trees relating combinatorial expression patterns of Sample Groups with their survival times

The aforementioned Cox regression coefficient measures and log rank p-values considered the survival association with each single subunit (Super Module or Gene Group) one time. In practice, survival times are likely affected by the combinatorial expression patterns of multiple subunits. To specify the more complex relation between survival times and Super Modules, we manually constructed decision trees relating combinatorial expression patterns of Super Modules and survival times. For each cancer type, we visualized the Kaplan-Meier curves of Sample Groups and reported their multi-group log rank p-values. The Sample Groups were aggregated according to the proximity of their Kaplan-Meier curves. Within each aggregated Sample Group, we then identified the Super Module expression patterns that were shared among its members. An example of the BLCA decision tree is illustrated in Figure X29. There are 4 Sample Groups with all very distinct Kaplan-Meier curves. Sample Groups 2 (green) and 1 (blue) have the lowest survival curves, followed by groups 4 (cyan) and 3 (red). We found expression levels of Super Module 8 best separated Sample Groups 2 and 1 (high), 4 (intermediate), and 3 (low). Between Sample Groups 2 and 1, Super Module 5 expression levels are slightly higher in the former. These delineations constitute the decision tree in the right panel of Figure X29.

In some cancer types, the combinatorial expression patterns of Super Modules are poorly associated with survival times. It is hence difficult to draw the decision trees of those cancer types. For instance, in OV the Kaplan-Meier curves of all Sample Groups are barely separable (log rank p-value 0.324). No decision tree is constructed accordingly. The

combinatorial expressions, survival curves of the Sample Groups and their decision tree of each cancer type are visualized in Supplementary Data.

4.3 Generating Pan-cancer Sample Groups

Similar to Super Modules, Sample Groups generated from multiple cancer types can be closely related and thus form a higher order structure. In brief, we solicited three Meta Gene Groups from the 18 Gene Groups: group 1 (Gene Groups 1-3) are highly enriched with immune response, group 2 (Gene Groups 4-6) are highly enriched with development and cell adhesion, and group 3 (Gene Groups 7, 8, 10, 12) are highly enriched with cell cycle. The average expression level of each large group in each sample Gene Group is quantized into a binary value (0 or 1), and there are $2^3 = 8$ combinatorial binary expressions of the three meta Gene Groups. To assign Sample Groups to Pan-cancer Sample Groups, we first derived the combinatorial expression patterns of the 228 Sample Groups over the 18 Gene Groups (Figure 4A). Certain Gene Groups are irrelevant in some Sample Groups if they are not over-represented in any Super Module of the corresponding cancer type (the white patches in Figure 4A). For instance, olfactory receptors (Gene Group 16) are target genes of Super Modules in only a few cancer types (such as GBM). They do not appear in the IHAS of other cancer types, thus are irrelevant in the corresponding Sample Groups. Furthermore, not all members of a Gene Group are relevant in a Sample Group since some members may not appear as target genes of the constituting Super Modules. We proposed an algorithm to select members of the Gene Groups over-represented in the Super Modules pertaining to each Sample Group. The combinatorial expression of a Gene Group in a Sample Group is the average expression of the selected genes among the Sample Group members (a patch in Figure 6A), and the average expression value is not valid if no genes are selected (white

patches in Figure 6A). We then quantized the combinatorial expression patterns into binary values and assigned the binary Meta Gene Group states of each Sample Group accordingly. The 8 Pan-cancer Sample Groups immediately arise from the 3-bit Meta Gene Group states. More precise procedures are described below.

Figure X29: In BLCA data, the combinatorial expression patterns of Super Modules are displayed (left panel). Patients are subdivided into 5 groups in terms of Sample Groups, and their Kaplan-Meier curves are displayed (middle panel). The distinction of the Kaplan-Meier curves in these groups is explained by a decision tree in terms of the combinatorial expression patterns of Super Modules.

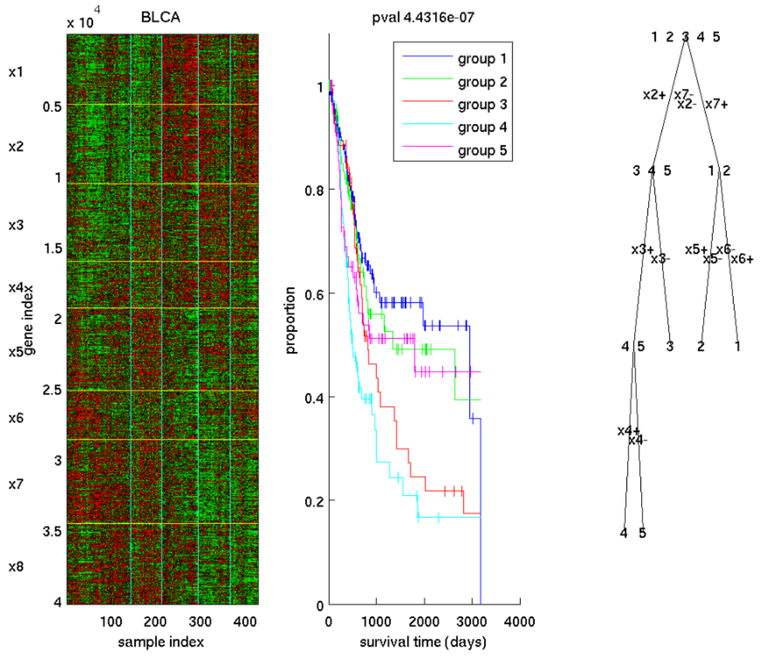

Figure X30: Algorithm for generating Pan-cancer Sample Groups.

Input: Super Modules, Gene Groups, and Sample Groups, mRNA expression data of all cancer types.

Output: Assignments of Sample Groups to one of the 8 combinatorial expression patterns of three large groups.

Procedures:

1. Subdivide the mRNA expression data of each cancer type into grids of spanned by Super Modules and Sample Groups. Calculate the mean expression value for each entry (Super Module-Sample Group combination) of the grids. Denote this grid expression matrix as A.
2. For each Super Module, consider the representative Gene Groups with enrichment p-value 10^{-10}.
3. For each Gene Group, calculate the partial expression profile over the Super Module-Sample Group grids. If the Gene Group is over-represented in a Super Module, then extract the intersection of the Gene Group members and Super Module members and calculate their mean expression data in each Sample Group. If the Gene Group is not over-represented in a Super Module, then place an invalid (NaN) value to in the entries corresponding to the Super Module in the grids. Denote the grid expression matrix of Gene Group l as B_l.
4. For each Super Module l, compare A and B_l and report whether each entry has compatible expressions (meaning that the expression values are either close or the quantized expression values have the same signs).

5. Construct a binary selection matrix C where C_{ij} denotes that Gene Group j is selected in Super Module i. The following criteria are adopted.

   5.1 Each row and each column in C contain at least one unit entry. In other words, at least one Gene Group is selected for each Super Module, and at least one Super Module contains each Gene Group. If not possible, then minimize the number of rows and columns with all zero entries.

   5.2 If $C_{ij} = 1$, then ensure the ith row in A and the jth row in B_l are inconsistent in at most 2 Sample Groups.

6. For each cancer type, select genes according to C. For each $C_{ij} = 1$, find the intersection of Super Module i and Gene Group j members, and take the union of those genes.

7. For each Sample Group, find the corresponding cancer type and subdivide the selected genes into Gene Groups. Calculate the average expression levels of intersections between selected genes and each Gene Group.

8. For each Sample Group, quantize the average expression levels of Gene Groups into a binary vector q.

9. Collapse the quantized binary vector q into a three-component binary vector r. $r_1 = 1$ if $(q_1 = 1) \vee (q_2 = 1) \vee (q_3 = 1), r_2 = 1$ if $(q_4 = 1) \vee (q_5 = 1) \vee (q_6 = 1), r_3 = 1$ if $(q_7 = 1) \vee (q_8 = 1) \vee (q_{10} = 1) \vee (q_{12} = 1)$.

10. Assign r to one of the 8 combinatorial expression patterns.

4.4 Aligning Pan-cancer Sample Groups with pan-cancer phenotypes

In addition to phenotypes of individual cancer types, TCGA also provides information about the phenotypes that appear across multiple cancer types. We aligned the 8 Pan-cancer Sample Groups with four pan-cancer phenotypes: hypermutations of selected samples, mesenchymal subtypes, purity levels of samples (Aran *et al*., 2015), and stemness of samples (Malta *et al*., 2018). Annotations of these pan-cancer phenotypes are juxtaposed with the sorted mRNA expression data of all Gene Groups and all samples in the TCGA data (Figure 6B), and the sorted mRNA expression data of the three meta Gene Groups in 7 cancer types (Figure 6C).

4.5 Integrating effector and target information within and across cancer types

To obtain a holistic view about various aspects of IHAS information, we integrated the functional enrichment of Super Modules, occurrence of effectors, combinatorial mRNA expression patterns of Super Modules and Sample Groups, alignment of clinical phenotypes with Sample Groups, and survival information in each Sample Group in 8 selected cancer types and visualized them in Supplementary Figures S10-11. Since the numbers of enriched functional categories, effectors and clinical phenotypes are too large to visualize, we solicited them by the following criteria. For functional categories, we selected the MSigDB Gene Sets which are either annotated with HALLMARK or involved in neuron functions, and calculated their FDR-adjusted hyper-geometric enrichment p-values in each Super Module. For effectors, we chose the hub genes in the Consensus Artery Network which were effectors, and counted their occurrences in the positive and negative association directions. For clinical phenotypes, we manually selected one or two phenotypes from Table 5, and calculated the phenotype value composition in each Sample Group. For survival information,

we counted the patients in each Sample Group with survival or censoring times and the patients whose survival or censoring times surpassed 5 years (1800 days). Below we summarize the integrated information of 8 cancer types.

Figure S10A displays the integrated information of ACC. Super Modules 1 and 3 are enriched with cell cycle control and immune response respectively. Prominent positive effectors include EIIF4EBP1 and RPS6 for Super Module 1 and STAT3 for Super Module 3. Sample Group 1 has high expression in Super Module 3 and moderate-low expression in Super Module 1. Sample Group 2 has high expression in Super Module 1 and low expression in Super Module 3. Sample Group 3 has low expression in Super Modules 2 and 3. Sample Group 1 is dominated by the mRNA phenotype of "steroid phenotype low" and the methylation phenotype of "CIMP-low". Sample Group 2 is dominated by the mRNA phenotype of "steroid phenotype high" and the methylation phenotype of "CIMP-intermediate". Sample Group 3 is dominated by the mRNA phenotype of "steroid phenotype high + proliferation" and the methylation phenotype of "CIMP-high". Sample Group 1 comprises a higher proportion of long-surviving patients than other Sample Groups.

Figure S10B displays the integrated information of BRCA. Super Modules 1-3 are enriched with estrogen response, and Super Module 1 is also enriched with neuron functions. Super Modules 4-5 are enriched with cell cycle control. Super Module 6 is enriched with cell cycle control, immune response, development, and neuron function. Super Modules 7-8 are enriched with immune response, development, and neuron function. Prominent positive effectors include BRCA1, NF1, STAT5B for Super Modules 2-3, EIIF4EBP1 and SHC1for Super Modules 4, FOXM1, TEAD4, SETDB1, GAPDH, ATF7IP for Super Modules 5-7, MAPK14, FOXO3, FYN, HDAC2 for Super Modules 6-7, and MTOR, RUNX3, RPS6KA1

for Super Modules 7-8. Sample Group 1 has high expression in Super Modules 7-8, moderate-high expression in Super Module 1, and low expression in Super Modules 3-5. Sample Group 2 has moderate-high expression in Super Modules 1-2 and low expression in Super Module 5. Sample Group 3 has high expression in Super Modules 5-7 and low expressions in Super Modules 1-3 and 8. Sample Group 4 has high expression in Super Modules 5-6 and low expression in Super Modules 1-2 and 8. Sample Group 5 has high expression in Super Modules 3-4 and low expression in Super Modules 6-8. Sample Group 6 has high expression in Super Modules 2-3 and low expression in Super Modules 5-8. Sample Groups 1-2 are dominated by luminal A tumors. Sample Group 3 is dominated by basal-like tumors. Sample Group 4 is a mixture of Her2-enriched and luminal B tumors. Sample Groups 5-6 are mixtures of luminal A and luminal B tumors. Sample Groups 1-2 comprise slightly higher fractions of long-surviving patients than Sample Groups 4-5.

Figure S10C displays the integrated information of COAD. Super Module 1 is enriched with neuron function. Super Modules 3 and 8-10 are enriched with cell cycle control. Super Module 4 is enriched with ribosome and respiration. Super Module 6 is enriched with immune response and development. Super Module 7 is enriched with neuron function, immune response and development. Super Modules 8 and 10 are enriched with cell cycle control. Super Module 8 is enriched with immune response and cell cycle control. Prominent positive effectors include MAPK3, CDH1, CBERBP for Super Modules 1 and 7, NF1, CDK5, PDX1, NLK, MED1, STAT5B for Super Modules 3-4, PLOR2A, SMAD4, EP300, SREBF1 for Super Modules 7-8, SMAD3 and E2F1 for Super Module 6. Sample Group 1 has high expression in Super Modules 6-7 and moderate-high expression in Super Modules 8-10, and low expression in Super Modules 2-4. Sample Group 2 has moderate-high expression in Super Modules 8 and 10 and low expression in Super Modules 1, 5, 7.

Sample Group 3 has high expression in Super Modules 1-3 and low expression in Super Modules 6-7 and 9-10. Sample Group 4 has high expression in Super Modules 3-5 and low expression in Super Modules 6-9. Sample Group 5 has high expression in Super Modules 1 and 7 and low expression in Super Modules 3, 8 and 10. Sample Group 1 is a mixture of CMS1 and CMS4 tumors and dominated by MSI/CIMP mRNA subtypes. Sample Group 2 has diverse CMS tumors and a mixture of invasive and MSI/CIMP mRNA subtypes. Sample Groups 3-4 are dominated by CMS tumors and CIN mRNA subtype. Sample Group 5 is dominated by CMS4 tumors a mixture of mRNA subtypes. Sample Groups 2 and 4 comprise slightly higher fractions of long-surviving patients than others.

Figure S10D displays the integrated information of ESCA. Super Module 1 is enriched with lipid metabolism. Super Modules 2 and 4 are enriched with neuron function. Super Modules 3 and 6 are enriched with cell cycle control. Super Module 5 is enriched with neuron function and cell cycle control. Prominent positive effectors include PRKCD and PCM1 for Super Modules 1-2, SRC for Super Module 1, GSK3B, PRKAA1, MITF for Super Modules 2 and 4, ZNF148, TBL1XR1, MAPK1 for Super Modules 4-5, KDM5B and RBBP5 for Super Modules 4 and 6, and FOXM1, TEAD4, GAPDH for Super Modules 5-6. Sample Group 1 has high expression in Super Modules 4-5 and low expressions in Super Modules 1-3. Sample Group 2 has high expression in Super Modules 1, 2, 4 and low expression in Super Modules 5-6. Sample Group 3 has high expression in Super Modules 1 and 3 and low expression in Super Modules 4-5. Sample Group 1 is dominated by esophagus squamous cell carcinoma. Sample Groups 2-3 are dominated by esophagus adenocarcinoma NOS.

Figure S11A displays the integrated information of LGG. Super Modules 1-3 are enriched with neuron function. Super Modules 4-5 are enriched with cell cycle control. Super Module

6 is enriched with cell cycle control, immune response, and development. Super Module 7 is enriched with immune response, and development. Prominent positive effectors include RB1 for Super Modules 1-2, MAPK8 and EIF4EBP1 for Super Modules 1 and 3, FOXO3 and PRKCA for Super Module 2, MTOR, TGFB1, GSK3A, and BCL3 for Super Modules 6-7. Sample Group 1 has high expression in Super Modules 5-7 and low expression in Super Modules 1-4. Sample Group 2 has low expression in Super Module 1. Sample Group 3 has high expression in Super Modules 4-5 and low expression in Super Modules 1-2. Sample Group 4 has high expression in Super Modules 3-4 and low expression in Super Module 6-7. Sample Group 5 has high expression in Super Modules 1-2 and low expression in Super Modules 4-6. Sample Group 1 is dominated by IDH wild type. Sample Groups 2-3 are dominated by IDH mutant-non codel subtype. Sample Group 4 is dominated by IDH mutant-codel subtype. Sample Group 5 is a mixture of IDH mutant codel and IDH mutant noncodel subtypes.

Figure S11B displays the integrated information of LUSC. Super Module 1 is enriched with immune response, development, and neuron function. Super Module 4 is enriched with cell cycle control. Prominent positive effectors include JUN and NFKB1for Super Module 1, MTOR, PRKCA, FBXW7, JAK2, MAPK1 for Super Modules 1 and 4, EGFR, MET, AKT1, ASH2L, PRKDC, TCEA1, E2F1, EIF6, EP300 for Super Modules 3-4. Sample Group 1 has high expression in Super Modules 1-2 and low expression in Super Modules 3-4. Sample Group 2 has moderate-high expression in Super Module 1 and low expression in Super Module 4. Sample Group 3 has low expression in Super Modules 1-2. Sample Group 4 has high expression in Super Module 4 and low expression in Super Modules 1-2. Sample Group 1 is dominated by secretory tumors. Sample Group 2 is a mixture of secretory and basal

tumors. Sample Group 3 is a mixture of four subtypes. Sample Group 4 is dominated by classical tumors.

Figure S11C displays the integrated information of PCPG. Super Module 1 is enriched with RNA splicing and respiration. Super Module 3 is enriched with immune response and development. Super Modules 4-5 are enriched with neuron function. Super Modules 6-7 are enriched with protein secretion/localization. Prominent positive effectors include FOXO3 and RAF1 for Super Module 3, TRAM1, MTOR, SKIL, NF1 for Super Modules 3 and 6, ATF2 and STAT1 for Super Modules 6 and 7. Sample Group 1 has high expression in Super Modules 1 and 3 and low expressions in Super Modules 4-7. Sample Group 2 has moderate-high expression in Super Module 7 and low expression in Super Modules 1 and 3. Sample Group 3 has high expression in Super Modules 4-5 and 7 and low expression in Super Modules 1 and 3. Sample Group 1 is dominated by the pseudohypoxia mRNA subtype and intermediate methylated subtype. Sample Groups 2-3 are dominated by kinase signaling mRNA subtype and low methylated subtype.

Figure S11D displays the integrated information of SARC. Super Module 1 is enriched with cytoskeleton, myogenesis, and neuron function. Super Modules 4-6 are enriched with cell cycle control and development. Super Modules 7-8 are enriched with immune response and development. Prominent positive effectors include AKT2, GSK3A, CDKN2A for Super Modules 1 and 5, EIF4EBP1, SETDB1, RIT1, UBQLN4 for Super Modules 4-5, CDK5, SMARCD1, ATF1, SP1, CDK4, MDM2 for Super Modules 7-8. Sample Group 1 has high expression in Super Modules 1-2 and 4 and low expression in Super Modules 5-8. Sample Group 2 has low expression in Super Module 4. Sample Group 3 has high expression of Super Module 5 and low expression in Super Modules 1-2. Sample Group 4 has high

expression of Super Modules 5, 7-8 and low expression in Super Modules 1-4. Sample Group 1 is dominated by STLMS histology subtype. Sample Group 2 is dominated by DDLPS subtype. Sample Groups 3-4 are dominated by UPS subtype.

We also combined the information of effectors and targets of all Super Modules across all cancer types to identify the pathways that impact distinct Gene Groups or Meta Gene Groups. First, we restricted effectors to the 379 hub genes from the Consensus Artery Network. Second, for each hub effector we counted its occurrence in each Super Module Group. Positive and negative associations in a Super Module Group were counted separately. Third, for each hub effector we determined whether it appeared frequently in a Super Module Group according to two criteria: (1) the occurrence in one direction ≥ 6, (2) the occurrence in this direction ≥ 3 folds of the occurrence in the other direction. Using these criteria we quantized the occurrence of each hub effector over Super Module Groups into a trinary vector: +1 and -1 denote that the hub effector occurs frequently in positive and negative directions of a Super Module Group. Fourth, we selected 101 hub effectors which occurred frequently in at least one Super Module Group and sorted their quantized occurrence vectors. Fifth, we selected 35 MSigDB Gene Sets which contained ≥ 3 selected hub effectors and were not generated from high-throughput data. All of those 35 Gene Sets happen to be pathways. Sixth, we sorted the 35 pathways according to their membership vectors of the 101 selected hub effectors.

According to the selected hub effectors and pathways we constructed 4 matrices/tensors. M_1 (17×18) is the binary over-representation matrix of 18 Gene Groups in 17 Super Module Groups. M_2 $(17\times101\times2)$ is the occurrence tensor of 101 selected hub effectors in 17 Super Module Groups and 2 directions. M_3 (35×101) is the membership matrix of 101 selected

hub effectors in 35 pathways. M_4 ($35 \times 18 \times 2$) is the aggregate tensor combining M_1-M_3: $M_4(:,:,i) = M_1 \cdot M_2(:,:,i) \cdot M_3$. Furthermore, we identified and marked 16 selected hub effectors which appeared in ≥ 5 selected pathways. They include the following genes: CDKN2A, RB1, CDK2, TP53, E2F1, AKT1, PRKCA, SHC1, RAF1, STAT3, MAPK1, SRC, MAPK3, MAPK8, MAPK14, EGFR.

By inspecting the visualization of M_4 we subdivided the 35 pathways into two groups. Group 1 includes the following 14 pathways: BIOCARTA RACCYCD, BIOCARTA ATM, BIOCARTA G1, BIOCARTA G2, BIOCARTA CTCF, BIOCARTA CELL CYCLE, BIOCARTA P53, BIOCARTA RB, BIOCARTA TEL, BIOCARTA ARF, REACTOME G0 and early G1, REACTOME YAP1 and WWTR1 TAZ STIMULATED GENE EXPRESSION, REACTOME G1 PHASE, REACTOME E2F MEDIATED REGULATION OF DNA REPLICATION. Group 2 includes the following 21 pathways: REACTOME GAB1 SIGNALOSOME, BIOCARTA EDG1 PATHWAY, BIOCARTA EIF4 PATHWAY, BIOCARTA BIOPEPTIDE PATHWAY, BIOCARTA ATR1 PATHWAY, BIOCARTA ECM PATHWAY, BIOCARTA INTEGRIN PATHWAY, BIOCARTA PYK2 PATHWAY, BIOCARTA HER2 PATHWAY, BIOCARTA SPRY PATHWAY, REACTOME SIGNALING TO RAS, REACTOME SIGNALING TO ERKS, KEGG TYPE II DIABETES MELLITUS, BIOCARTA AGR PATHWAY, BIOCARTA SPPA PATHWAY, BIOCARTA ERK PATHWAY, BIOCARTA MPR PATHWAY, BIOCARTA VARRESTIN SRC PATHWAY, BIOCARTA MET PATHWAY, REACTION GROWTH HORMONE RECEPTOR SIGNALING, REACTOME NEGATIVE REGULATION OF FGFR SIGNALING. The first 5 selected hub effectors (from CDKN2A to E2F1) frequently occur on pathway group 1, and the remaining 11 selected hub effectors (from AKT1 to EGFR) frequently occur on pathway group 2.

5 Validating IHAS on external datasets

We validated the integrated hierarchical association structures in and their relations with clinical phenotypes in various external datasets from both tumors and normal tissues. Validation focuses on several aspects of the integrated hierarchical association structures: (1) whether the subunits (modules, Super Modules, Gene Groups, etc.) retain coherent expressions in external data, (2) whether the associations between effector molecular alterations and target gene expressions are preserved in external data, (3) whether the relations between target gene expressions of subunits and prognosis are preserved in external data, (4) whether the combinatorial expression patterns of Super Modules can be reproduced in external data, (5) whether the gene sets derived from TCGA possess functional implications in the datasets of drug responses and gene dependencies in cancer cell lines, (6) whether the gene sets derived from TCGA possess functional implications in the datasets of transcriptomes and epigenomes of normal tissues.

5.1 Juxtaposition of cancer subtypes and combinatorial expression patterns of Super Modules

Subtypes of breast cancers and glioblastomas are demarcated according to the expression signatures of selected genes. To demonstrate the persistent relations between Super Module gene expression patterns and cancer subtypes, we visualized the transcriptomic expressions of TCGA BRCA and GBM data as well as METABRIC and REMBRANDT data, and

juxtaposed the cancer subtypes of samples. For TCGA data, genes and samples are sorted according to the clustering outcomes, while the boundaries of Super Modules and Sample Groups are demarcated. For external data, genes are sorted with the same order and Super Module boundaries are identical to TCGA, while samples are sorted by subtypes that give rise to similar expression patterns as TCGA.

5.2 Evaluating expression coherence of Super Modules and Gene Groups

An immediate validation of IHAS is to check whether the genes in each Super Module or Gene Group retain coherent expressions on external data. We quantified the expression coherence of a collection of genes with two scores: the median correlation coefficient among the expression profiles of the member gene pairs and the p_{diff} deviation between the correlation coefficient distribution of the member gene pairs and the background distribution of all gene pairs in the data. To reduce the burden of assessing the background distribution we randomly selected 5000 genes and calculated their distribution of pairwise correlation coefficients excluding self-correlations. Expression coherence is reported in all external datasets.

5.3 Associations between association structure and survival outcomes

METABRIC, REMBRANDT, and a subset of the GEO datasets provide prognostic information of survival and censoring times. Verification of prognostic information on external data is similar to the prognostic analysis on TCGA data. For each Super Module and Gene Group, we calculated the deviation score p_{diff} of Cox regression coefficients distribution relative to the background distribution. For METABRIC and REMBRANDT

data, we grouped patients by their expression subtypes, visualized their Kaplan-Meier curves, and reported their log rank p-values.

5.4 Aligning the combinatorial expression patterns in the GEO datasets

For each cancer type in TCGA, the combinatorial expression patterns denote the average expression levels of each combination of Super Module and Sample Group. We anticipated that the whole or parts of those combinatorial expression patterns are reproducible in the external datasets of the same cancer type. To identify similar or partially similar combinatorial expression patterns in external datasets, we need to align Super Modules and Sample Groups in the TCGA data with the counterparts in an external data. Alignments of Super Modules are immediate since they have to share the same groups of genes. Alignments of Sample Groups are less trivial since samples in TCGA and external data are not directly related. We aligned Sample Groups in both TCGA and external datasets and generated the combinatorial expression patterns in the external dataset. In brief, we employed spectral clustering recursively to both datasets and generated two trees of binary partitions of samples, and developed a dynamic programming algorithm to align the binary partition trees and generate the combinatorial expression patterns in the external data. A valid alignment has to respect the tree structures. If nodes A and B are aligned, then descendants of A are aligned with B or its descendants and vice versa. Furthermore, the loss function of a valid alignment is defined recursively by the sum of loss functions of the best sub-alignments among the children of the current nodes and the match/gap score of aligning the current nodes. A belief propagation like method returns the globally optimal assignment. More precisely, the algorithm consists of two parts: recursive evaluation of the loss function

value for each pair of nodes between the two binary partition trees, and recursive determination of the alignment that minimizes the loss function.

Figure X31: Algorithm of recursive_loss_evaluation: recursively evaluating the loss functions.

Input: Two binary partition trees of samples T_1 and T_2 from the two datasets, their gene expression data, the current nodes $v_1 \in T_1$ and $v_2 \in T_2$.

Output: loss functions $L_1(:,:)$ and $L_2(:,:)$ of pair v_1 and v_2 and their descendants.

Procedures:

1. Define a patch p_1 of v_1 as a K-component vector, where each component is the average expression level over the Super Module target members and samples encompassed by v_1. Define a patch p_2 of v_2 likewise.
2. If both v_1 and v_2 are leaf nodes, then $S_1(v_1, v_2)$ is the number of mismatched components between p_1 and p_2 (one component has a value ≥ 0.55 and another has a value ≤ 0.45), and $S_2(v_1, v_2)$ is the Euclidean distance between p_1 and p_2. $L_1(v_1, v_2) = S_1(v_1, v_2), L_2(v_1, v_2) = S_2(v_1, v_2)$. Stop.
3. If v_1 is not a leaf node and v_2 is a leaf node, then $L_1(v_1, v_2) = S_1(v_1, v_2) + 0.4 \cdot (\text{\# Super Modules}), L_2(v_1, v_2) = S_2(v_1, v_2) + \sqrt{\frac{1}{9} \cdot (\text{\# Super Modules})}$. Stop.

4. If v_1 is a leaf node and v_2 is not a leaf node, and suppose c_1 and c_2 are two children of v_2. Then $L_1(v_1, v_2) = S_1(v_1, v_2) + \min(L_1(v_1, c_1), L_1(v_1, c_2))$, $L_2(v_1, v_2) = S_2(v_1, v_2) + \min(L_2(v_1, c_1), L_2(v_1, c_2))$. Denote c the matched child of v_2. Set the current nodes to v_1 and c and incur recursive_loss_evaluation.
5. If neither v_1 nor v_2 is a leaf node, then consider all possible alignments between the trios of v_1 and v_2. The loss functions are the infinimum from all those possible alignments.

   5.1 Both children of v_1 are aligned with both children of v_2. The loss functions $L_1(v_1, v_2)$ and $L_2(v_1, v_2)$ are the sum of loss functions for children's alignments plus $S_1(v_1, v_2)$ and $S_2(v_1, v_2)$ respectively. Spin off two current node pairs corresponding to the matched children pairs and incur recursive_loss_evaluation.

   5.2 $v_1$is aligned to a child c_1 of v_2. The loss functions $L_1(v_1, v_2)$ and $L_2(v_1, v_2)$ are the loss functions of aligning $v_1$and c_1, plus the gap penalty of not aligning c_2, another child of v_2, to any node in T_1. The gap penalties are $0.4 \cdot (\text{\# Super Modules})$ and $\sqrt{\frac{1}{9} \cdot (\text{\# Super Modules})}$ for the two loss functions. Set the current nodes to v_1 and c_1 and incur recursive_loss_evaluation.

   5.3 A child c_1 of v_1 is aligned to v_2. The loss functions can be computed in a reciprocal manner. Move the current nodes to c_1 and v_2 and incur recursive_loss_evaluation.

   5.4 A child c_1 of v_1 is aligned to a child c_2 of v_2, and other children of v_1 and v_2 are not aligned. The loss functions are the loss functions of aligning c_1 and c_2 plus the gap penalty of not aligning the other children of v_1 and v_2. Set the current nodes to c_1 and c_2 and incur recursive_loss_evaluation.

Figure X32: Algorithm of recursive_alignment_determination: recursively aligning nodes in the binary partition trees.

Input: Two binary partition trees of samples T_1 and T_2 from the two datasets, their gene expression data, loss functions $L_1(:,:)$ and $L_2(:,:)$ for all node pairs, the current nodes $v_1 \in T_1$ and $v_2 \in T_2$.

Output: Alignment mappings f_{12} and f_{21} that map v_1 and v_2 to the counterparts in T_2 and T_1 respectively. One node can be mapped to one or multiple nodes.

Procedures:

1. If both v_1 and v_2 are leaf nodes, then $f_{12}(v_1) = v_2$ and $f_{21}(v_2) = v_1$. Stop.
2. If v_1 is not a leaf node and v_2 is a leaf node, then $f_{12}(v_1) = v_2$ and $f_{21}(v_2) = v_1$. Stop.
3. Suppose v_1 is a leaf node and v_2 is not a leaf node. Suppose c_1 and c_2 are two children of v_2, and $L_1(v_1, c_1) < L_1(v_1, c_2)$. Then $f_{12}(v_1) = (v_2, c_1), f_{21}(v_2) = v_1, f_{21}(c_1) = v_1$. Suppose $L_1(v_1, c_1) = L_1(v_1, c_2)$ but $L_2(v_1, c_1) = L_2(v_1, c_2)$, then $f_{12}(v_1) = (v_2, c_1), f_{21}(v_2) = v_1, f_{21}(c_1) = v_1$. Move the current nodes to v_1 and c_1 and incur recursive_alignment_determination.
4. If neither v_1 nor v_2 is a leaf node, then find the alignment of the trios of v_1 and its children and of v_2 and its children that minimizes the loss function $L_1(:,:)$. If there are multiple minimizers for $L_1(:,:)$, then find the alignment that minimizes $L_2(:,:)$.

Set f_{12} and f_{21} according to the best alignments. Spin off and move current nodes according to the best alignment. Incur recursive_alignment_determination.

The function building_alignments establishes alignment mappings between the two partition trees by employing recursive_loss_evaluation and recursive_alignment_determination in sequence.

Figure X33: Algorithm of building_alignment: build alignments of the two binary partition trees.

Input: Two binary partition trees of samples T_1 and T_2 from the two datasets, their gene expression data.

Output: Alignment mappings f_{12} and f_{21} for all nodes in T_1 and T_2 respectively.

Procedures:

1. Initialize $L_1(:,:) = 0$ and $L_2(:,:) = 0$, and the current nodes to the roots of T_1 and T_2 respectively.
2. Incur recursive_loss_evaluation recursively to calculate the loss function values $L_1(:,:)$ and $L_2(:,:)$ for all pairs of nodes.
3. Set the current nodes to the roots of T_1 and T_2 respectively.
4. Incur recursive_alignment_determination recursively to determine the alignment mappings f_{12} and f_{21}.

5.5 Assessing preservation of CNV-mRNA associations

CNV data are available for two external datasets of specific cancer types (METABRIC and REMBRANDT) and one external dataset of multiple cancer types (CCLE). We verified CNV-mRNA associations of these two types of external data with slightly different approaches. For specific cancer types, we extracted the effectors and targets of trans-acting CNV modules in TCGA BRCA and GBM data assessed their association strength in METABRIC and REMBRANDT data by (1) median correlation coefficients between effectors and targets and (2) their p_{diff} scores. For multiple cancer types, we extracted the recurrent effectors of CNV segments from TCGA data for each Super Module Group, and identified the corresponding enriched Gene Groups. We then evaluated the association strength between the CCLE CNV data of the recurrent effectors and the CCLE mRNA expression data of the members of the enriched Gene Groups using both median correlation coefficients and p_{diff} scores.

5.6 Assessing preservation of other types of effector-target associations

CCLE possesses all types of TCGA molecular alteration data beyond CNV (mutations, DNA methylations, etc.). These data cover many effector genes, but relatively few of them are recurrent by our definition. To better validate those effector-target associations, we asked whether the numbers of effector-target associations of effector genes in TCGA were positively correlated with their numbers of effector-target associations in CCLE. Specifically, we sorted effector genes (mutated genes, methylated genes, etc) by their numbers of effector-target associations in TCGA and grouped them into bins of 10 genes. In

each bin, we then extracted the corresponding effector-target associations in CCLE and counted the associations with compatible and incompatible directions relative to TCGA. One type of effector-target associations is preserved in CCLE if the number of compatible associations in CCLE declines with the rank of the effector bin in terms of the number of associations in TCGA, while the number of incompatible associations is relatively invariant with the rank. We assessed preservation of effector-target associations for four types of effectors in CCLE data: mutations, DNA methylations, microRNA expressions, and protein phosphorylations. The results are summarized in Supplementary Figure S8.

5.7 Relating Gene Group expressions and drug response data

One external dataset comprises drug response data. CCLE reports the IC_{50} values of 24 drugs on 1046 cancer cell lines. We related IHAS from TCGA with the two drug response data by different means. In CCLE, for each (compound,gene) pair we evaluated the correlation coefficient between the IC_{50} values of the compound and the mRNA expression values of the gene over the 1046 samples. A negative correlation denotes that cells with high expression values of the gene are also sensitive to the drug treatment (low IC_{50} values), and a positive correlation denotes the opposite relation. To verify whether the expressions of Gene Groups are indicative about drug responses, we determined the directions and calculated the p_{diff} scores of all (compound,Gene Group) pairs and.

5.8 Relating Gene Group expressions and gene dependencies

Gene dependency data are not provided in TCGA. To use them to verify the integrated hierarchical association structure in TCGA, we examined whether the correlations between Achilles gene dependency data and CCLE mRNA expression data of Gene Groups possessed certain patterns. We calculated the correlation coefficient between each pair of (growth response,mRNA expression) profiles from the Achilles gene dependency data and CCLE mRNA expression data. Furthermore, for each pair of (perturbed gene,Gene Group) we computed the average correlation coefficient over target members of the Gene Group. Since gene dependency data were generated by two technologies (RNAi and CRISPR), we also selected the perturbed genes whose dependency data between RNAi and CRISPR technologies were correlated, and whose (perturbed gene,Gene Group) correlation matrices between RNAi and CRISPR technologies were also correlated. 2000 perturbed genes were selected accordingly. Figures 9C-D display the (gene dependency data, Gene Group expression data) correlation coefficients generated by RNAi and CRISPR technologies. The RNAi and CRISPR correlation matrices are highly similar. We subdivided the 2000 perturbed genes into 3 stable clusters. Cluster 1 (939 genes) possess moderate positive correlations with Gene Groups 7, 10, 12, 13, 14, 15 and moderate negative correlations with Gene Groups 1, 2, 6, 11. Cluster 2 (675 genes) possess moderate negative correlations with Gene Groups 7, 10, 12, 13, 14, 15 and moderate positive correlations with Gene Groups 1, 3, 6. Cluster 3 (386 genes) possess moderate/weak negative correlations with Gene Groups 7, 10, 12 and positive correlations with Gene Groups 1, 2, 11. A negative association between a perturbed gene and a Gene Group denotes that cell lines possessing high expressions of the Gene Group undergo more growth reduction upon the deletion of the perturbed gene. A positive association implies the opposite relation. Thus, deleting genes in cluster 1 will reduce growth of cell lines with higher cell differentiation/immune response activities but will elevate growth of cell lines with higher cell proliferation/division activities. In contrast,

deleting genes in cluster 2 will will reduce growth of highly proliferative/dividing cell lines but will elevate growth of quiescent/metastasizing/differentiating cell lines. Deleting genes in cluster 3 induces very similar responses to those of cluster 2.

We calculated the hypergeometric enrichment p-values of MSigDB gene sets for each cluster and reported the outcomes in Supplementary Table S10I. Curiously, certain functional classes are either uniquely enriched in one gene clusters or commonly enriched in multiple clusters. Gene sets of cell adhesion, cell-cell communication, immune response, cell development and differentiation are enriched in cluster 1. Gene sets of respiration, splicing, protein complex disassembly, and translation are highly enriched in cluster 2. In contrast, various gene sets related to cell cycle/proliferation/division are enriched in clusters 1, 2, 3 or their combinations.

5.9 Verifying the impacts of selected hub effectors in gene dependency data

From the integrated data of effectors and targets of Super Module Groups we identified 16 selected hub genes which occurred frequently in some Super Module Groups and appeared in $\geq$ 5 selected pathways (Section 4.5). 5 selected hub effectors appear frequently in pathway group 1 (pathways pertaining to cell cycle control), and 11 selected hub effectors appear frequently in pathway group 2 (various signaling pathways). We expected that perturbing the 5 selected hub effectors – CDKN2A, RB1, CDK2, TP53, E2F1 – will impact cancer cells with higher Meta Gene Group 3 expressions, and perturbing the 11 selected hub effectors – AKT1, PRKCA, SHC1, RAF1, STAT3, MAPK1, SRC, MAPK3, MAPK8, MAPK14, EGFR – will impact cancer cells with higher Meta Gene Groups 1-2 expressions. To verify the predictions on the Achilles data, we further narrowed down the 16 selected hub effectors to 9

by considering the genes where the dependency profiles between RNAi and CRISPR experiments ≥ 0.1: RB1, CDK2, TP53, E2F1, AKT1, RAF1, MAPK1, MAPK14, and EGFR. We calculated the mean correlation coefficient between the Achilles dependency profile of each selected hub effector and the CCLE mRNA expression profiles of each of the 18 Gene Groups. The results are two 9×18 correlation coefficient matrices C_1 and C_2 (for RNAi and CRISPR perturbations). Since correlation coefficients between dependency and mRNA expression profiles are generally low, we rescaled C_1 and C_2 by a background distribution. We constructed two matrices B_1 and B_2 of the mean correlation coefficients between the dependency profiles of all perturbed genes (16934 and 17604 for RNAi and CRISPR data) and the 18 Gene Groups. Entries in C_1 and C_2 were converted into the CDF values P_1 and P_2 based on the background distributions in B_1 and B_2. Small CDF values (close to 0) denote negative correlation coefficients, and large CDF values (close to 1) denote positive correlation coefficients. To facilitate visualization we further converted CDF values to the range [-1,+1] by $Q_1 = (P_1 - 0.5) * 2, Q_2 = (P_2 - 0.5) * 2$. Figure 6B in the main text displays Q_1 and Q_2 for the 9 selected hub effectors on 10 Gene Groups belonging to the 3 Meta Gene Groups. RB1 and TP53 yielded strong negative values (≤ -0.8) in Gene Group 7 for both Q_1 and Q_2, and AKT1 and EGFR yielded strong negative values in Gene Group 2 for both Q_1 and Q_2. Overall, cancer cell lines with higher expressions of Meta Gene Group 3 are more dependent on RB1 and TP53, and cancer cell lines with higher expressions of Meta Gene Group 1 are more dependent on AKT1, RAF1, MAPK14, and EGFR.

5.10 Relating IHAS from TCGA and transcriptomic and epigenomic data in normal tissues

To verify IHAS in Bodymap and Roadmap data in normal tissues, we examined whether genes possessing tissue-specific expression or epigenomic states were strongly enriched in Super Modules and Gene Groups. For the Bodymap data, we calculated standard hyper-geometric enrichment p-values of the 16 sets of tissue-specific genes (Table X3) in each Super Module and Gene Group. The enrichment outcomes are sorted by both the orders of Super Module Groups and cancer types.

The Roadmap Epigenomic data was converted into a 29293×129 binary matrix R of active transcription states of 29293 probed genes over 129 probed tissues. We roughly subdivided the 129 tissues into four groups – stem, neuron, blood, and others. An element R_{ij} denotes the active transcription state of gene i in tissue type j. Each gene has a binary vector indicating its active transcription states over 129 tissues. This 129-component binary vector was reduced into a 4-component vector indicating the overall active transcription states the four tissue groups. We counted the number of genes possessing each of the $2^4 = 16$ combinatorial epigenomic states over the 4 tissue groups, sorted the combinatorial epigenomic states by the gene counts and picked the top 8 states, and categorized genes into 8 clusters (Figure 12A). Clusters 1 and 2 genes are active and inactive across all tissue types respectively. Cluster 3 genes are active in neuron tissues alone. Cluster 4 genes are active in stem cell tissues alone. Cluster 5 genes are active in both stem cell and and neuron tissues. Cluster 6 genes are active in all tissues but blood cells. Cluster 7 genes are active in all tissues but stem and blood cells. Cluster 8 genes are active in blood cells. We calculated standard hyper-geometric enrichment p-values of the 8 gene clusters in each Super Module and Gene Group.

6. Comparing IHAS with other multi-omics integration studies and databases

6.1 Comparing IHAS with other multi-omics integration studies

We compared IHAS with 5 prior studies of multi-omics integration methods: iClusters of tumors (Hoadley *et al*., 2018), immune subtypes of tumors (Thorsson *et al*., 2018), Multi-Omics Factor Analysis (MOFA, Argelaguet *et al*., 2018), Multi-omics Master-Regulator Analysis (MOMA, Paull *et al*., 2021), and tumor microenvironment subtypes (TME, Bagaev *et al*., 2021). We first qualitatively checked the presence or absence of 12 features pertaining to multi-omics data integration in IHAS and those methods. iClusters, immune subtypes and TME clustered TCGA tumors. We counted the overlaps of the reported tumor clusters with the 8 Pan-cancer Sample Groups from IHAS and calculated the enrichment p-values. MOMA and TME clustered genes. We counted the overlaps of the reported gene clusters with the 18 Gene Groups from IHAS and calculated the enrichment p-values. MOFA is a dimension reduction algorithm. For each cancer type, we applied MOFA to decompose the integrated data into 15 factors, projected samples onto the factor space, and applied k-means to cluster samples with k equals to the number of Sample Groups. MOFA sample clusters were aligned with IHAS Sample Groups by maximizing the overlap counts over all permutations of sample clusters.

6.2 Verifying IHAS results on the STRING database

STRING is a large database of known and predicted protein-protein associations of about 67 million proteins from about 14000 organisms (Franceschini *et al.*, 2013). It is widely used by biologists and bioinformaticians as a surrogate for the ground truth of protein-protein associations. STRING assigns each protein pair a confidence score of associations according to multiple types of evidence such as literature co-occurrence, high-throughput assays, and comparative genomics. We performed two enrichment analysis of the IHAS inference results on STRING. First, we checked whether effectors/regulators co-occurring in the same Super Modules tend to possess high STRING scores. We extracted 22416181 co-occurring effector/regulator pairs and sorted them by their co-occurring frequencies over the Super Modules. To assess enrichment in STRING we calculated the cumulative confidence scores (sum of the scores from the first to the current positions) and displayed the cumulative scores with respect to ranks. As a negative control we randomly sampled the same number of effector/regulator pairs and calculated their cumulative scores. The co-occurring effector/regulator pairs (blue curve) possess much higher cumulative scores than random effector/regulator pairs (red curve). Among the top 100, 1000 and 10000 co-occurring pairs, 37, 309 and 2247 possess positive STRING scores, yet only 4, 40 and 409 control pairs possess positive STRING scores.

Second, we checked whether effector-target pairs co-occurring in the same Super Modules tend to possess high STRING scores. We extracted 41334000 co-occurring effector-target pairs and assessed enrichment with the STRING database with the same procedures. The same number of randomly sampled effector and target gene pairs were extracted as the negative control. The cumulative STRING scores of the co-occurring effector-target pairs are also considerably higher than those of the random effector-target pairs, but the level of enrichment is lower than the co-occurring effector pairs. Among the top 100, 1000 and

10000 co-occurring effector-target pairs, 8, 42 and 378 possess positive STRING scores (3, 21, 252 for control effector-target pairs respectively). Recurrent effector-target pairs are less well aligned with the STRING database than recurrent effector pairs probably because there are many more targets than effectors. Consequently, some effector-target associations are likely (1) not direct protein-protein interactions or transcriptional regulation links, (2) not reported in literature since the target genes are less well studies, or (3) spurious.

References

1. E.G. Cerami *et al.*, Pathway Commons, a web resource for biological pathway data, *Nucleic Acids Research* **39**(suppl_1), D685-D690, 2011.
2. V. Matys *et al.*, TRANSFAC and its module TRANSCompel: transcriptional gene regulation in eukaryotes, *Nucleic Acids Research* **34**, D018-D110, 2006.
3. A. Kozomara and S. Griffiths-Jones, miRBase: integrating microRNA annotation and deep-sequencing data, *Nucleic Acids Research* **39**(suppl_1), D152-D157, 2011.
4. MiRTarBase: a database curates experimentally validated microRNA-target interactions, Hsu S.D. *et al.*, Nucleic Acids Research, 39, D163-D169, 2011.
5. The ENCODE Project Consortium, An integrated encyclopedia of DNA elements in the human genome, *Nature* **489**, 57-74, 2012.
6. C. Curtis *et al.*, The genomic and transcriptomic architecture of 2000 breast tumours reveals novel subgroups, *Nature* **486**, 346-352, 2012.
7. S. Madhavan *et al.*, Rembrandt: helping personalized medicine becomes a reality through integrative translational research, *Molecular Cancer Research* **7**(2), 157-167, 2009.

8. J. Barretina *et al*., The Cancer Cell Line Encyclopedia enables predictive modelling of anticancer drug sensitivity, *Nature* **483**, 603-607, 2012.
9. A. Tsherniak *et al*., Defining a cancer dependency map, Cell 170(3), 564-576, 2017.
10. F. Martinez-Jimenez *et al*., *Nature Review Cancer* **20**, 555-572, 2020.
11. J. Lamb *et al*., The connectivity map: using gene expression signatures to connect small molecules, genes, and disease, *Science* **313**(5795), 1929-1935, 2006.
12. Roadmap Epigenomics Consortium, Integrative analysis of 111 reference human epigenomes, *Nature* **518**, 317-330, 2015.
13. D. Marr, Vision: a computational investigation into the human representation and processing of visual information, San Francisco, W.H. Freeman, 1982.
14. The Biology of Cancer, Weinberg R., Garland Science, 2007.
15. ATM, CTLA4, MNDA, and HEM1 in high versus low CD38-expressing B-cell chronic lymphocytic leukemia, Joshi A.D. *et al*., *Clinical Cancer Research* 13(18), 5295-5304, 2007.
16. GTdatabase: a knowledge-base of high-throughput and curated data on cancer-testis antigens, Almeida L.G. *et al*., *Nucleic Acids Research* 37, D816-819, 2009.
17. Pan-cancer landscape of aberratnt DNA methylations across human tumors, Saghafinia S. *et al*., *Cell Reports* 25(4), 1066-1080, 2018.
18. MicroRNA-10a suppresses breast cancer progression via PI3K/Akt/mTOR pathway, Ke K. and Lou T., *Oncology Letters* 14(5):5994-6000, 2017.
19. Let-7 as biomarker, prognostic indicator, and therapy for precision medicine in cancer, Chirshev E. *et al*., *Clinical Translational Medicine*, 8:24, 2019.
20. The role of TGF-β and its receptors in gastrointestinal cancers, Luo J. *et al*., *Translational Oncology* 12(3), 475-484, 2019.

21. Methylation of SFRP2 gene as a promising noninvasive biomarker using feces in colorectal cancer diagnosis: a systematic meta-analysis, Yang Q. *et al.*, *Scientific Reports* 6:33339, 2016.
22. Overexpression of the Wilms' tumor gene WT1 in colorectal adenocarcinoma, Oji Y. *et al.*, *Cancer Science* 94(8): 712-717, 2003,
23. The miR-17/92 cluster: a comprehensive update on its genomics, genetics, functions and increasingly important and numerous roles in health and disease, Mogilyansky E. and Rigoutsos I., *Cell Death and Differentiation*, 20, 1603-1614, 2013.
24. Mir-96 promotes cell proliferation, migration and invasion by targeting PTPN9 in breast cancer, Hong Y. *et al.*, *Scientific Reports*, 6, 37421, 2016.
25. Hypermutation and microsatellite instability in gastrointestinal cancers, Yuza K. *et al.*, *Oncotarget* 8(67), 112103-112115, 2017.
26. Glutathione S-transferase polymorphisms are associated with survival in anaplastic glioma patients, Kilburn L. *et al.*, *Cancer* 116(9), 2242-2249, 2010.
27. Mir-9, a MYC/MYCN-activated microRNA, regulates E-cadherin and cancer metastasis, Ma *et al.*, *Nature Cell Biology* 12, 247-256, 2010.
28. Hsa-mir-181a and hsa-mir-181b function as tumor suppressors in human glioma cells, Shi L. *et al.*, *Brain Research* 1236, 185-193, 2008.
29. Mir-429 suppresses tumor migration and invasion by targeting CRKL in hepatocellular carcinoma via inhibiting Raf/MEK/ERK pathway and epithelial-mesenchymal transition, Guo C. *et al.*, *Scientific Reports* 8, 2375, 2018.
30. Mir-15 and mir-16 induce apoptosis by targeting BCL2, Cimmino A. *et al.*, *Proceedings of the National Academy of Sciences, U.S.A.*, 102(39), 13944-13949, 2005.

31. Y. Benjamini and Y. Hochberg, Controlling the false discovery rate: a practical and powerful approach to multiple testing, *Journal of Royal Statistical Society B*, **57**(1), 289-300, 1995.
32. D.R. Cox, Regression models and life-tables, *Journal of Royal Statistical Society B*, **34**(2), 187-202.
33. R. Peto and J. Peto, Asymptotically efficient rank invariant test procedures, *Journal of Royal Statistical Society A*, **135**(2), 185-207, 1972.
34. D. Aran *et al*., Systematic pan-cancer analysis of tumour purity, *Nature Communications*, **6**, 8971, 2015.
35. T.M. Malta *et al*., Machine learning identifies stemness features associated with oncogenic defifferentiation, *Cell* **173**(2), 338-354, 2018.
36. Cell-of-origin patterns dominate the molecular classification of 10,000 tumors from 33 types of cancer, Hoadley K.A. *et al*., *Cell* 173, 291-304, 2018.
37. The immune landscape of cancer, Thorsson V. *et al*., *Immunity*, 48, 812-830, 2018.
38. Multi-Omics Factor Analysis – a framework for unsupervised integration of multi-omics data sets, Argelaguet R. *et al*., *Molecular Systems Biology* 14:e8124, 2018.
39. A molecular master regulator landscape controls cancer transcriptional identity, Paull E.O. *et al*., *Cell* 184, 334-351, 2021.
40. Conserved pan-cancer microenvironment subtypes predict response to immunotherapy, Bagaev A. *et al*., *Cancer Cell* 39, 845-865, 2021.
41. STRING v9.1: protein-protein interaction networks, with increased coverage and integration, Franceschini A. *et al*., *Nucleic Acids Research* 41, D808-D815, 2013.
