# Supplementary material for: An integrated analysis of the cancer genome atlas data discovers a hierarchical association structure across thirty three cancer types
Source: PLOS Digit Health. 2022 Dec 20;1(12):e0000151. doi: 10.1371/journal.pdig.0000151 (PMC9931374; doi:10.1371/journal.pdig.0000151)
Supplement: Attachment — Submitted filename: Yeang_IHAS_reviews_responses.pdf [file pdig.0000151.s031.pdf]

Dear Editor and Reviewers,

Thank you for willing to consider our manuscript for publication upon major revision and sending us the constructive comments. We have considerably revised the manuscript to address all the comments raised by the editor and reviewers. To sum up, we have trimmed the length of the main text by about 30 percent, and added analysis results to present integrated views of IHAS within and across cancer types and demonstrated their relevance to cancer diagnosis and treatments. We have also validated predictions of the impacts of several selected effector genes in experimental data (the Achilles gene dependency data), though the data was not generated by our group. Below please find the point-to-point responses to each comment. To facilitate comparison we also provide a manuscript with highlighted changed text. In addition, we found the quality of the figures became rather poor after converting the manuscript into a single PDF file in the submission system. Therefore, we compressed the main figure files into Figs.zip and placed it as a supporting information file. Please ignore the figures in the combined PDF file and directly view the individual figure files in Figs.zip. We apologize for the inconvenience. Please do not hesitate contacting me if you have further questions about the manuscript.

Sincerely yours,

Chen-Hsiang Yeang
Associate Research Fellow
Institute of Statistical Science
Academia Sinica
Taipei, Taiwan

Editor

1. All reviewers recommended to revise the manuscript in a readable length while addressing various concerns on the rationales, methodology and data interpretation of this study. Detailed comments from reviewers can be found below.

   Response: We have trimmed the length of the main text (including Methods but excluding Figure and Table legends and References) by about 30 percent from 17707 to 12806 words, and basically rewritten Introduction and Discussion Sections to strengthen and focus the major arguments.

2. In particular, cancer is highly individual- and organ-specific. Please justify how the proposed global analysis will address patient/ organ-specific cancer manifestation and response to treatments, rather than making general and broad statements across multiple cancers.

Response: We've presented integrated views of various IHAS subunits in 8 specific cancer types (2 in Figure 5 and 6 in Figures S10-11 and Text S1 Section 4.5). These integrated views simultaneously manifest the enriched functions of Super Modules, selected effectors occurred in each Super Module, combinatorial expression patterns of Super Modules in Sample Groups, alignment of Sample Groups with major clinical phenotypes, and comparison of survival outcomes in each Sample Group. The multitude of information directly links the survival times and clinical phenotypes to the combinatorial expressions of Super Modules and their functional roles, and identifies the putative effector genes which may mis-regulate each Super Module.

Reviewer # 1:

The manuscript PDIG-D-22-00162 entitled "An integrated analysis of the cancer genome atlas data discovers a hierarchical association structure across thirty three cancer types" produced an Integrated Hierarchical Association Structure (IHAS) from the complete data of TCGA and compiled a large database of cancer multi-omics associations.

It is a related work in the fields of cancer omics data study. However, some issues should be addressed well before publication.

1. The targeted clinical problem is not clear, thus, readers would be not clear how to use such outcomes from this work.

Response: In the revised Introduction Section, we have stated the high-level goals: (1) Developed and implemented a data integration framework to infer associations between molecular alterations on genomes/epigenomes and transcriptomes, and between transcriptomes and clinical/molecular phenotypes, (2) Provided a compendium of these inferred associations covering 7 omics data types and across 33 cancer types, (3) Organized these associations in a hierarchical structure allowing investigations at multiple levels of details, (4) Validated IHAS in a wide range of external datasets. These goals enable IHAS to tackle the following clinical problems: (1) Categorizing patients in terms of molecular signatures of effector alterations and target gene expressions. We have shown that IHAS-induced categories were aligned with most clinical phenotypes reported in TCGA (Figures 4B-C, 5, S7, S10-11, Tables 5 and S5B), (2) Selecting targeted genes or drugs for precision cancer treatments based on the patterns of effector occurrences and target gene expressions (Figures 6, 9B-D), (3) Demonstrating that the directions of associations between survival times and Gene Groups vary with cancer types (Figure 4D), hence eliciting caution when using transcriptional biomarkers to predict prognostic outcomes. We have also addressed the clinical implications in the Discussion Section.

2. Authors stated that current existing methods would not organize the associations in a hierarchical structure. It is necessary to display the benefit of hierarchical structure. Indeed, the current results have not shown the improvement from hierarchical structure (e.g. Figure 3) and extensive external data (e.g. Figure 4). They are still general heatmap structure and visualization like conventional method, thus there are not novel information produced or available.

Response: We thank the reviewer's comment about the benefits of hierarchical structure. To justify those benefits we elaborated the information gain by traversing up or down along the hierarchy (Subsection *Benefits of the hierarchical structure* under *Functional characterization of IHAS*). The upward information gain is more obvious, as a higher level subunit unifies multiple lower level subunits and thus contains information not covered by individual lower level subunits. Conversely, the downward information gain offers details in lower level subunits but ignored in higher level subunits. Figure S6 summarizes the downward information gain at three levels: (1) some Super Modules are enriched with the functions not in the higher level Gene Groups and Meta Gene Groups, (2) some Association Modules possess distinct expression profiles than those of the Super Modules they belong to, (3) some Association Models share a common effector but also possess other distinct effectors, and they tend to have more disparate expression profiles. Figures 3-4 (in the previous version) just visualize the partial information at each level of IHAS but certainly do not represent the multi-level complex relations of IHAS subunits. They are used to elucidate the content of IHAS at each level.

3. In fig8. The validation on GSE data is not significant. There should be lots of evidences with significant biological or biomedical meaning.

Response: Figure 8 is used to illustrate how the combinatorial expression patterns and survival curves of Sample Groups are aligned between TCGA and GEO data. The log-rank p-values do not need to be significant as long as the order of Sample Group survival curves is preserved between TCGA and GEO datasets. In this example, the survival curves of TCGA-LUAD and GSE68465 follow the order of groups 1 and 2 > group 3 > group 4, and the four Sample Groups reflect a decreasing level of differentiation. The insignificant log-rank p-values in the two datasets are likely due to the smaller differences between Sample Groups 2 and 3. A comprehensive validation on GEO data is reported in Table S9.

4. In particular, this is a work on multi-omics in pan-cancer, however, there are not enough results and discussions on the contribution from multi-omics analysis, e.g. shared or complementary information from different biological levels.

Response: We reported several aspects about multi-omics comparison. In Table S1A, we reported the numbers of Association Modules pertaining to each type of molecular alterations in each cancer type and all cancer types together. SNP modules are scarce despite the large number of SNPs in the data. Mutation and DNA methylation modules are the most and the second most abundant. Other types of molecular alterations comprise similar numbers of modules. In Table S10 and Figure S13, we also demonstrated that associations pertaining to CNV, mutations and DNA methylations are more reproducible than those of microRNA expressions and protein phosphorylations.

5. There are not definite calculation model and measurements in main text. I only saw descriptions of concepts. More solid details of different so-called modules should supply their analysis and biology hypothesis.

Response: The detailed descriptions of data collection and processing and model inference are reported in Supplementary Text S1 (Sections 1-2). In particular, the formulation and inference of Association Models from TCGA data are reported in Supplementary Text S1 Section 2.1. Since the length of the paper is already criticized by most reviewers, we think supplying more technical information in the main text will probably raise more criticism and confuse readers. We did elucidate inference of the IHAS subunits in the Subsection *Overview of integrated analysis and validation on pan-cancer omics data* and Method Section. We also provided some detailed information of some Association Models, Association Modules, Super Modules, and Super Module Groups in Figures 1 and S4. In particular, in Subsection *Summary of IHAS from TCGA* of the Results Section, Figure S4, and Supplementary Text S1 Section 2.3, we depicted selected Association Modules in four Super Modules of BRCA, COAD, LGG and LIHC.

6. The key is the database and useful web-server for experts from different fields. But, in current form, there is not detail database and web introduced and discussed in main text. It is necessary to provide the complete database for public domain.

Response: We have moved the Webpage documents (Supplementary Data) under an URL https://www.stat.sinica.edu.tw/IHAS/ for public access. We have also added descriptions about Supplementary Data in the beginning of the Results Section to promote its usage. Building a full-fledged database is beyond the scope of this paper as it requires extensive extra efforts and the technical information of database construction and interface is independent of the concepts and biological findings of IHAS. Therefore, we deposited the information primarily in figures and tables, but also provide a simple search function to allow users to filter the content of Super Modules by gene names.

Reviewer #2:

In this paper, the authors present a pan-cancer analysis of TCGA data for hierarchical association structure across multiple cancers.

1. The major concern is what are the main findings from the integrative data analysis. What are their indicators for cancer studies? How to justify these findings?

Response: Rich biological findings are derived from IHAS. In the Discussion Section we listed several major findings from the pan-cancer analysis of IHAS and discussed their clinical implications. First, at a high level the transcriptomic variations of most tumors are reduced to the combinatorial patterns of three dominant biological processes (Meta Gene Groups): immune response, development and metastasis, and cell cycle control, as well as several other major processes (Gene Groups) such as translation and respiration. These combinatorial expression patterns are aligned with the majority of clinical and molecular features of cancers. Second, the combinatorial expression patterns of of IHAS subunits provide informative guidelines for targeted treatments. Cancer cell lines with elevated Meta Gene Group 1 or 3 expressions are differentially sensitive to distinct sets of drugs (Figure 9B) and differentially dependent on perturbing effector genes on two sets of pathways (Figure 6B). Third, although the combinatorial expression patterns of Meta

Gene Groups and Gene Groups are ubiquitous across cancer types, their relations with patients' survival times vary with cancer types. This property elicits caution when using transcriptional biomarkers to predict prognostic outcomes. These findings were justified/validated by analysis of external data. For instance, from analysis of TCGA data we found hub effectors in two groups of pathways impacted distinct Meta Gene Groups (Figure 6A). We validated this prediction in Achilles data by showing cancer cell lines with higher expressions in a Meta Gene Group were more dependent on perturbing the predicted effector genes (Figure 6B).

2. This paper provides a resource of building the complicated associations in a hierarchical structure (across multiple data types and across multiple cancer types). It is expected to clarify some concrete conclusions for this hierarchical structure.

Response: Similar to our responses to question 1, in the Discussion Section we have listed major and concrete findings and discussed their clinical implications. In addition to the aforementioned three findings, IHAS also provides integrated views of multiple aspects of IHAS information in specific cancer types (Figures 5 and S10-11).

3. As for so many cancer types, it is good to present the common features in these associations. For cancer specific features, it is also suggested to present in a rational way.

Response: We have already organized these associations in a hierarchical structure. Common features of the IHAS subunits at one level are the IHAS subunits at the next higher level. For instance, Super Modules across cancer types sharing a large portion of target genes are grouped together as a Super Module Group, and genes that co-appear in the same set of Super Module Groups are grouped together as a Gene Group. Cancer type specific features are the lower level IHAS subunits including Association Models and Modules, Super Modules, and Sample Groups. In addition, we also listed some unique features which are not shared across cancer types (Table S2C, Figures 5, S6, S10-11). For instance, in BRCA the Super Modules enriched with estrogen response pathways play vital roles in breast cancer phenotypes (Figure 5A).

4. This paper is very long and it is hard to grasp the main idea for the reader's perspective. It is possible to summarize the main findings more clearly.

Response: We thank the reviewer's comment. Other reviewers also criticized the length of the manuscript. The paper is by nature long and not easy to follow as it tackles a very large dataset with rich information and complicated interactions. In the revised manuscript we have significantly reduced its length by about 30%. We provided summary information in several parts of the paper. In Figure 2, we visualized the overview of the integrated analysis and validation framework. In Discussion we listed several major findings drawn from pan-cancer analysis and their clinical implications. Each figure or table summarizes the results from one type of analysis. For instance, Figure 4 and Table 5 summarize the alignment of IHAS with clinical phenotypes, Figures 7-12 and Table S9 summarize validation outcomes with external datasets.

5. The cancer types are diverse as described. Is it possible to cluster them into several groups and summarized the common features in the hierarchical structures.

Response: Prior studies have already clustered TCGA samples based on multi-omics data and found that most sample clusters correspond to groups of cancer types (Hoadley *et al.*, 2018, Thorsson *et al.*, 2018). Our results partially agree with these studies (Figure 4D, Table S13BCF). However, we find it is better to represent the pan-cancer data as a decomposition of common and unique patterns rather than clustering cancer types. Samples are grouped together when they share similar combinatorial patterns, and the groups of samples are not necessarily correlated with cancer types. For instance, the Pan-cancer Sample Groups are demarcated by the expression patterns of three Meta Gene Groups but not highly correlated with cancer types.

6. It is good to compare the present method to the other omics integration methods. How to evaluate the results by different data integration methods? Thus, the authors need present their work more clearly.

Response: We dedicated Subsection *Comparison of IHAS with other multi-omics integration studies and databases* to compare IHAS with other omics integration methods. As pointed out in the beginning of the subsection, there is no single yardstick to measure the performance of these methods since they possess specific objectives, assumptions, approaches and focused biological processes. We compared IHAS with 5 other methods by two means. Qualitatively we listed the presence or absence of 12 features in these methods (Table S13A) and demonstrated that only IHAS possessed all 12 features. Quantitatively we calculated the overlap and p-values of IHAS with the results reported by other methods and showed that IHAS results were more similar to immune and TME subtypes than iClusters, MOFA and MOMA (Table S13B-G). We also validated IHAS inference results on the STRING database of molecular interactions by showing that associations occurring more frequently in IHAS also tend to possess more confident interactions (Figure S15 and Table S13HI).

Reviewer #3:

This paper studies the hierarchical correlation structure of 33 cancer types, but there are problems such as a lot of repetition and no emphasis. In addition, there are also big problems in the schematic diagram, as follows:

1. The introduction contained so many descriptions about previous studies, and lacked comparisons and conclusions.

Response: We thank the reviewer's comment. We have re-written the Introduction Section to make it better fit the aims and structure of the paper. In brief, Introduction has the following logic flow. (1) Relations of molecular alterations in cancer are important. (2) Large-scale projects such as TCGA and ICGC already probe molecular alterations, yet reconstruction of their relations is still challenging. (3) List the four goals of our work. (4) List previous methods and state that they do not simultaneously fulfill the four goals.

2. In the results section, the biological explanations were missing, and many concepts were mentioned so many times. The core conclusions were also missing.

Response: Given the size and complexity of TCGA data, we cannot provide comprehensive biological interpretations to the entire inference results. Instead we gave summary biological interpretations to the IHAS results across cancer types and in a few cancer types. In specific cancer types, we reported integrated views of BRCA, COAD and 6 other cancer types (Figures 5 and S10-11, Supplementary Text S1 Section 4.5) and demonstrated that the combinatorial expressions of several functional groups determined the molecular subtypes. Across cancer types, we reported that the combinatorial expressions of common subunits (Gene Groups, Meta Gene Groups) were aligned with pan-cancer clinical phenotypes (Figure 4A-C), and the effectors in distinct sets of pathways impacted different Meta Gene Groups (Figure 6). We have significantly reduced the manuscript length by about 30% and removed the redundant statements. Most redundant statements were in the Discussion Section. We have re-written Discussion to address the main conclusions and clinical implications of IHAS.

3. There are so small front label for Figure 1, and the workflow of overall design was confusing.

Response: Figure 2 (Figure 1 in the previous version) is very busy because it encapsulates all analysis steps in the work. Each block except the top row (data processing) appears as figures or tables in the paper. They serve as thumbnails for each step but are not meant to convey the detailed information about the content. Thus readers do not need to read the small labels in those blocks. The top blocks provide essential information about the data types in the processing, thus we enlarged their font sizes. The blue arrows indicate the prerequisite relations of those steps. For instance, the three steps of pan-cancer characterization of IHAS all require Super Module Groups and Gene Groups. To better explain the workflow, we also provided Figure S1 to elucidate the architecture and information flows of the IHAS inference machine.

4. The Figure 2 should be totally revised and the label was too small.

Response: We have moved Figure 2 to Figure S1 and enlarged all font sizes.

5. There were so many examples and no biological evaluations.

Response: We have either cut or moved most examples to Supplementary Text S1. We performed biological evaluations in four aspects. First, we assessed the functions of target genes and effectors in multiple levels of IHAS subunits (Tables 3, S2, S4). Second, we aligned Sample Groups and Pan-cancer Sample Groups with clinical phenotypes within and across cancer types (Tables 5 and S5B, Figure 4). Third, we found hub effectors in two groups of pathways impacted distinct Meta Gene Groups (Figure 6A). Fourth, we validated this prediction in Achilles data by showing cancer cell lines with

higher expressions in a Meta Gene Group were more dependent on perturbing the predicted effector genes (Figure 6B).

6. The authors must provide the website or link for users, which was important for research.

Response: We have moved the Webpage documents (Supplementary Data) under an URL https://www.stat.sinica.edu.tw/IHAS/ for public access.

7. Some parts appear many times in the text, such as "three aspects of molecular changes in cancer", suggesting to remove unnecessary parts.

Response: We have significantly reduced the manuscript length by about 30% and removed the redundant statements. Most redundant statements were in the Discussion Section. We have re-written Discussion to remove these redundant statements.

Reviewer #4:

In this study, Tiong et al. built an Integrated Hierarchical Association Structure (IHAS) between molecular alterations on genomes/epigenomes and variations on transcriptomes in 33 cancer types. They justified the biological relevance and clinical utility of IHAS by characterizing its functional properties, aligning combinatorial expressions of IHAS subunits with phenotypes, and validating IHAS in a wide range of external datasets. IHAS seems valid and the results they show are promising. However, there are existing some specific problems.

1. I did not see the Web page of IHAS. Is it possible to use IHAS through its web interface? Please make it publicly accessible.

Response: We have moved the Webpage documents (Supplementary Data) under an URL https://www.stat.sinica.edu.tw/IHAS/ for public access.

2. The manuscript is too long, and it is not easy to follow the information disclosed by IHAS. The authors may split it into two (one is about IHAS framework and the other is the discovery from IHAS).

Response: We thank the reviewer's suggestion about splitting the paper into two. Splitting has the merits of controlling the paper length but is difficult to implement since the IHAS framework and biological discovery are not quite separable. Nowadays a dry-lab analysis paper requires biological validation to get published, and the analysis results are closely tied to the IHAS framework. Alternatively, we have trimmed the length of the main text (including Methods but excluding Figure and Table legends and References) by about 30 percent, and basically rewritten Introduction and Discussion Sections strengthen and focus the major arguments.

3. Besides validated IHAS in more than 300 external datasets, could the authors validate several discoveries using biomedical experiment?

Response: We thank the reviewer's suggestion. Our group does not have resources to perform wet-lab experiments. As a proxy for carrying out experiments on our own, we have validated predictions inferred from TCGA data on an external data of gene perturbation. From analysis of TCGA data we found hub effectors in two groups of pathways impacted distinct Meta Gene Groups (Figure 6A). We validated this prediction in Achilles data by showing cancer cell lines with higher expressions in a Meta Gene Group were more dependent on perturbing the predicted effector genes (Figure 6B).

Reviewer #5:

The authors propose an integrative analysis framework, namely IHAS, to combine 7 multi-omics data types in 33 cancers of TCGA to discover hierarchical association structure and insightful biological findings. The results are validated by large-scale external data. The concept of finding hierarchical association structure is appealing and innovative for the complex problem. But there are concerns of the approach, results and validation, which are discussed below.

1. The target-effector association and hierarchical strategy are biologically reasonable and good approaches. But the method produces massive amount of information that are difficult to follow and the findings generally become descriptive. Some reduction approaches in omics types, cancer types, omics features should be seriously considered. The authors ambitiously include all 7 omics types and all 33 cancers but it is reasonable to imagine that some omics types and some cancer types have weaker signals to be filtered.

Response: We thank the reviewer's positive comments. The purpose of IHAS is to reduce the massive amount of information in all the associations into a hierarchical structure. At the top level, there are only three Meta Gene Groups pertaining to immune response, development, and cell cycle control, and eight Pan-cancer Sample Groups generated by the binary combinations of the Meta Gene Groups. At the next two levels there are manageable numbers of Gene Sets (18) and Super Module Groups (17), Super Modules (217), and Sample Groups (228). At the lower levels there are many more Association Modules and Models. Therefore, in the paper we focused discussions and validations on the high-level subunits across cancer types and highlighted those of a few specific cancer types (such as breast and colon cancers). The large numbers of Association Modules and Models and the diverse effectors in Super Modules should be viewed as a compendium just like TCGA (although the entities are derived from the TCGA data). Users who are interested in specific cancer types or omics types can narrow down their scope accordingly. We agree with the reviewer that some omics types and cancer types have weaker signals and have presented relevant evidence about this observation. We have demonstrated that associations pertaining to CNV, mutations and DNA methylation were more reproducible than other types of associations in CCLE data (Figures 9A and S13, Table S10AB). Similarly, some cancer types (such as LAML and UVM) possessed far fewer Association Modules than other cancer types (such as BRCA

and COAD) (Table S1A). However, rather than applying explicit filters to association signals we kept all associations but also reported their strength (χ^2 and permutation p-values). Users may discard weaker associations according to those scores.

2. The association model Equation (1) is confusing. Which is independent and which is dependent variable? What is f_i(x_i) in the equation? The authors refer to it as logistic regression model. If y is the target gene expression, how can it be a logistic regression model?

Response: We apologize for the confusion and have modified the text to better explain equation 1. It resembles a logistic regression model but is not exactly it, since in standard logistic regression the independent variables $\boldsymbol{x}$ are continuous and dependent variable y is discrete. Instead, in our model $\boldsymbol{x}$ (effector alterations) can be continuous (such as CNV or DNA methylation) and discrete (such as mutations and SNPs), and y (target gene expression) is continuous. Since inference for a hybrid model is more difficult, we considered $p(y|\boldsymbol{x})$ where $\boldsymbol{x}$ and y are all discrete. We renamed it an exponential family model. To preserve the information in the continuous measurements (of gene expressions, CNV, DNA methylations, etc), we further introduced a probabilistic quantization procedure to convert the continuous measurements (e.g., normalized gene expression values) into probabilities of discrete states (e.g., probabilities of up/down regulation or no change). We specified the possible feature functions $f_i(x_i)$ in Supplementary Text S1 Section 2.1.1. If x_i is a quantized numeric variable (CNV, DNA methylations, etc), then only two functions are allowed: $f_i(x_i) = x_i$ and $f_i(x_i) = -x_i$ denote that the effector (x) activates or represses the target gene expression (y) respectively. If x_i is a categorical variable (mutation and SNP), then twelve functions in Table X4 are allowed.

3. In the section "Functional characterization of IHAS", the authors characterize three functional properties of IHAS subunits and say that Gene Groups are primarily enriched with the three functional categories: immune response, development, and cell cycle. However, this is not surprising since the hierarchical clustering algorithm for Gene Groups already involves the enrichment patterns of six functional categories including immune response, development, and cell cycle as criteria. So, it cannot serve as the evidence that the Gene Groups by IHAS are good/biologically meaningful.

Response: We apologize the misunderstanding caused by our presentation. We used these six functional categories in the stopping criteria for hierarchical clustering of Super Modules because we observed that the majority of Super Modules were enriched with some of these six functional categories. The hierarchical clustering algorithm is a systematic approach to quantify this observation. Since it is a bit cumbersome to state the whole reasoning process (Frequent enrichment of six functional categories in most Super Modules → Use the six functional categories in the stopping criteria to cluster Super Modules to Super Module Groups → Decompose Super Module Groups into combinations of Gene Groups), we skipped the first step and hence created an impression that the six functional categories were explicitly imposed instead of arising from the data. We have added a sentence explaining why using these six functional categories in describing the method of generating Super Module Groups.

4. In the section “Alignments of IHAS with clinical phenotypes”, the definition of concentration coefficients is ambiguous, even in the supplementary. Since the concentration coefficient is the criteria to evaluate the aligning of Sample Groups with the clinical phenotypes, it might be better to further interpret the concentration coefficient to justify the conclusion that the combinatorial expression patterns of IHAS subunits define most clinical and molecular phenotypes in TCGA. Similarly, in the section “Pan-cancer Sample Groups are aligned with pan-cancer phenotypes”, the authors state that “Meta Gene Groups are closely aligned with these pan-cancer features.”, but there is no quantitative correlation of Meta Gene Group with pan-cancer feature in Table 5, and Figure 6B does not clearly show strong correlation. So, they may report the correlation and make it solid.

Response: We thank the reviewer’s suggestion. To better explain concentration coefficients we have added an example of aligning ACC Sample Groups with DNA methylation subtypes to elucidate how it is calculated in Subsection *Alignments of IHAS with clinical phenotypes – Sample Groups are aligned with over 80% of clinical features within cancer types*. We also calculated the correlation coefficients between sample purity and median Meta Gene Group 1 expressions (-0.5029) and between RNA stemness and median Meta Gene Group 3 expressions (0.5101). Considering the median expression profile was aggregated from thousands of genes, these correlation coefficients are indeed very high.

5. In the section “Validation on external datasets”, the authors state that “In the first part, we manifested the veracity of IHAS by indicating that various aspects of IHAS were preserved in external datasets. They include the expression coherence of the target genes in the same subunits (Super Modules, Gene Groups, Meta Gene Groups), associations between effector alterations and target gene expressions, combinatorial expression patterns of Super Modules and Sample Groups, and associations between IHAS subunits (Super Modules target genes, Gene Group members) and clinical features (survival times, molecular subtypes, etc).” However, it is not clear which of the following subsections assessed the corresponding aspect they mentioned. It seems that they only evaluated the coherent expressions of Super Modules in external data but not Gene Groups and Meta Gene Groups. In addition, there is no clear conclusion about the performance of IHAS on the external datasets or how reliable the IHAS is.

Response: In the beginning of Subsection *Validation on external datasets*, we have specified which aspects were assessed by listing the external datasets used in the three parts: METABRIC, REMBRANDT, GEO and CCLE in the first part, CCLE drug response and Achilles in the second part, and Bodymap and Roadmap in the third part. We reported expression coherence of Super Modules in METABRIC (Table S7), REMBRANDT (Table S8), and GEO (Table S9), and expression coherence of Gene Groups in CCLE (Table S10A), Bodymap (Table S11A) and Roadmap (Table S12B). The reason for this arrangement is that METABRIC, REMBRANDT and GEO datasets are cancer type specific thus more appropriate for Super Modules, while CCLE, Bodymap and Roadmap datasets are pan-cancer thus more appropriate for Gene Groups. The expression coherence of the three Meta Gene Groups will be very similar to that of Gene Groups 1-3, 4-6, and 7, 8, 10, 12. Since the IHAS inference results are complex, it

is hard to give a one-sentence conclusion about validation performance. But there are several main findings: (1) the combinatorial expression patterns of IHAS subunits are generally preserved in other tumor datasets, (2) the inferred associations are more preserved for CNV, mutation and DNA methylation effectors and less preserved for microRNA expressions and protein phosphorylations, (3) Meta Gene Groups 1-2 are less coherently expressed than Meta Gene Group 3 in cancer cell lines, (4) the predicted impacts of selected effectors on Gene Groups are validated by drug response and gene dependency data, (5) some IHAS subunits stem from the expression patterns of the normal tissues of origin.

6. In the section "Comparison of IHAS with other multi-omics integration studies and databases", the second paragraph mentioned "12 features" as a criterion of comparing IHAS with the reference methods, but there is no explanation/definition for 12 features.

Response: We have added definitions of the 12 features in the second paragraph of Subsection *Comparison of IHAS with other multi-omics integration studies and databases*.

7. About the supplementary, some algorithms of IHAS are ambiguous. For example, the algorithm of clustering Association Modules and samples is not clear. It would be easy to read if the authors could use mathematical formula/representation.

Response: We thank the reviewer's suggestion. We have added mathematical notations in Section 2.3 (Super Modules and Sample Groups) of Supplementary Text S1 to explain the algorithm of clustering Association Modules and samples into Super Modules and Sample Groups.

8. The paper is unusually long but the presentation is very difficult to follow. The "Overview" section and Figure 1 are not very helpful. Particularly, after reading them multiple times, it's still difficult to understand the meaning of models, modules, super modules, super module groups and gene groups in this paper. Figure 2 is not legible even on a large monitor. Figure 3A-3C start to provide information easier to digest. But then Figure 3D is difficult to follow. The readers need to go back-and-forth in the result and method sections to guess and understand the method.

Response: We thank the reviewer's comment. After re-examining the manuscript we agree that it is too long and the logic flow is not easy to follow. Therefore, we substantially revised the manuscript to address these concerns. First, we have trimmed the length of the main text by about 30%. Second, in the old version the Overview Subsection starts with a list of all IHAS subunits followed by an example. To improve understanding we have swapped the order by first introducing an example of IHAS subunits and then describing the IHAS inference and validation framework. Third, in the old version the concepts and results of IHAS subunits are mixed. In the new version we have first described the concepts and then in another subsection summarized the results. Fourth, we have moved the figure of the architecture of the IHAS inference machine (Figure 2 in the old version) to Figure S1 in the new version, and have enlarged font

sizes. Fifth, the old Figure 3D is difficult to follow because we didn't explain the membership occurrence matrix when presenting the figure in the old version. In the new version we have explained the heat map of the membership occurrence matrix.

9. This work does not fit to PLOS Digital Health and can fit better to PLOS Comp Bio, PLOS Genetics or PLOS ONE.

Response: As PLOS Digital Health is still a young journal and the discipline of Digital Health is still evolving, I think it is a good strategy to include articles of a wide scope related to digital health. IHAS has strong clinical implications about diagnosis for clinical phenotypes, survival and treatment outcomes, and selection and design of targeted treatments. Thus I think it is relevant to the broad scope of this journal.
