# Supplementary material for: An integrated analysis of the cancer genome atlas data discovers a hierarchical association structure across thirty three cancer types
Source: PLOS Digit Health. 2022 Dec 20;1(12):e0000151. doi: 10.1371/journal.pdig.0000151 (PMC9931374; doi:10.1371/journal.pdig.0000151)
Supplement: Attachment — Submitted filename: Yeang_IHAS_reviews_responses2.pdf [file pdig.0000151.s032.pdf]

Dear Editor and Reviewers,

Thank you very much for sending us the comments about our revised manuscript and willing to consider it for publication upon the second revision. We are grateful for the positive comments regarding our efforts to address the concerns about the previous version of the manuscript. There are a few minor comments raised by reviewers 1 and 2. We have modified the manuscript to address these comments. Below please find the point-to-point responses to each comment. Please do not hesitate contacting me if you have further questions about the manuscript.

Sincerely yours,

Chen-Hsiang Yeang
Associate Research Fellow
Institute of Statistical Science
Academia Sinica
Taipei, Taiwan

Journal Requirements:

1. Please review your reference list to ensure that it is complete and correct. If you have cited papers that have been retracted, please include the rationale for doing so in the manuscript text, or remove these references and replace them with relevant current references. Any changes to the reference list should be mentioned in the rebuttal letter that accompanies your revised manuscript. If you need to cite a retracted article, indicate the article's retracted status in the References list and also include a citation and full reference for the retraction notice.

Response:

We have added two references which we cited but did not list in the references of the previous version (Kikuchi *et al*., 2011 and Furukawa *et al*., 2006), and corrected a few typos in the revised references. We have examined all the references and ensured no cited papers were retracted.

2. Please insert an Ethics Statement at the beginning of your Methods section, under a subheading 'Ethics Statement'. It must include:

a) The name(s) of the Institutional Review Board(s) or Ethics Committee(s)

b) The approval number(s), or a statement that approval was granted by the named board(s)

c) (for human participants/donors) - A statement that formal consent was obtained (must state whether verbal/written) OR the reason consent was not obtained (e.g. anonymity). NOTE: If child participants, the statement must declare that formal consent was obtained from the parent/guardian.

Response: We have added an Ethics Statement at the beginning of the Methods section.

Reviewer # 1:

1. Authors have responded to all my concerned questions. There is only few remaining issue.

For the question about "details of different so-called modules should supply their analysis and biology hypothesis", authors replied "Since the length of the paper is already criticized by most reviewers, we think supplying more technical information in the main text will probably raise more criticism and confuse readers."

Indeed, IHAS is not a general model, thus, the current main figure and text didn't provide enough information for me and readers in different fields. The current figure 1 is too simple to start the paper. In current form, Figure 1 should, at least, supply a clear demonstration of the key characteristics of IHAS, including: the hierarchical association structure (e.g. in a general tree format), the expression pattern corresponding to different module types, the association between modules and clinical indices (e.g. survival), and the shared or complementary expression pattern of one module in different omics data. Of course, a few key definition and formula of these characteristics can also be shown in Figure 1.

Response: We thank the reviewer's clear suggestions about Figure 1, and have modified it according to these suggestions. Three extra panels are added to the figure, and the existing panels are better annotated. The revised Figure 1 includes the following panels to illustrate IHAS: (A) The Association Model of target gene E2F1 in BRCA. All the effectors and the target gene are annotated. The target gene E2F1 and the selected effector trans-acting chr8q CNV are marked by red, (B) The Association Module of trans-acting chr8q CNV in BRCA. The selected effector trans-acting chr8q CNV and target gene E2F1 are marked by red, (C) A BRCA Super Module encompassing the selected effector trans-acting chr8q CNV and target gene E2F1. Variations of all effectors across 1201 samples are displayed. The types of multi-omics assays appeared in the Super Module are annotated, (D) An excerpt of the gene membership occurrence matrix of Super Modules pertaining to the selected BRCA Super Module in panel C and its enriched Gene Groups. The submatrix is displayed by a heat map, (E) The combinatorial expressions of all BRCA Super Modules and Sample Groups, and the alignment of the sorted samples with PAM50 subtypes and survival times, (F) The combinatorial expressions of three Meta Gene Groups and two Pan-cancer Sample Groups across multiple cancer types, and the alignment of sorted samples with several pan-cancer phenotypes including cancer types, sample purity, DNA and RNA stemness, (G) An overview of relations between subunits of IHAS. Although subunits do possess hierarchical inclusion relations (e.g., a Module consists of multiple Models, and a Super Module consists of multiple Modules), it is a bit cumbersome to represent these relations

by a multi-level tree. Instead we represent a type of subunits as a node and draw a dependency graph of the subunits.

Reviewer # 2:

1. The authors have addressed my comments. A minor comment is about the figures. They are not clear and hard to recognize.

Response: As we explained in the response letter of the first-round reviews, the quality of figures became deteriorated after converting the manuscript into a single PDF file in the submission system. Therefore, we compressed the main figure files into Figs.zip and placed it as a supporting information file. Please ignore the figures in the combined PDF file and directly view the individual figure files in Figs.zip. If the paper is accepted, we will provide the high-resolution figures to the editorial team to ensure that their quality is not compromised in the publication.

2. The method section need contain more introduction to the multi-level/hierarchical issues in data integration.

Response: We thank the reviewer's constructive comments. We have added a subsection "Hierarchical relations of IHAS subunits" in the beginning of the Methods section to give an overview of the relations of IHAS subunits and illustrated these relations in the revised Figure 1G. Vertical unidirectional lines denote the hierarchical inclusion relations (e.g., a Module contains multiple Models, and a Super Module contains multiple Modules). Horizontal bidirectional lines denote the combinatorial relations of subunits at the same levels (e.g., Super Modules and Sample Groups of the same cancer type form the combinatorial expression patterns of certain biological processes in certain subtypes). These hierarchical relations are also illustrated in the beginning of the Results section and Figure 1 legend.
